# Exploring the Leaf Beetle Fauna (Coleoptera: Chrysomelidae) of an Ecuadorian Mountain Forest Using DNA Barcoding

**DOI:** 10.1371/journal.pone.0148268

**Published:** 2016-02-05

**Authors:** Birthe Thormann, Dirk Ahrens, Diego Marín Armijos, Marcell K. Peters, Thomas Wagner, Johann W. Wägele

**Affiliations:** 1 Zoological Research Museum Alexander Koenig, Bonn, Germany; 2 Museo de Colecciones Biológicas MUTPL, Departamento de Ciencias Naturales, Universidad Técnica Particular de Loja, Loja, Ecuador; 3 Department of Animal Ecology and Tropical Biology, Biocenter, University of Würzburg, Würzburg, Germany; 4 Abteilung Biologie, Institut für Integrierte Naturwissenschaften, Universität Koblenz–Landau, Koblenz, Germany; Consiglio Nazionale delle Ricerche (CNR), ITALY

## Abstract

**Background:**

Tropical mountain forests are hotspots of biodiversity hosting a huge but little known diversity of insects that is endangered by habitat destruction and climate change. Therefore, rapid assessment approaches of insect diversity are urgently needed to complement slower traditional taxonomic approaches. We empirically compare different DNA-based species delimitation approaches for a rapid biodiversity assessment of hyperdiverse leaf beetle assemblages along an elevational gradient in southern Ecuador and explore their effect on species richness estimates.

**Methodology/Principal Findings:**

Based on a COI barcode data set of 674 leaf beetle specimens (Coleoptera: Chrysomelidae) of 266 morphospecies from three sample sites in the Podocarpus National Park, we employed statistical parsimony analysis, distance-based clustering, GMYC- and PTP-modelling to delimit species-like units and compared them to morphology-based (parataxonomic) species identifications. The four different approaches for DNA-based species delimitation revealed highly similar numbers of molecular operational taxonomic units (MOTUs) (n = 284–289). Estimated total species richness was considerably higher than the sampled amount, 414 for morphospecies (Chao2) and 469–481 for the different MOTU types. Assemblages at different elevational levels (1000 vs. 2000 m) had similar species numbers but a very distinct species composition for all delimitation methods. Most species were found only at one elevation while this turnover pattern was even more pronounced for DNA-based delimitation.

**Conclusions/Significance:**

Given the high congruence of DNA-based delimitation results, probably due to the sampling structure, our study suggests that when applied to species communities on a regionally limited level with high amount of rare species (i.e. ~50% singletons), the choice of species delimitation method can be of minor relevance for assessing species numbers and turnover in tropical insect communities. Therefore, DNA-based species delimitation is confirmed as a valuable tool for evaluating biodiversity of hyperdiverse insect communities, especially when exact taxonomic identifications are missing.

## Introduction

Worldwide, natural habitats are disturbed and destroyed at alarming rates which results in a massive loss of species [1]. Especially in hyperdiverse habitats like tropical rainforests, many species are in danger of extinction before even being discovered [2,3]. This implies that biodiversity assessment needs to be accelerated which should obviously include not only enhanced sampling but also to complement traditional methods of species delimitation with more universal and faster approaches [4–7]. Therefore, DNA-based methods are increasingly employed with success for species delimitation, but their robustness and reliability is still matter of debate [8,9]. Therefore, beyond theoretical exploration of parameter space [10–13] also more empirical studies are needed that survey the performance of accelerated species delimitation approaches [14].

Knowledge of species numbers, on local as well as on global scale, is important for providing a reference point to estimate biodiversity loss [15]. Although biodiversity can be assessed at different levels of classification, the significance of the species as a biological unit is widely recognized. Species are the 'currency' of conservation biology [16]. Species richness and species turnover are important parameters of community structure [17,18].

While debates on how a species should be defined resulted in different competing species concepts [19] these may lead to different species numbers and have potential impact on decisions of conservation management [16]. However, since most DNA-based delimitation methods are founded on the phylogenetic species concept this study is focused on the operational performance of different species delimitation methods for field collections of highly diverse insect assemblages.

Beetles play a crucial role in efforts to estimate the total number of species on Earth [20,21] because they are extremely rich both in functionality and species numbers, making up about one-quarter of all species on Earth [22]. Leaf beetles (Coleoptera: Chrysomelidae) are with ~37,000 described species in more than 2,000 genera one of the largest groups of beetles [23–25]. Their herbivorous feeding makes them attractive for ecological research [26–28] as diversity of herbivorous insects is strongly linked with that of plants [29]. The degree of host specificity of herbivorous beetles has played a fundamental role in estimates of world’s species diversity [21,30]. Leaf beetles represent a large part of the herbivorous insect fauna in many biomes (e.g. tropical rainforest) [31–33] and they are easily noticed and collected even by non-specialists [34]. Knowledge of their communities is essential to understand plant-herbivore-interactions [34]. However, while most studies rather focus on taxonomic or phylogenetic issues [35–40], so far there have been only few detailed studies on the diversity and turnover of leaf beetle communities in Neotropical ecosystems (e.g. [34,41–44]). The Neotropical beetle fauna is comparatively poorly studied [23] and a much higher actual diversity than that recorded in the current literature should be assumed [44].

Extreme species richness associated with the difficulty of sorting and identifying a poorly known fauna presents a methodological challenge for large-scale biodiversity studies [45,46]. DNA-based tools have been shown to be a valuable and effective approach for biodiversity assessment when the diversity of unknown samples is too large to be handled with traditional taxonomic approaches [5,47–50]. Used initially for species identification through match with inventories [49,51–53], DNA-based tools were later successfully employed for species delimitation and discovery [48,54–56] as well as ecology [26,57,58]. Species inference directly from sequence data has become a crucial approach [4,59–61]. The choice of method of species delimitation from molecular data has a considerable effect on estimated species entities and thus also on species richness estimates [62].

In this study, different species delimitation methods were applied to an unexplored leaf beetle fauna in a tropical montane rainforest of southern Ecuador. So far the number of species known from Ecuador is rather low due to the scarce taxonomic information available for this group. The Invertebrate Section of the Museum of Zoology at Pontifical Catholic University of Ecuador in Quito, which holds Ecuador's largest collection of native taxa, harbours ~24,200 Chrysomelidae specimens of which 76% have no identification at all (Clifford Keil, pers. comm.). Especially areas in southern Ecuador are strongly undersampled [63]. The only existing checklist considering Ecuador is outdated [64]. Since its publication, numerous species have been newly described from Ecuador in occasional papers [65–71], but only for Cassidinae a more comprehensive taxonomic treatment was published [35]. As with most of the arthropods' groups, all current taxonomic and biogeographic information, if existing at all, is widely scattered. Ecological data for species of this region are very limited [72].

Based on species estimates from a set of different delimitation methods we try to infer species diversity and compare it to estimates from parataxonomic morphological species sorting. We test the performance of several delimitation methods and evaluate how much these different yields affect estimates of potential species richness. The study area in the tropical Andes of southern Ecuador is part of a mega-diverse biodiversity hotspot [73,74], where climates and habitat types change rapidly along elevational gradients resulting in a high turnover of communities [75]. Since tropical mountain forests exhibit even higher degrees of species diversity than tropical lowland rainforests [76,77], we expect a high species richness of Chrysomelidae.

## Materials and Methods

The Ministerio del Ambiente, Ecuador, permitted us to carry out research and access to study sites was granted by Naturaleza y Cultura Internacional (NCI).

### (a) Study area and specimen sampling

Leaf beetle sampling was conducted in November and December 2010 and between May and August 2011 in the mountain forest of Podocarpus National Park (NP) and the adjacent Reserva Biológica San Francisco (RBSF) in southern Ecuador. The area belongs to the Tropical Andes, a biodiversity hotspot [73,74], and is located at the eastern slope of the Andes. It exhibits a tropical humid climate with a bimodal pattern of precipitation [78].

**Fig 1:**
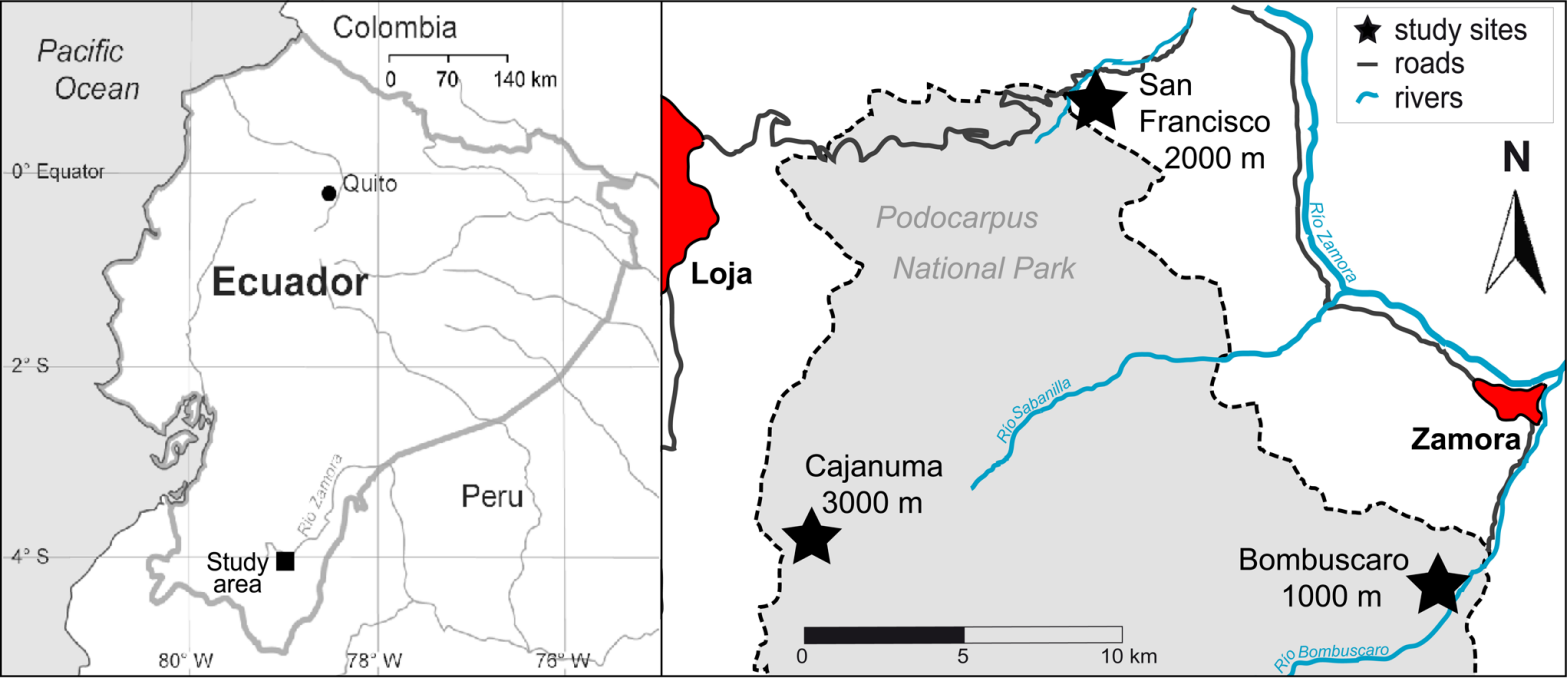
Map of Podocarpus National Park, Ecuador, with location of study sites. Cajanuma (3000 m), ECSF (2000 m), Bombuscaro (1000 m) [80].

Sampling was conducted in three different elevational zones within Podocarpus NP and RBSF, namely (1) the 1000 m zone (Bombuscaro area; 1020–1075 m a.s.l.; premontane rainforest), (2) the 2000 m zone (Estación Científica San Francisco (ECSF) area; 1913–2089 m a.s.l.; lower montane rainforest), and (3) the 3000 m zone (Cajanuma area; 2805–2891 m a.s.l.; upper montane rainforest or cloud forest) (Fig 1; classification of vegetation: [79]).

Beetles were collected by standardized sampling using three different methods, i.e. a) sweep-netting, b) beating of shrubs and smaller trees using a beating-tray and c) hand collection from the vegetation. To complete the overview of species diversity for DNA-based species delimitation, standardized sampling was complemented with additional hand collection and Malaise- as well as light-trapping collections.

Beetles were killed and collected in 70% ethanol but transferred into 96% ethanol the same day. For each sampling unit (a sampling unit is either 0.5 h sweep netting on defined plots of 20 x 20 m, 0.5 h beating on plot, 0.5 h hand collection on plot, hand collection alongside the way of one sampling day, a Malaise-trap-, or a light-tower catch) Chrysomelidae were sorted for preliminary morphospecies and one specimen of each morphospecies of each sample was used for subsequent parataxonomic sorting and molecular analysis.

### (b) DNA extraction, amplification and sequencing

Total genomic DNA was extracted from one to three legs of each specimen, using the Qiagen DNeasy^®^ Blood&Tissue Kit or Qiagen Biosprint 96BS following the manufacturers' protocol. The specimen was subsequently dry mounted and labelled to allow following morphological investigation. The 5'-end of cytochrome *c* oxidase I (658 bp, in this text referred to as COI) was amplified with the primers LCO1490 and HCO2198, or with LCO and Nancy (for primer information see S1 Table) using the Qiagen^®^ Multiplex PCR Kit. Amplification reactions were carried out in a 20 μl volume containing 10 μl QIAGEN Multiplex PCR Mastermix, 2 μl Q-Solution, 1.6 μl of each primer (both 10 pmol/μl), and 2.5 μl DNA template, and filled up to 20 μl with sterile H_2_O. The PCR temperature profile consisted of an initial denaturation at 95° (15 min), followed by 15 cycles at 94° (35 s, denaturation), 55°-40° (90 s, annealing temperature decreasing with every cycle about 1°; Touch down-PCR), 72° (90 s, extension), 25 cycles at 50° annealing temperature, and a final extension at 72° (10 min). Products were checked by electrophoresis on a 1.5% agarose gel containing GelRed^TM^ (Biotium Inc.). Successfully amplified DNA fragments were purified using Illustra^TM^ ExoStar (GE Healthcare) following the manufacturers' protocol.

PCR products were sequenced in both directions with Macrogen Europe (Amsterdam, Netherlands; http://www.macrogen.com) using the same primers. Sequences are submitted to GenBank (accession numbers KJ677272–KJ677945) and voucher specimens will be deposited in the collections of the Zoological Research Museum Alexander Koenig, Bonn (ZFMK), and the Universidad Técnica Particular de Loja, Ecuador (see S2 Table and S3 Table).

### (c) Data analyses

Sequences were assembled and edited with Geneious version 5.4.4 (Biomatters Ltd.; http://www.geneious.com/) being subsequently aligned using the implemented MUSCLE algorithm [81] (default settings, except for the maximum number of iterations (maxiters) set to 500). A Maximum-Likelihood (ML) Tree was generated in RAxML version 7.3.2 [82] using a GTR+I+Г model and 5000 bootstrap replicates. Three species of weevils (Coleoptera: Curculionidae) from GenBank and BOLD were chosen as outgroup taxa to root the tree (*Anthonomus eugenii*, *Dichromacalles dromedarius*, and *Acalles camelus*; S2 Table). They were not included in the further analyses. Branch lengths were made ultrametric with PATHd8 software [83] using relative ages of nodes and setting the root to an arbitrary age of 1.

#### Species delimitation

Since species numbers of the separate samples are crucial for the total species diversity estimate, we compared different methods of species delimitation, and these were then compared with results obtained from morphospecies. Results of the different molecular species delimitation methods (networks, distance-, GMYC- and PTP-clusters) are summed up in the term molecular operational taxonomic units (MOTUs).

In detail, the species delimitation methods comprised the following approaches: **A) Statistical parsimony analysis** [84,85] as implemented in TCS v.1.21 [86] (95% connection limit) was used to group sequences into separate haplotype networks. These networks consist of closely related haplotypes connected by mutational paths free from homoplasy with a probability of 95% [85,87]. TCS networks have been shown in various studies to correspond reliably to species across a broad range of taxa [4,54,85,88–90]. In this study all entities delimited by the TCS-analysis are called networks, even though they may contain only one haplotype or haplotypes that are connected linearly and not necessarily by loops. **B) Distance-based clustering**, which is, despite wide criticism [91,92], widely used as it is fast and easy to apply [59,93]. We used SpeciesIdentifier v.1.7.7-dev3 [92] from the TaxonDNA package (http://taxondna.sourceforge.net/) to generate clusters of sequences based on pairwise uncorrected distances at user-defined thresholds. All individuals that in direct comparison have distances below this threshold are grouped into a cluster [92]. We tested different threshold values of 3%, 5%, and 7.5%. As optimal thresholds could not be unambiguously estimated with our data set (S1 Fig), only the results of the 3%-threshold are presented. The 3%-threshold has been initially suggested in early barcoding studies by Hebert et al. [94] and is often used as standard in insect barcoding [5,47,50,95]. It was successfully used to discriminate beetle species of well-known faunas [88,96] and analyses by Papadopoulou et al. [62] confirmed this value. **C) Generalized mixed Yule-coalescent (GMYC) modelling** [4,48] as implemented in the 'splits' package (https://www.r-forge.r-project.org/projects/splits/) for the R environment (R Development Core Team, 2009) was used to estimate species boundaries directly from the phylogenetic tree [4,48] produced with COI data. This procedure exploits the differences in the rate of lineage branching at the level of species and populations, recognizable as a sudden increase of apparent diversification rate when ultrametric node height (distance to tips) is plotted against the log number of nodes in a lineage-through-time plot [97]. Its likelihood is compared then with that of the null hypothesis assuming no shift in branching rate (no separate species), and subsequently the threshold value (time) is estimated, which is the cut-off point between speciation and coalescence [11]. We use a single threshold value for our input tree [48], which has already been applied successfully to selected groups of organisms [4,48,62,88,98]. **D) Poisson tree processes (PTP) modelling** was used as implemented on the PTP web server (http://species.h-its.org/ptp/) [99]. This method is similar to GMYC-modelling but uses directly the number of substitutions instead of the time to identify branching rate transition points and therefore avoids the potentially error-prone process of making the tree ultrametric [99].

Additionally, haplotype diversity was inferred as a further independent measure for genetic diversity which demonstrated utility for exploring macroecological patterns in poorly known biota and predicting large-scale biodiversity patterns based on genetic inventories of local samples [100]. Morphological sorting of specimens into morphospecies was conducted by a taxonomic expert of the group (T.W.). It was realised only on the basis of external morphology but without the examination of genitalia and without identification literature. Ectoskeletal characters for morphospecies delimitation are shape of head, pronotum and total body, surface structures, and hairs or spines. This resembles a 'Parataxonomy' approach, which is often the only feasible method to handle the large amount of insect specimens from biodiversity studies in the tropics, where an accurate taxonomic identification is hardly possible due to the lack of modern identification keys and a potential high number of undescribed taxa [33,101,102]. Morphospecies were provided with the subfamily name and subsequently numbered.

#### Species richness estimates

For species richness estimates only sweep-netting, beating, and hand collection samples were included, as light-trapping was conducted infrequently at the 1000 m site and Malaise-trapping was not used at all. The samples from 3000 m were excluded because the area was significantly undersampled due to logistic restrictions and adverse weather conditions. Species accumulation curves were used to visualize the increase in total species diversity in relation to the number of analysed individuals and to check the completeness of our faunal survey. Method 'random' adds up the samples in a random order with 1000 iterations and calculates the mean ± 95% confidence interval, whereas method 'collector' adds up samples in the order they appear in the data. The expected total number of species was estimated using Chao2 [103], and first- and second-order Jackknife estimator using the incidence-based estimation provided by the *specpool* function of the R package vegan 2.0–5 [104]. These are widely used non-parametric estimators that use information on the rare species in an assemblage to estimate the minimum number of species in the assemblage [17] and have found to perform well in several comparative studies on species richness estimation [105,106].

For logistical reasons, more samples were obtained at 2000 m than at 1000 m. To compare mean species richness for the 1000 m and 2000 m zone at standardized levels of sampling intensity a Jackknife analysis was performed by randomly drawing 10,000 times 153 individuals (the number of individuals collected at 1000 m) from the individuals collected at 2000 m and calculating mean and 95% confidence interval of these samples. For this procedure we used the *sample* function of the R base within a simple loop.

All statistical analyses and data plots were performed in R 2.15.1 using package vegan 2.0–5 [104], Fig 5 was plotted with Microsoft Excel 2003.

## Results

### 1. General results

**Fig 2:**
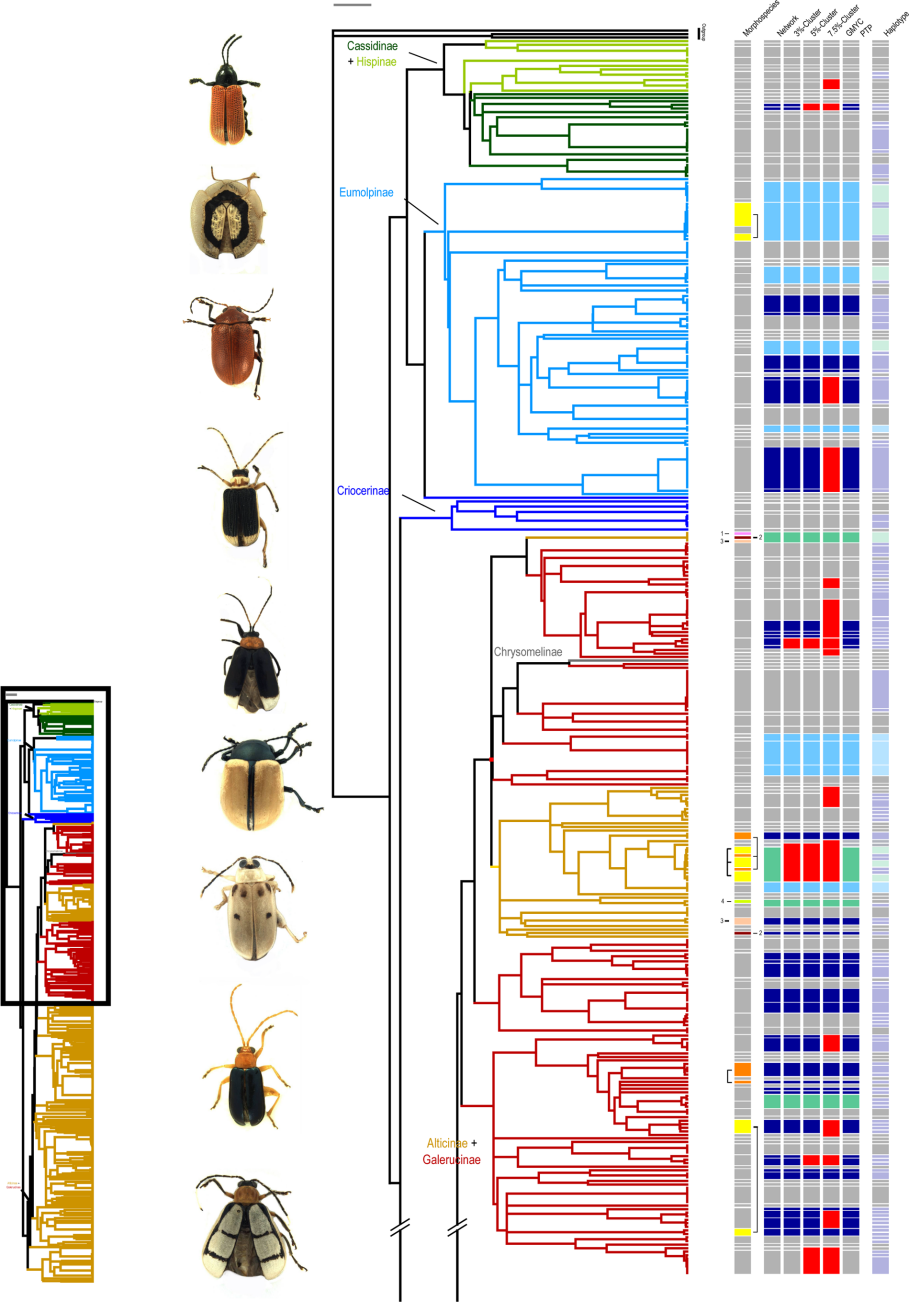
ML-Tree providing an overview about morphospecies and MOTUs and differences between the methods. Column 1: Split morphospecies are connected by brackets and share the same colour. Columns 2–7 + 8: MOTUs (Networks, 3%-, 5%-, 7.5%-, GMYC-, PTP-clusters) and haplotypes splitting a morphospecies are indicated by dark blue fields, those lumping morphospecies by light blue fields, those splitting and lumping morphospecies at the same time by green fields. Red fields indicate differences between the different molecular species delimitation methods.

**Fig 3:**
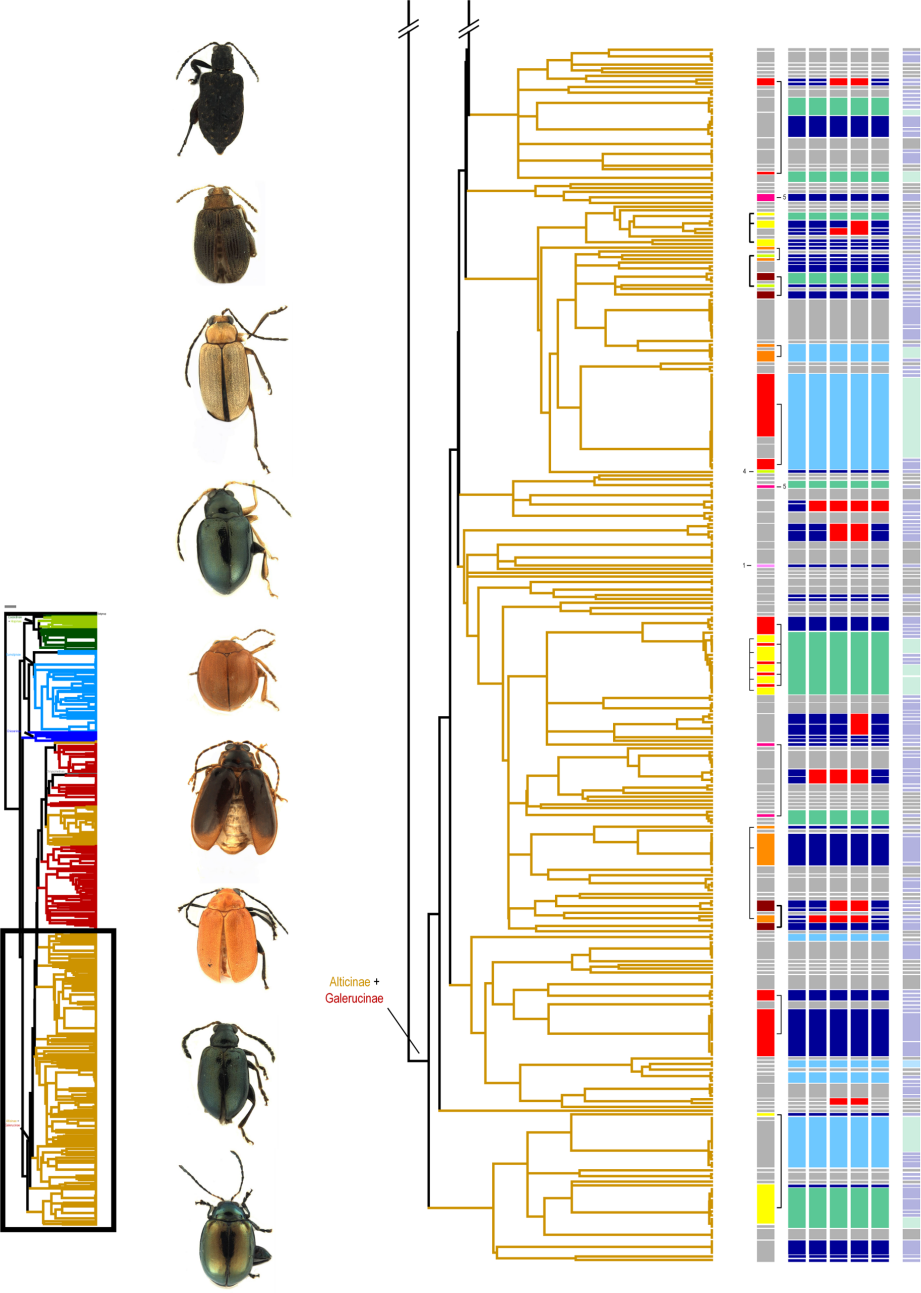
Continuation of Fig 2.

For a total of 674 specimens belonging to seven different subfamilies of Chrysomelidae COI sequences were obtained (alignment length: 658 base pairs), which were used for species delimitation: Galerucinae s.str. (represented by 163 specimens), Alticinae (371 specimens), Eumolpinae (90 specimens), Cassidinae s.str. (25 specimens), Hispinae (14 specimens), Criocerinae (10 specimens) and Chrysomelinae (1 specimen). Specimens resulted in 426 different haplotypes. Galerucinae + Alticinae (= Galerucinae s.l.), Eumolpinae, as well as Cassidinae + Hispinae (= Cassidinae s.l.) formed monophyletic clusters in the COI Maximum Likelihood tree (Figs 2 and 3 and S2 Fig), only Criocerinae appeared paraphyletic and the Chrysomelinae specimen was placed within the Galerucinae. Galerucinae s.str. and Alticinae formed several well separated clusters within Galerucinae s.l.

### 2. Species delimitation

Morphospecies sorting resulted in a total number of 266 morphospecies. The morphological sorting revealed a large amount of singletons in our data set: 140 morphospecies (52.6%) were represented by only one specimen (representing 20.8% of all analysed individuals), 47 (17.7%) by only two specimens (doubletons, 14% of all analysed individuals).

TCS-Network analyses led to a total number of 289 networks and distance-based cluster analyses to a number of 284 clusters. GMYC- and PTP-modelling resulted in a total of 288 identical GMYC- and PTP-clusters (for results of species delimitation for each specimen see S3 Table).

Despite the high congruence in species numbers, it must be noted that there were several cases of conflict between morphospecies and MOTUs (Figs 2 and 3 and S2 Fig). These contradictions arise from splitting (the individuals of one morphospecies are assigned to two or more different MOTUs) or lumping events (individuals of two or more different morphospecies are fused into one MOTU) (Table 1).

**Table 1:** Overview of splittings and lumpings.

	Morpho-species	Networks	Distance-clusters	GMYC-/PTP-clusters	Haplo-types
Species number	266	289	284	288	426
Number of Singleton specimens	140	161	156	160	324
Number of Doubleton specimens	94 (47 pairs)	104 (52 pairs)	98 (49 pairs)	102 (51 pairs)	94 (47 pairs)
Ratio MOTU number vs. morphospecies number	–	1.09	1.07	1.08	1.6
Number of perfect matches morphospecies / MOTUs	–	178	180	179	154
Number of perfect matches that are not singletons	–	62	65	63	28
Accuracy (proportion of perfect matches morphospecies / MOTUs, in respect to the total number of morphospecies)	–	66.92%	67.67%	67.29%	57.9%
Number of split morphospecies	–	42	39	41	88
Number of lumped morphospecies	–	60	61	60	42
Number of Morphospecies that were both split and lumped	–	14	14	14	18

Therefore, despite a high agreement between the number of MOTUs and the number of morphospecies (partially due to the fact that splitting and lumpings compensate one another) perfect congruence was rather low: In total we found 178 perfect matches between morphospecies and networks and 180 between morphospecies and distance-clusters, and 179 between morphospecies and GMYC-/PTP-clusters (Table 1). Splittings and lumpings were almost identical for networks, distance- and GMYC-/PTP-clusters. For all approaches, the number of morphospecies being split into several MOTUs was higher than the number of cases where several morphospecies were lumped into one MOTU. The congruence between the different molecular species delimitation methods was very high, for GMYC- and PTP-modelling results were even identical (Table 2). There were only five cases of discrepancies were one or another method was more or less restrictive than the others, and there was no case where three methods disagreed, i.e. grouped specimens in three different ways (Figs 2 and 3).

**Table 2:** Congruence between the different species delimitation methods. Shown are the numbers of perfectly matching morphospecies/MOTUs, i.e. groups that have been identically delimited by the respective methods.

	Morphospecies	Networks	Distance-clusters	GMYC-/PTP-clusters
**Morphospecies**	266	178	180	179
**Networks**	–	289	279	287
**Distance-clusters**	–	–	284	280
**GMYC/PTP-clusters**	–	–		288

Out of the 140 singleton morphospecies, 115 or 116 were also 'molecular singletons' for distance-clusters or networks and GMYC-/PTP-clusters, respectively, i.e. they were the unique representatives of a MOTU, while 126 were unique representatives of a haplotype. The remaining 25 or 24 singletons, respectively, were lumped with other specimens into one MOTU. One-hundred-and-sixty-one networks (55.7%), 156 distance-clusters (54.9%), and 160 GMYC-/PTP-clusters (55.6%) were represented by only one specimen; 324 haplotypes (76.1%) occurred only once (see Table 1).

### 3. Species richness

**Fig 4:**
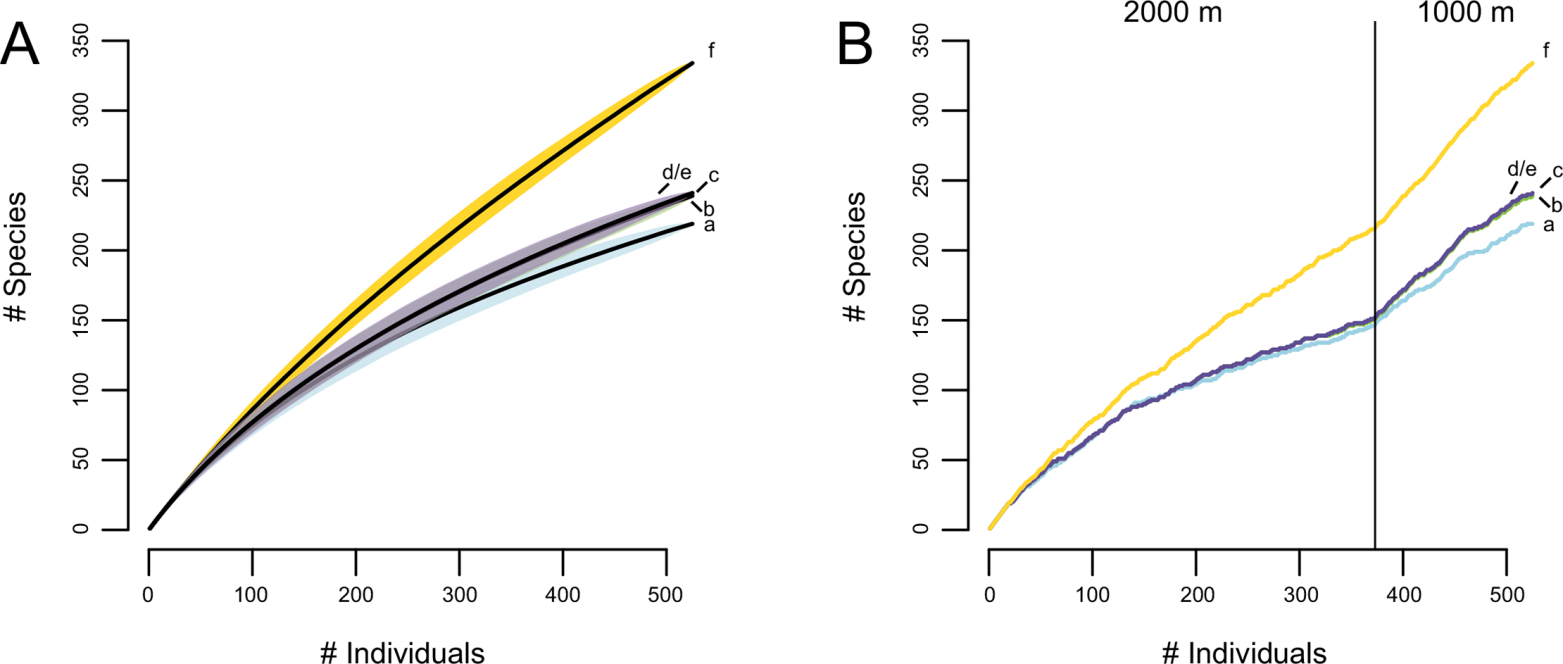
Species accumulation curves. Increase in the number of morphospecies (a), distance-clusters (b), networks (c), GMYC- and PTP-clusters (d/e) and haplotypes (f) with increasing number of analysed individuals. A: Specimens from 1000 m and 2000 m pooled. Coloured polygons indicate 95% confidence intervals. B: Specimens from 1000 m added to specimens from 2000 m.

Sweep-netting, beating, and hand collection samples of 1000 and 2000 m resulted in 525 individuals belonging to 219 morphospecies. The species accumulation curve did not reach saturation, suggesting that additional sampling would significantly increase the number of morphospecies (Fig 4A). Molecular species delimitation resulted in 241 networks and GMYC-/PTP-clusters as well as 239 distance-clusters represented by 334 haplotypes. The curves of the methods were in their slope similar to the morphospecies curve, none of them showed saturation.

The expected total number of morphospecies estimated with the Chao2 estimator was 413.6 ± 49.8 (first-order Jackknife: 338.2 ± 21.2; second-order Jackknife: 420.3) while the expected number of networks, GMYC- and PTP-clusters was 481.1 ± 56.9 (first-order Jackknife: 382 ± 24; second-order Jackknife: 480.9) and of distance-clusters 469 ± 54.9 (first-order Jackknife: 377 ± 23.7; second-order Jackknife: 473). Total number of haplotypes was estimated to be 1134.1 ± 164.1 (first-order Jackknife: 585.2 ± 35.1; second-order Jackknife: 795.5). Leaf beetle communities in the sampled areas of the Podocarpus National Park were estimated to be considerably richer by the molecular approaches than by the morphological one.

As sampling effort was different at the two elevations, the number of analysed individuals was standardized to compare species richness at the two elevational levels. At 2000 m, 372 individuals were sampled belonging to 146 morphospecies, 151 networks and GMYC-/PTP-clusters, 150 distance-clusters and 215 haplotypes. The 153 individuals from 1000 m were assigned to 90 morphospecies, 96 networks and GMYC-/PTP-clusters, 95 distance-clusters and 120 haplotypes. Standardizing the results of the 1000 m and 2000 m zone to the same number of analysed individuals (153; Jackknife) revealed no significant difference in mean morphospecies richness between 1000 m and 2000 m (Table 3). The same was valid for networks, GMYC-/PTP- and distance-clusters, as well as for haplotype numbers.

**Table 3:** Comparison of species- and haplotype richness between 1000 and 2000 m. Results standardized with Jackknife to the same number of analysed individuals (153 analysed individuals from 1000 m).

Species richness					Haplotype richness
	Morphospecies	Networks	Distance-clusters	GMYC-/PTP-clusters	Haplotypes
**1000 m**	90	96	95	96	120
**2000 m**	87.9	89.9	89.7	89.9	111.6

**Fig 5:**
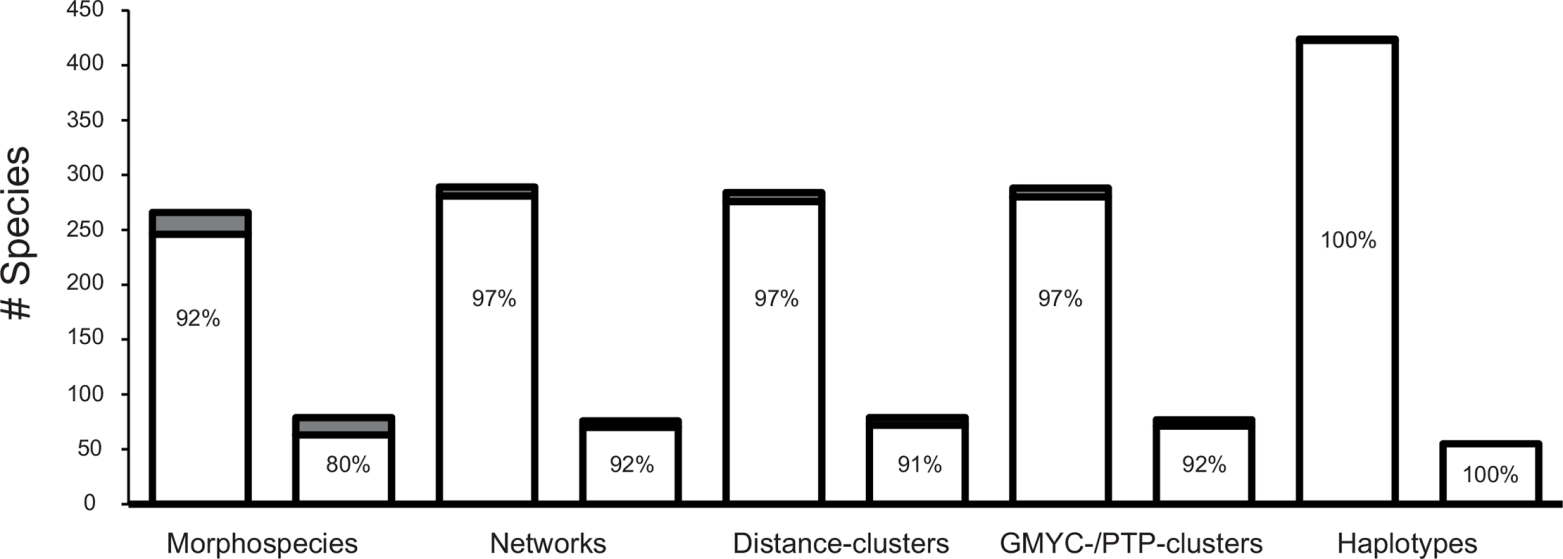
Barplots illustrating occurrence of species at elevational levels. Percentage of morphospecies, networks, distance- and GMYC-/PTP-clusters and haplotypes found at one (white) or two (grey) elevational levels. Complete dataset (left column) and dataset without singletons and doubletons (right column).

The majority of all found morphospecies occurred exclusively at a single elevational level (only 8% occurred at two elevational levels and no morphospecies was found at all three elevational levels) (Fig 5). This pattern was even more pronounced when using genetic clusters: Almost all MOTUs occurred at only one elevational level, only 3% at two levels. All haplotypes were restricted to one elevational level. As singletons and doubletons (morphospecies, MOTUs, haplotypes represented by one or two specimens) can occur only at one or two elevational levels, they were removed from the dataset for experimental reasons. The results were similar: The percentage of species found at one single elevational level was still the vast majority (80% of all morphospecies, 91% of all distance-clusters and 92% of networks and GMYC-/PTP-clusters).

The difference in species composition between the different elevations was also reflected in the species accumulation curve of specimens from 1000 and 2000 m (Fig 4B), which showed no saturation for the elevation levels under neither of the different delimitation approaches. When species from 1000 m were added to the data, the slope of the curve steeply increased.

## Discussion

This study is the first attempt to investigate the leaf beetle fauna of a tropical montane rainforest of Podocarpus NP and RBSF in southern Ecuador. Considering that mainly one stratum of vegetation was sampled (herbaceous and shrubby understorey vegetation) and that sampling is far from being complete (Fig 4), the 252 morphospecies found are a fraction of the full diversity of the area. Further sampling as well as inclusion of the canopy fauna is likely to raise species numbers considerably.

Although our survey is not complete, it provides a good insight into the characteristics of the studied leaf beetle assemblage: Initial expectations of a hyperdiverse Chrysomelidae fauna are confirmed and a high proportion of rare species with low specimen numbers was found as it is typical for tropical arthropod assemblages [107,108]. Despite the high amount of singletons (>50%) in the dataset, DNA-based species delimitations were quite robust and widely consistent with morphospecies assignments [14]. Observed and estimated species numbers were higher for DNA-based species delimitations than for morphospecies sorting. Among the DNA-based species delimitation methods, there were only slight differences in observed and estimated species numbers underscoring that in a study with geographically restricted sampling and a high percentage of singletons the choice of the exact species delimitation method is less crucial. All findings revealed by MOTUs are similar to morphospecies data, confirming the suitability of DNA taxonomy as a tool for assessing biodiversity of an unknown fauna, at least at a geographically restricted scale as in our study.

### DNA taxonomy and its implications on species richness estimates

The successful application of DNA based species delimitation to Ecuador's chrysomelid fauna is not surprising, as it has been shown to be a reliable method for identification, detection and delimitation of species for a broad variety of taxa, including beetles, in several studies (e.g. [47,62,88,96,109,110]). The different approaches were able to circumscribe distinct clusters of sequences across all subfamilies of Chrysomelidae of this study, which is an important premise if a large assemblage of unknown species is to be studied. As species numbers inferred by molecular methods were considerably higher than morphospecies numbers, DNA-based methods of species delimitation should be integrated in biodiversity assessment studies, as the morphospecies approach alone may considerably underestimate species richness [62].

We empirically compared and validated statistical parsimony analysis, distance-based clustering, and GMYC- and PTP-modelling. The high congruence among these different DNA based species delimitation methods indicates that the choice of the particular delimitation methods can be of minor relevance as for the current dataset, at least when sampling, as in this study, is geographically highly restricted (but see [111]). Probably one cause is the high amount of singletons (>50%), which does not allow too much variation for inference of the species boundary.

A geographically complete sampling of a species is usually very time and labour-intensive and often not feasible. Populations or locations are frequently isolated, either naturally or induced by the progressive fragmentation of habitats, preventing a comprehensive covering of the complete diversity. This is even more valid for tropical insects, where the desired complete inventory of a certain area is unachievable, as tropical species in general are high in numbers, but often rare and very localized [108]. While Lim et al. [9] argue that this bias may hamper semi-automated DNA-based species delimitation, the congruence of results of our different delimitation methods used seems to demonstrate the opposite. Despite a high percentage of singletons and doubletons our species richness estimates remain robust (see [14]).

Although biodiversity is usually measured in numbers of species, the entire genetic diversity of a species, including the diversity of haplotypes, is crucial for conservation. The use of haplotype diversity seems to be an even more objective measure for biodiversity as it is completely independent from species concepts or delimitation methods including their assumptions [48,100,109]. Therefore, in our analyses haplotypes are an independent estimator and a proxy for diversity in concert with DNA-based species delimitations. It has been shown that mtDNA barcode accumulation curves lead to similar results as curves generated using morphology or nuclear genetic markers [112]. Likewise, in our study the haplotype accumulation curve was similar in shape to those based on morphospecies and MOTUs and it differed only in scale. Therefore, haplotype diversity can be a valuable tool for comparing diversity at a finer scale. It also allows for the analysis of diversity of taxonomically unknown organisms, is transparent and reproducible and can be compared among sites [112]. For the current data set this did not provide dramatically new insights and probably also suffered in its utility from our limited intraspecific sampling. However, it should be expected that this method will be highly informative if applied on a wider geographical scale with much more extended infraspecific sampling [100].

### Chrysomelid diversity

This is the first study of site-specific data on leaf beetle richness for Ecuador. Species numbers are difficult to compare with leaf beetle studies from other Neotropical regions due to differences in geographical scale, taxonomic focus, sampling effort and methods (e.g. [41,44]).

Observed and estimated species numbers are higher for molecular species delimitation methods than for morphological species sorting but the differences at a local level are not too great. This might change considerably with a much wider geographical sampling where intraspecific variation is covered more extensively and interspecific divergence decreases as one encounters more closely related, allopatrically distributed species [111]. This might alter species delimitation and lead to different results in species richness.

A species accumulation curve that does not reach saturation is frequently found for samples from rainforest communities of insects [33,108]. In the present study this could be partly explained by undersampling, but might occur also because most species are rather rare (illustrated by a large proportion of singletons: 53% and 55–56% of the morphospecies and MOTUs, respectively). These rare species are an important part of rainforest communities of insect herbivores, often representing from 30% up to more than half of all species in tropical arthropod samples [33,107,108]. They may prevent the species accumulation curve from getting saturated even in very large sample series achieved with a huge sampling effort. As the number of specimens included in our study is rather small compared to many tropical arthropod surveys (see [107]), the percentage of singletons might decrease with additional sampling effort, but is expected to remain quite high.

In contrast to expectations from the literature [113] no significant difference in mean species richness resulted between the two elevational levels (1000 and 2000 m). A difference in species number could have been expected, as insect species richness often declines with increasing elevation or shows a hump-shaped distribution [113]. Results revealed that leaf beetle communities differ strongly between the elevational levels [41], an issue that should be examined in more detail in future studies. Although the high turnover of communities might be slightly exaggerated by possibly insufficient sampling, a high turnover of communities was expected because, even though the two sampling areas of the 1000 and 2000 m zone are as close as ~20 km, the collection sites exhibit remarkable differences in climate and vegetation.

Additional Malaise- and light-trapping added more specimens and species with different ecology and habitat preferences (flying and/or nocturnal species) to our sampling. However, the canopy, which is considered to be the most diverse habitat in tropical rainforests [21,114], especially for leaf-beetles [115,116,117], was not sampled here. These communities are known to be quite distinct from those of the understorey [115]. Therefore, a thorough sampling of the canopy with fogging would probably increase species numbers from our sample sites. Studies that included the rainforest canopy, or which were part of large-scale studies and inventories yielded much higher species numbers (e.g. [117], > 650 species in a Peruvian rainforest canopy). These large-scale research programmes are capable of more intense sampling over a longer time period and with more manpower compared to the present study [44,102,118]. This illustrates that accelerated biodiversity assessments with DNA-based techniques, like traditional taxonomic approaches, very much depend on the sampling, in terms of benefits of the methodological protocols [9,14], but also in terms of completeness (i.e. total species numbers).

## Conclusions

The present study provides a rapid biodiversity assessment of the hitherto unstudied leaf beetle fauna of the understorey vegetation of a tropical montane rainforest in Ecuador. It revealed a remarkable diversity of Chrysomelidae and is a good starting point for future, more detailed research on this fauna, also regarding a thorough taxonomic re-examination and the formal description with binary Linnaean names of possibly new species. The large species turnover found between the different elevations suggests the need for further investigation of differences between the communities, particularly along the elevational gradient. This seems even more important since the rates of forest conversion of the Andean mountain forests are high, with Ecuador suffering the highest annual deforestation rate in South-America (-1.9%) [119,120].

Whereas the integration of different DNA-based approaches for estimating species richness is strongly recommended [8], the choice of the molecular species delimitation method, at least with our data, seems to be of minor relevance. All our results illustrate the high potential of DNA-based species delimitation for exploring communities of hyperdiverse taxa even before they are taxonomically identified and formally described [4]. It is a useful complement to morphological approaches due to its repeatability (iterative taxonomy; [121]) and capability of revealing cryptic diversity as an effective tool for taxonomic species delimitation and description. This is an important requirement for a universal tool for direct biodiversity measurement.

## Supporting Information

S1 Fig Calibration of distance clusters with morphospecies to determine best threshold. (EPS)

S2 Fig ML-Tree providing an overwiew about morphospecies and MOTUs and differences between the methods. Column 1: Split morphospecies are connected by brackets and share the same colour. Columns 2–7 + 8: MOTUs (Networks, 3%-, 5%-, 7.5%-, GMYC-, PTP-clusters) and haplotypes splitting a morphospecies are indicated by dark blue fields, those lumping morphospecies by light blue fields, those splitting and lumping morphospecies at the same time by green fields. Red fields indicate differences between the different molecular species delimitation methods.
(TIF)

S1 Table Primer information. (PDF)

S2 Table Specimen list with sampling information. Podocarpus NP = Podocarpus National Park, RBSF = Reserva Biológica San Francisco.
(PDF)

S3 Table Results of species delimitation for each specimen. (PDF)
