# Supplementary figures and images for: Exploring the Leaf Beetle Fauna (Coleoptera: Chrysomelidae) of an Ecuadorian Mountain Forest Using DNA Barcoding
Source: PLoS One. 2016 Feb 5;11(2):e0148268. doi: 10.1371/journal.pone.0148268 (PMC4744027; doi:10.1371/journal.pone.0148268)

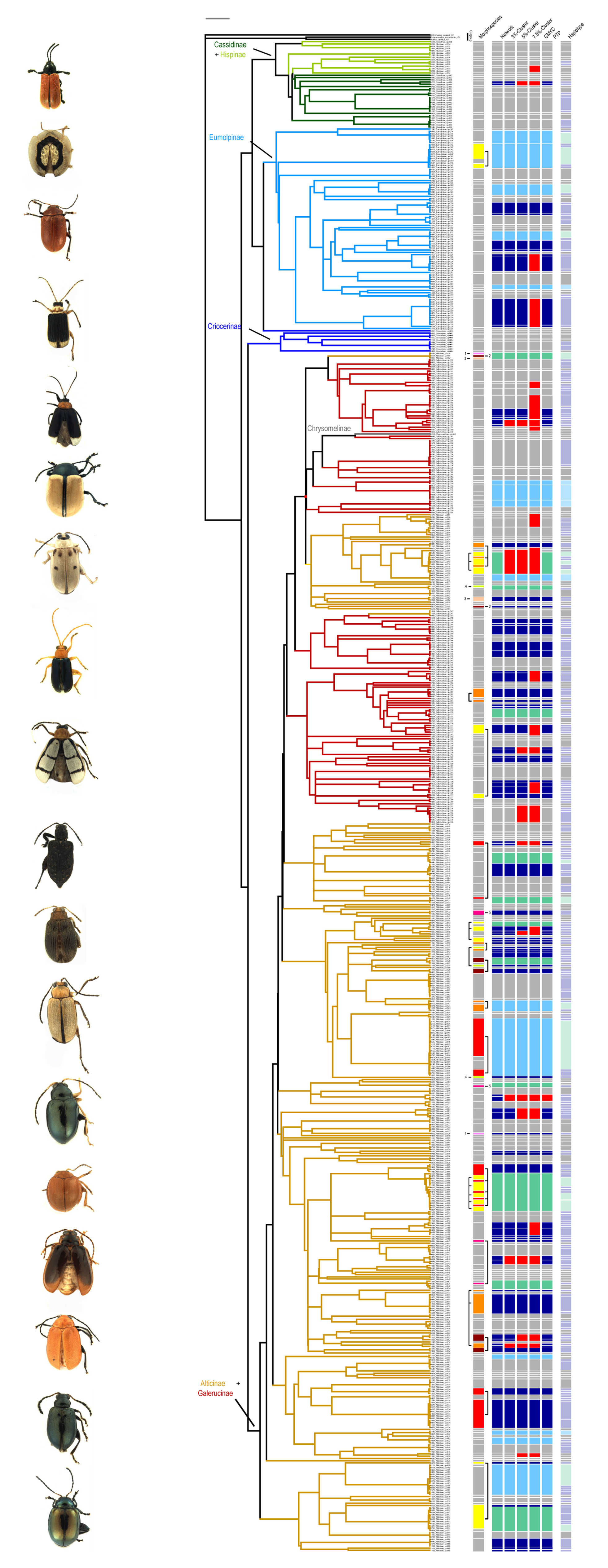

Supplement: S2 Fig — Column 1: Split morphospecies are connected by brackets and share the same colour. Columns 2–7 + 8: MOTUs (Networks, 3%-, 5%-, 7.5%-, GMYC-, PTP-clusters) and haplotypes splitting a morphospecies are indicated by dark blue fields, those lumping morphospecies by light blue fields, those splitting and lumping morphospecies at the same time by green fields. Red fields indicate differences between the different molecular species delimitation methods. (TIF) [file pone.0148268.s002.tif]
