# Supplementary material for: Exploring the Leaf Beetle Fauna (Coleoptera: Chrysomelidae) of an Ecuadorian Mountain Forest Using DNA Barcoding
Source: PLoS One. 2016 Feb 5;11(2):e0148268. doi: 10.1371/journal.pone.0148268 (PMC4744027; doi:10.1371/journal.pone.0148268)
Supplement: S1 Table — (PDF) [file pone.0148268.s003.pdf]

Table S1. Primer information.

Name	Sequence	Direction	Reference
LCO1490	5´- GGT CAA CAA ATC ATA AAG ATA TTG G -3´	forward	Folmer O et al. (1994) DNA primers for amplification of mitochondrial cytochrome c oxidase subunit I from diverse metazoan invertebrates. Mol Mar Biol Biotech 3
HCO 2198	5´- TAA ACT TCA GGG TGA CCA AAA AAT CA -3´	reverse	Folmer O et al. (1994) DNA primers for amplification of mitochondrial cytochrome c oxidase subunit I from diverse metazoan invertebrates. Mol Mar Biol Biotech 3
Nancy	5´- CCC GGT AAA ATT AAA ATA TAA ACT TC -3´	reverse	Simon C et al. (1994) Evolution, weighting and phylogenetic utility of mitochondrial gene sequences and a compilation of conserved polymerase chain reaction primers. Ann Entomol Soc Am 87: 651-701.
