# Supplementary material for: Exploring the Leaf Beetle Fauna (Coleoptera: Chrysomelidae) of an Ecuadorian Mountain Forest Using DNA Barcoding
Source: PLoS One. 2016 Feb 5;11(2):e0148268. doi: 10.1371/journal.pone.0148268 (PMC4744027; doi:10.1371/journal.pone.0148268)
Supplement: S2 Table — Podocarpus NP = Podocarpus National Park, RBSF = Reserva Biológica San Francisco. (PDF) [file pone.0148268.s004.pdf]

Table S2. Specimen list with sampling information. Podocarpus NP = Podocarpus National Park, RBSF = Reserva Biológica San Francisco.

Specimen	GenBank Accession Nr.	Location	Sampling Area	Latitude (UTM,17S)	Longitude (UTM,17S)	Altitude (m a.s.l.)	Sampling Date	Sampling Method
BT_0001_Eumolpinae sp. 1	KJ677921	Podocarpus NP/RBSF, Ecuador	ECSF	9560781.7	713299.6	1826	Nov/Dec 2010	Hand-Collection
BT_0002_Alticinae sp. 42	KJ677411	Podocarpus NP/RBSF, Ecuador	ECSF	9560781.7	713299.6	1826	Nov/Dec 2010	Hand-Collection
BT_0004_Eumolpinae sp. 42	KJ677862	Podocarpus NP/RBSF, Ecuador	ECSF	9560781.7	713299.6	1826	Nov/Dec 2010	Hand-Collection
BT_0005_Galerucinae sp. 40	KJ677774	Podocarpus NP/RBSF, Ecuador	ECSF	9560781.7	713299.6	1826	Nov/Dec 2010	Hand-Collection
BT_0007_Galerucinae sp. 38	KJ677526	Podocarpus NP/RBSF, Ecuador	ECSF	9560781.7	713299.6	1826	Nov/Dec 2010	Hand-Collection
BT_0008_Alticinae sp. 243	KJ677417	Podocarpus NP/RBSF, Ecuador	ECSF	9560781.7	713299.6	1826	Nov/Dec 2010	Hand-Collection
BT_0012_Eumolpinae sp. 21	KJ677897	Podocarpus NP/RBSF, Ecuador	ECSF	9560781.7	713299.6	1826	Nov/Dec 2010	Hand-Collection
BT_0015_Galerucinae sp. 76	KJ677559	Podocarpus NP/RBSF, Ecuador	ECSF	9560781.7	713299.6	1826	Nov/Dec 2010	Hand-Collection
BT_0017_Alticinae sp. 43	KJ677407	Podocarpus NP/RBSF, Ecuador	ECSF	9560781.7	713299.6	1826	Nov/Dec 2010	Hand-Collection
BT_0021_Alticinae sp. 7	KJ677705	Podocarpus NP/RBSF, Ecuador	ECSF	9560781.7	713299.6	1826	Nov/Dec 2010	Hand-Collection
BT_0022_Alticinae sp. 219	KJ677711	Podocarpus NP/RBSF, Ecuador	ECSF	9560781.7	713299.6	1826	Nov/Dec 2010	Hand-Collection
BT_0024_Galerucinae sp. 1	KJ677550	Podocarpus NP/RBSF, Ecuador	ECSF	9560781.7	713299.6	1826	Nov/Dec 2010	Hand-Collection
BT_0033_Galerucinae sp. 37	KJ677555	Podocarpus NP/RBSF, Ecuador	ECSF	9560781.7	713299.6	1826	Nov/Dec 2010	Hand-Collection
BT_0034_Eumolpinae sp. 14	KJ677931	Podocarpus NP/RBSF, Ecuador	ECSF	9560781.7	713299.6	1826	Nov/Dec 2010	Hand-Collection
BT_0035_Eumolpinae sp. 6	KJ677907	Podocarpus NP/RBSF, Ecuador	ECSF	9560781.7	713299.6	1826	Nov/Dec 2010	Hand-Collection
BT_0036_Galerucinae sp. 11	KJ677532	Podocarpus NP/RBSF, Ecuador	ECSF	9560781.7	713299.6	1826	Nov/Dec 2010	Hand-Collection
BT_0043_Galerucinae sp. 5	KJ677545	Podocarpus NP/RBSF, Ecuador	ECSF	9560781.7	713299.6	1826	Nov/Dec 2010	Hand-Collection
BT_0046_Alticinae sp. 243	KJ677415	Podocarpus NP/RBSF, Ecuador	ECSF	9560781.7	713299.6	1826	Nov/Dec 2010	Hand-Collection
BT_0047_Alticinae sp. 42	KJ677412	Podocarpus NP/RBSF, Ecuador	ECSF	9560781.7	713299.6	1826	Nov/Dec 2010	Hand-Collection
BT_0048_Galerucinae sp. 39	KJ677523	Podocarpus NP/RBSF, Ecuador	ECSF	9560781.7	713299.6	1826	Nov/Dec 2010	Hand-Collection
BT_0049_Galerucinae sp. 41	KJ677775	Podocarpus NP/RBSF, Ecuador	ECSF	9560781.7	713299.6	1826	Nov/Dec 2010	Hand-Collection
BT_0088_Galerucinae sp. 7	KJ677543	Podocarpus NP/RBSF, Ecuador	ECSF	9560781.7	713299.6	1826	20.11.2010	Light trapping
BT_0089_Eumolpinae sp. 1	KJ677922	Podocarpus NP/RBSF, Ecuador	ECSF	9560781.7	713299.6	1826	20.11.2010	Light trapping
BT_0090_Galerucinae sp. 76	KJ677558	Podocarpus NP/RBSF, Ecuador	ECSF	9560781.7	713299.6	1826	21.11.2010	Light trapping
BT_0091_Eumolpinae sp. 1	KJ677923	Podocarpus NP/RBSF, Ecuador	ECSF	9560781.7	713299.6	1826	21.11.2010	Light trapping
BT_0094_Galerucinae sp. 11	KJ677533	Podocarpus NP/RBSF, Ecuador	ECSF	9560455	713721	2002	22.11.2010	Hand-Collection
BT_0095_Cassidinae sp. 1	KJ677873	Podocarpus NP/RBSF, Ecuador	ECSF	9560352	713911	2011	22.11.2010	Hand-Collection
BT_0096_Alticinae sp. 10	KJ677729	Podocarpus NP/RBSF, Ecuador	ECSF	9560352	713911	2011	22.11.2010	Hand-Collection
BT_0098_Galerucinae sp. 2	KJ677547	Podocarpus NP/RBSF, Ecuador	ECSF	9560352	713911	2011	22.11.2010	Hand-Collection
BT_0099_Galerucinae sp. 7	KJ677512	Podocarpus NP/RBSF, Ecuador	ECSF	9560352	713911	2011	22.11.2010	Hand-Collection
BT_0102_Alticinae sp. 44	KJ677374	Podocarpus NP/RBSF, Ecuador	ECSF	9560352	713911	2011	22.11.2010	Hand-Collection
BT_0103_Eumolpinae sp. 38	KJ677927	Podocarpus NP/RBSF, Ecuador	ECSF	9560352	713911	2011	22.11.2010	Hand-Collection
BT_0107_Galerucinae sp. 46	KJ677632	Podocarpus NP/RBSF, Ecuador	ECSF	9560352	713911	2011	22.11.2010	Hand-Collection
BT_0109_Alticinae sp. 251	KJ677459	Podocarpus NP/RBSF, Ecuador	ECSF	9560352	713911	2011	22.11.2010	Hand-Collection
BT_0110_Alticinae sp. 87	KJ677497	Podocarpus NP/RBSF, Ecuador	ECSF	9560352	713911	2011	22.11.2010	Hand-Collection
BT_0114_Galerucinae sp. 62	KJ677756	Podocarpus NP/RBSF, Ecuador	ECSF	9560352	713911	2011	22.11.2010	Hand-Collection
BT_0115_Alticinae sp. 98	KJ677286	Podocarpus NP/RBSF, Ecuador	ECSF	9560352	713911	2011	22.11.2010	Hand-Collection
BT_0118_Eumolpinae sp. 19	KJ677877	Podocarpus NP/RBSF, Ecuador	ECSF	9560352	713911	2011	22.11.2010	Hand-Collection
BT_0119_Alticinae sp. 124	KJ677494	Podocarpus NP/RBSF, Ecuador	ECSF	9560352	713911	2011	22.11.2010	Hand-Collection
BT_0121_Alticinae sp. 107	KJ677776	Podocarpus NP/RBSF, Ecuador	ECSF	9560352	713911	2011	22.11.2010	Hand-Collection
BT_0123_Alticinae sp. 129	KJ677769	Podocarpus NP/RBSF, Ecuador	ECSF	9560352	713911	2011	22.11.2010	Hand-Collection
BT_0125_Alticinae sp. 97	KJ677311	Podocarpus NP/RBSF, Ecuador	ECSF	9560352	713911	2011	22.11.2010	Hand-Collection
BT_0126_Alticinae sp. 123	KJ677618	Podocarpus NP/RBSF, Ecuador	ECSF	9560352	713911	2011	22.11.2010	Hand-Collection
BT_0130_Galerucinae sp. 34	KJ677691	Podocarpus NP/RBSF, Ecuador	ECSF	9560352	713911	2011	22.11.2010	Hand-Collection
BT_0134_Galerucinae sp. 7	KJ677513	Podocarpus NP/RBSF, Ecuador	ECSF	9560423	714258	1933	24.11.2010	Hand-Collection
BT_0135_Eumolpinae sp. 19	KJ677878	Podocarpus NP/RBSF, Ecuador	ECSF	9560352	713911	2011	24.11.2010	Hand-Collection
BT_0137_Cassidinae sp. 4	KJ677850	Podocarpus NP/RBSF, Ecuador	ECSF	9560352	713911	2011	24.11.2010	Hand-Collection
BT_0139_Alticinae sp. 10	KJ677730	Podocarpus NP/RBSF, Ecuador	ECSF	9560352	713911	2011	24.11.2010	Hand-Collection
BT_0140_Alticinae sp. 28	KJ677346	Podocarpus NP/RBSF, Ecuador	ECSF	9560352	713911	2011	24.11.2010	Hand-Collection
BT_0144_Eumolpinae sp. 38	KJ677926	Podocarpus NP/RBSF, Ecuador	ECSF	9560352	713911	2011	24.11.2010	Hand-Collection
BT_0145_Galerucinae sp. 61	KJ677514	Podocarpus NP/RBSF, Ecuador	ECSF	9560352	713911	2011	24.11.2010	Hand-Collection
BT_0146_Alticinae sp. 29	KJ677442	Podocarpus NP/RBSF, Ecuador	ECSF	9560352	713911	2011	24.11.2010	Hand-Collection
BT_0147_Alticinae sp. 62	KJ677421	Podocarpus NP/RBSF, Ecuador	ECSF	9560352	713911	2011	24.11.2010	Hand-Collection
BT_0148_Alticinae sp. 66	KJ677468	Podocarpus NP/RBSF, Ecuador	ECSF	9560352	713911	2011	24.11.2010	Hand-Collection
BT_0149_Alticinae sp. 249	KJ677456	Podocarpus NP/RBSF, Ecuador	ECSF	9560352	713911	2011	24.11.2010	Hand-Collection
BT_0153_Alticinae sp. 109	KJ677669	Podocarpus NP/RBSF, Ecuador	ECSF	9560352	713911	2011	24.11.2010	Hand-Collection
BT_0154_Alticinae sp. 115	KJ677287	Podocarpus NP/RBSF, Ecuador	ECSF	9560352	713911	2011	24.11.2010	Hand-Collection
BT_0155_Alticinae sp. 193	KJ677671	Podocarpus NP/RBSF, Ecuador	ECSF	9560352	713911	2011	24.11.2010	Hand-Collection
BT_0157_Alticinae sp. 97	KJ677300	Podocarpus NP/RBSF, Ecuador	ECSF	9560352	713911	2011	24.11.2010	Hand-Collection
BT_0158_Eumolpinae sp. 2	KJ677932	Podocarpus NP/RBSF, Ecuador	ECSF	9560352	713911	2011	24.11.2010	Hand-Collection
BT_0159_Galerucinae sp. 96	KJ677683	Podocarpus NP/RBSF, Ecuador	ECSF	9560352	713911	2011	24.11.2010	Hand-Collection
BT_0174_Galerucinae sp. 46	KJ677633	Podocarpus NP/RBSF, Ecuador	ECSF	9560352	713911	2011	24.11.2010	Hand-Collection
BT_0176_Galerucinae sp. 46	KJ677636	Podocarpus NP/RBSF, Ecuador	ECSF	9560352	713911	2011	24.11.2010	Hand-Collection
BT_0183_Galerucinae sp. 34	KJ677692	Podocarpus NP/RBSF, Ecuador	ECSF	9560352	713911	2011	24.11.2010	Hand-Collection
BT_0188_Galerucinae sp. 11	KJ677535	Podocarpus NP/RBSF, Ecuador	ECSF	9560781.7	713299.6	1826	24.11.2010	Light trapping
BT_0189_Alticinae sp.161	KJ677361	Podocarpus NP/RBSF, Ecuador	ECSF	9560781.7	713299.6	1826	24.11.2010	Light trapping
BT_0190_Eumolpinae sp. 1	KJ677924	Podocarpus NP/RBSF, Ecuador	ECSF	9560781.7	713299.6	1826	24.11.2010	Light trapping
BT_0196_Galerucinae sp. 10	KJ677807	Podocarpus NP/RBSF, Ecuador	Bombuscaro	9544506	725125	1104	25.11.2010	Hand-Collection
BT_0199_Alticinae sp. 118	KJ677667	Podocarpus NP/RBSF, Ecuador	ECSF	9560352	713911	2011	27.11.2010	Hand-Collection
BT_0201_Chrysomelinae sp. 2	KJ677759	Podocarpus NP/RBSF, Ecuador	Bombuscaro	9544734	725457	1046	29.11.2010	Hand-Collection
BT_0202_Galerucinae sp. 32	KJ677273	Podocarpus NP/RBSF, Ecuador	Bombuscaro	9544506	725125	1104	29.11.2010	Hand-Collection
BT_0204_Hispinae sp. 2	KJ677856	Podocarpus NP/RBSF, Ecuador	Bombuscaro	9544506	725125	1104	29.11.2010	Hand-Collection
BT_0207_Galerucinae sp. 69	KJ677778	Podocarpus NP/RBSF, Ecuador	ECSF	9560352	713911	2011	01.12.2010	Hand-Collection
BT_0208_Eumolpinae sp. 19	KJ677879	Podocarpus NP/RBSF, Ecuador	ECSF	9560307	713655	1993	02.12.2010	Hand-Collection
BT_0209_Cassidinae sp. 5	KJ677822	Podocarpus NP/RBSF, Ecuador	ECSF	9560352	713911	2011	02.12.2010	Hand-Collection
BT_0211_Alticinae sp. 87	KJ677495	Podocarpus NP/RBSF, Ecuador	ECSF	9560352	713911	2011	02.12.2010	Hand-Collection
BT_0212_Galerucinae sp. 66	KJ677794	Podocarpus NP/RBSF, Ecuador	ECSF	9560352	713911	2011	02.12.2010	Hand-Collection
BT_0213_Galerucinae sp. 24	KJ677733	Podocarpus NP/RBSF, Ecuador	ECSF	9560352	713911	2011	02.12.2010	Hand-Collection
BT_0214_Alticinae sp. 28	KJ677349	Podocarpus NP/RBSF, Ecuador	ECSF	9560352	713911	2011	02.12.2010	Hand-Collection
BT_0218_Galerucinae sp. 31	KJ677751	Podocarpus NP/RBSF, Ecuador	ECSF	9560352	713911	2011	02.12.2010	Hand-Collection
BT_0219_Eumolpinae sp. 73	KJ677831	Podocarpus NP/RBSF, Ecuador	ECSF	9560352	713911	2011	02.12.2010	Hand-Collection
BT_0220_Alticinae sp. 115	KJ677288	Podocarpus NP/RBSF, Ecuador	ECSF	9560352	713911	2011	02.12.2010	Hand-Collection

BT_0221_Eumolpinae sp. 10	KJ677906	Podocarpus NP/RBSF, Ecuador	ECSF	9560352	713911	2011	02.12.2010	Hand-Collection
BT_0223_Galerucinae sp. 34	KJ677689	Podocarpus NP/RBSF, Ecuador	ECSF	9560352	713911	2011	02.12.2010	Hand-Collection
BT_0227_Cassidinae sp. 14	KJ677874	Podocarpus NP/RBSF, Ecuador	ECSF	9560352	713911	2011	02.12.2010	Hand-Collection
BT_0228_Cassidinae sp. 7	KJ677837	Podocarpus NP/RBSF, Ecuador	ECSF	9560352	713911	2011	02.12.2010	Hand-Collection
BT_0231_Hispinae sp. 3	KJ677842	Podocarpus NP/RBSF, Ecuador	ECSF	9560352	713911	2011	02.12.2010	Hand-Collection
BT_0232_Hispinae sp. 4	KJ677272	Podocarpus NP/RBSF, Ecuador	ECSF	9560352	713911	2011	02.12.2010	Hand-Collection
BT_0233_Alticinae sp. 61	KJ677282	Podocarpus NP/RBSF, Ecuador	ECSF	9560352	713911	2011	02.12.2010	Hand-Collection
BT_0234_Alticinae sp. 97	KJ677308	Podocarpus NP/RBSF, Ecuador	ECSF	9560352	713911	2011	02.12.2010	Hand-Collection
BT_0235_Alticinae sp. 10	KJ677728	Podocarpus NP/RBSF, Ecuador	ECSF	9560352	713911	2011	02.12.2010	Hand-Collection
BT_0236_Alticinae sp. 156	KJ677727	Podocarpus NP/RBSF, Ecuador	ECSF	9560352	713911	2011	02.12.2010	Hand-Collection
BT_0239_Galerucinae sp. 2	KJ677548	Podocarpus NP/RBSF, Ecuador	ECSF	9560352	713911	2011	03.12.2010	Hand-Collection
BT_0240_Eumolpinae sp. 21	KJ677898	Podocarpus NP/RBSF, Ecuador	ECSF	9560352	713911	2011	03.12.2010	Hand-Collection
BT_0243_Alticinae sp. 118	KJ677666	Podocarpus NP/RBSF, Ecuador	ECSF	9560352	713911	2011	03.12.2010	Hand-Collection
BT_0244_Alticinae sp. 97	KJ677309	Podocarpus NP/RBSF, Ecuador	ECSF	9560352	713911	2011	03.12.2010	Hand-Collection
BT_0245_Galerucinae sp. 34	KJ677693	Podocarpus NP/RBSF, Ecuador	ECSF	9560352	713911	2011	03.12.2010	Hand-Collection
BT_0246_Galerucinae sp. 30	KJ677701	Podocarpus NP/RBSF, Ecuador	ECSF	9560470	713965	1990	03.12.2010	Hand-Collection
BT_0247_Criocerinae sp. 1	KJ677813	Podocarpus NP/RBSF, Ecuador	ECSF	9560352	713911	2011	05.12.2010	Hand-Collection
BT_0249_Criocerinae sp. 1	KJ677814	Podocarpus NP/RBSF, Ecuador	ECSF	9560352	713911	2011	05.12.2010	Hand-Collection
BT_0252_Galerucinae sp. 34	KJ677694	Podocarpus NP/RBSF, Ecuador	ECSF	9560352	713911	2011	05.12.2010	Hand-Collection
BT_0254_Eumolpinae sp. 23	KJ677899	Podocarpus NP/RBSF, Ecuador	ECSF	9560352	713911	2011	05.12.2010	Hand-Collection
BT_0256_Alticinae sp. 31	KJ677625	Podocarpus NP/RBSF, Ecuador	ECSF	9560781.7	713299.6	1826	03.12.2010	Light trapping
BT_0257_Galerucinae sp. 1	KJ677551	Podocarpus NP/RBSF, Ecuador	ECSF	9560781.7	713299.6	1826	03.12.2010	Light trapping
BT_0258_Galerucinae sp. 5	KJ677546	Podocarpus NP/RBSF, Ecuador	ECSF	9560781.7	713299.6	1826	03.12.2010	Light trapping
BT_0259_Alticinae sp. 96	KJ677469	Podocarpus NP/RBSF, Ecuador	ECSF	9560352	713911	2011	08.12.2010	Hand-Collection
BT_0267_Alticinae sp. 29	KJ677443	Podocarpus NP/RBSF, Ecuador	ECSF	9560580	713650	1900	08.12.2010	Malaise Trapping
BT_0268_Alticinae sp. 158	KJ677582	Podocarpus NP/RBSF, Ecuador	ECSF	9560580	713650	1900	08.12.2010	Malaise Trapping
BT_0269_Alticinae sp. 86	KJ677396	Podocarpus NP/RBSF, Ecuador	ECSF	9560580	713650	1900	08.12.2010	Malaise Trapping
BT_0271_Alticinae sp. 64	KJ677447	Podocarpus NP/RBSF, Ecuador	ECSF	9560580	713650	1900	08.12.2010	Malaise Trapping
BT_0273_Alticinae sp. 141	KJ677585	Podocarpus NP/RBSF, Ecuador	ECSF	9560580	713650	1900	08.12.2010	Malaise Trapping
BT_0276_Alticinae sp. 122	KJ677777	Podocarpus NP/RBSF, Ecuador	ECSF	9560580	713650	1900	08.12.2010	Malaise Trapping
BT_0278_Alticinae sp. 124	KJ677491	Podocarpus NP/RBSF, Ecuador	ECSF	9560580	713650	1900	08.12.2010	Malaise Trapping
BT_0279_Alticinae sp. 115	KJ677289	Podocarpus NP/RBSF, Ecuador	ECSF	9560580	713650	1900	08.12.2010	Malaise Trapping
BT_0283_Eumolpinae sp. 20	KJ677941	Podocarpus NP/RBSF, Ecuador	ECSF	9560580	713650	1900	08.12.2010	Malaise Trapping
BT_0284_Alticinae sp. 87	KJ677496	Podocarpus NP/RBSF, Ecuador	ECSF	9560580	713650	1900	08.12.2010	Malaise Trapping
BT_0285_Galerucinae sp. 22	KJ677556	Podocarpus NP/RBSF, Ecuador	ECSF	9560580	713650	1900	08.12.2010	Malaise Trapping
BT_0288_Eumolpinae sp. 17	KJ677909	Podocarpus NP/RBSF, Ecuador	ECSF	9560781.7	713299.6	1826	12.12.2010	Light trapping
BT_0289_Eumolpinae sp. 1	KJ677925	Podocarpus NP/RBSF, Ecuador	ECSF	9560781.7	713299.6	1826	12.12.2010	Light trapping
BT_0292_Galerucinae sp. 76	KJ677557	Podocarpus NP/RBSF, Ecuador	ECSF	9560781.7	713299.6	1826	12.12.2010	Light trapping
BT_0295_Galerucinae sp. 31	KJ677752	Podocarpus NP/RBSF, Ecuador	ECSF	9560352	713911	2011	12.12.2010	Hand-Collection
BT_0296_Alticinae sp. 5	KJ677652	Podocarpus NP/RBSF, Ecuador	ECSF	9560580	713650	1900	12.12.2010	Malaise Trapping
BT_0297_Alticinae sp. 142	KJ677593	Podocarpus NP/RBSF, Ecuador	ECSF	9560580	713650	1900	12.12.2010	Malaise Trapping
BT_0298_Alticinae sp. 63	KJ677342	Podocarpus NP/RBSF, Ecuador	ECSF	9560580	713650	1900	12.12.2010	Malaise Trapping
BT_0301_Alticinae sp. 83	KJ677334	Podocarpus NP/RBSF, Ecuador	ECSF	9560580	713650	1900	12.12.2010	Malaise Trapping
BT_0302_Alticinae sp. 86	KJ677398	Podocarpus NP/RBSF, Ecuador	ECSF	9560580	713650	1900	12.12.2010	Malaise Trapping
BT_0305_Alticinae sp. 81	KJ677765	Podocarpus NP/RBSF, Ecuador	ECSF	9560580	713650	1900	12.12.2010	Malaise Trapping
BT_0307_Alticinae sp. 115	KJ677297	Podocarpus NP/RBSF, Ecuador	ECSF	9560580	713650	1900	12.12.2010	Malaise Trapping
BT_0309_Alticinae sp. 256	KJ677301	Podocarpus NP/RBSF, Ecuador	ECSF	9560580	713650	1900	12.12.2010	Malaise Trapping
BT_0311_Eumolpinae sp. 42	KJ677863	Podocarpus NP/RBSF, Ecuador	ECSF	9560580	713650	1900	12.12.2010	Malaise Trapping
BT_0312_Eumolpinae sp. 21	KJ677900	Podocarpus NP/RBSF, Ecuador	ECSF	9560580	713650	1900	12.12.2010	Malaise Trapping
BT_0314_Eumolpinae sp. 43	KJ677809	Podocarpus NP/RBSF, Ecuador	ECSF	9560580	713650	1900	12.12.2010	Malaise Trapping
BT_0336_Galerucinae sp. 56	KJ677740	Podocarpus NP/RBSF, Ecuador	Bombuscaro	9544713	725565	1020	20.11.2010	Sweep-netting
BT_0337_Galerucinae sp. 56	KJ677741	Podocarpus NP/RBSF, Ecuador	Bombuscaro	9544713	725565	1020	20.11.2010	Sweep-netting
BT_0338_Galerucinae sp. 55	KJ677742	Podocarpus NP/RBSF, Ecuador	Bombuscaro	9544713	725565	1020	20.11.2010	Sweep-netting
BT_0339_Alticinae sp. 71	KJ677330	Podocarpus NP/RBSF, Ecuador	Bombuscaro	9544713	725565	1020	20.11.2010	Sweep-netting
BT_0343_Galerucinae sp. 52	KJ677743	Podocarpus NP/RBSF, Ecuador	Bombuscaro	9544713	725565	1020	20.11.2010	Sweep-netting
BT_0346_Alticinae sp. 51	KJ677366	Podocarpus NP/RBSF, Ecuador	Bombuscaro	9544713	725565	1020	20.11.2010	Sweep-netting
BT_0349_Eumolpinae sp. 20	KJ677940	Podocarpus NP/RBSF, Ecuador	Bombuscaro	9544713	725565	1020	20.11.2010	Sweep-netting
BT_0352_Alticinae sp. 130	KJ677771	Podocarpus NP/RBSF, Ecuador	Bombuscaro	9544713	725565	1020	20.11.2010	Sweep-netting
BT_0353_Alticinae sp. 132	KJ677732	Podocarpus NP/RBSF, Ecuador	Bombuscaro	9544713	725565	1020	20.11.2010	Sweep-netting
BT_0356_Alticinae sp. 128	KJ677509	Podocarpus NP/RBSF, Ecuador	Bombuscaro	9544713	725565	1020	20.11.2010	Sweep-netting
BT_0361_Alticinae sp. 50	KJ677381	Podocarpus NP/RBSF, Ecuador	Bombuscaro	9544713	725565	1020	20.11.2010	Beating
BT_0363_Alticinae sp. 51	KJ677373	Podocarpus NP/RBSF, Ecuador	Bombuscaro	9544713	725565	1020	20.11.2010	Beating
BT_0365_Galerucinae sp. 49	KJ677645	Podocarpus NP/RBSF, Ecuador	Bombuscaro	9544713	725565	1020	20.11.2010	Beating
BT_0372_Alticinae sp. 9	KJ677723	Podocarpus NP/RBSF, Ecuador	Bombuscaro	9544713	725565	1020	20.11.2010	Hand-Coll.on Plot
BT_0375_Eumolpinae sp. 32	KJ677904	Podocarpus NP/RBSF, Ecuador	Bombuscaro	9544970	725703	1066	20.11.2010	Sweep-netting
BT_0376_Alticinae sp. 51	KJ677367	Podocarpus NP/RBSF, Ecuador	Bombuscaro	9544970	725703	1066	20.11.2010	Sweep-netting
BT_0377_Eumolpinae sp. 24	KJ677912	Podocarpus NP/RBSF, Ecuador	Bombuscaro	9544970	725703	1066	20.11.2010	Sweep-netting
BT_0380_Eumolpinae sp. 24	KJ677918	Podocarpus NP/RBSF, Ecuador	Bombuscaro	9544970	725703	1066	20.11.2010	Beating
BT_0382_Galerucinae sp. 13	KJ677537	Podocarpus NP/RBSF, Ecuador	Bombuscaro	9544970	725703	1066	20.11.2010	Hand-Coll.on Plot
BT_0383_Alticinae sp. 87	KJ677501	Podocarpus NP/RBSF, Ecuador	ECSF	9560455	713721	2002	22.11.2010	Sweep-netting
BT_0384_Eumolpinae sp. 39	KJ677883	Podocarpus NP/RBSF, Ecuador	ECSF	9560455	713721	2002	22.11.2010	Sweep-netting
BT_0387_Alticinae sp. 104	KJ677324	Podocarpus NP/RBSF, Ecuador	ECSF	9560455	713721	2002	22.11.2010	Sweep-netting
BT_0390_Alticinae sp. 90	KJ677453	Podocarpus NP/RBSF, Ecuador	ECSF	9560455	713721	2002	22.11.2010	Sweep-netting
BT_0391_Alticinae sp. 90	KJ677454	Podocarpus NP/RBSF, Ecuador	ECSF	9560455	713721	2002	22.11.2010	Sweep-netting
BT_0392_Eumolpinae sp. 74	KJ677832	Podocarpus NP/RBSF, Ecuador	ECSF	9560455	713721	2002	22.11.2010	Sweep-netting
BT_0395_Eumolpinae sp. 74	KJ677833	Podocarpus NP/RBSF, Ecuador	ECSF	9560455	713721	2002	22.11.2010	Sweep-netting
BT_0396_Cassidinae sp. 4	KJ677851	Podocarpus NP/RBSF, Ecuador	ECSF	9560455	713721	2002	22.11.2010	Beating
BT_0397_Alticinae sp. 131	KJ677654	Podocarpus NP/RBSF, Ecuador	ECSF	9560455	713721	2002	22.11.2010	Beating
BT_0399_Galerucinae sp. 28	KJ677437	Podocarpus NP/RBSF, Ecuador	Bombuscaro	9544210	724474	1268	25.11.2010	Sweep-netting
BT_0402_Galerucinae sp. 44	KJ677603	Podocarpus NP/RBSF, Ecuador	Bombuscaro	9544210	724474	1268	25.11.2010	Sweep-netting
BT_0403_Galerucinae sp. 44	KJ677604	Podocarpus NP/RBSF, Ecuador	Bombuscaro	9544210	724474	1268	25.11.2010	Sweep-netting
BT_0405_Galerucinae sp. 45	KJ677630	Podocarpus NP/RBSF, Ecuador	Bombuscaro	9544210	724474	1268	25.11.2010	Sweep-netting
BT_0407_Galerucinae sp. 49	KJ677642	Podocarpus NP/RBSF, Ecuador	Bombuscaro	9544210	724474	1268	25.11.2010	Sweep-netting
BT_0408_Eumolpinae sp. 24	KJ677913	Podocarpus NP/RBSF, Ecuador	Bombuscaro	9544210	724474	1268	25.11.2010	Sweep-netting
BT_0409_Eumolpinae sp. 24	KJ677914	Podocarpus NP/RBSF, Ecuador	Bombuscaro	9544210	724474	1268	25.11.2010	Sweep-netting
BT_0410_Alticinae sp. 141	KJ677772	Podocarpus NP/RBSF, Ecuador	Bombuscaro	9544210	724474	1268	25.11.2010	Sweep-netting

BT_0411_Alticinae sp. 58	KJ677350	Podocarpus NP/RBSF, Ecuador	Bombuscaro	9544210	724474	1268	25.11.2010	Sweep-netting
BT_0415_Alticinae sp. 242	KJ677611	Podocarpus NP/RBSF, Ecuador	Bombuscaro	9544210	724474	1268	25.11.2010	Sweep-netting
BT_0417_Galerucinae sp. 74	KJ677799	Podocarpus NP/RBSF, Ecuador	Bombuscaro	9544210	724474	1268	25.11.2010	Sweep-netting
BT_0420_Alticinae sp. 128	KJ677506	Podocarpus NP/RBSF, Ecuador	Bombuscaro	9544210	724474	1268	25.11.2010	Beating
BT_0423_Eumolpinae sp. 24	KJ677915	Podocarpus NP/RBSF, Ecuador	Bombuscaro	9544210	724474	1268	25.11.2010	Beating
BT_0425_Eumolpinae sp. 7	KJ677876	Podocarpus NP/RBSF, Ecuador	Bombuscaro	9544210	724474	1268	25.11.2010	Hand-Coll.on Plot
BT_0426_Alticinae sp. 150	KJ677715	Podocarpus NP/RBSF, Ecuador	ECSF	9560391	713726	2026	27.11.2010	Sweep-netting
BT_0427_Alticinae sp. 104	KJ677317	Podocarpus NP/RBSF, Ecuador	ECSF	9560391	713726	2026	27.11.2010	Sweep-netting
BT_0428_Alticinae sp. 105	KJ677315	Podocarpus NP/RBSF, Ecuador	ECSF	9560391	713726	2026	27.11.2010	Sweep-netting
BT_0429_Eumolpinae sp. 39	KJ677884	Podocarpus NP/RBSF, Ecuador	ECSF	9560391	713726	2026	27.11.2010	Sweep-netting
BT_0432_Eumolpinae sp. 30	KJ677902	Podocarpus NP/RBSF, Ecuador	ECSF	9560391	713726	2026	27.11.2010	Sweep-netting
BT_0433_Galerucinae sp. 69	KJ677780	Podocarpus NP/RBSF, Ecuador	ECSF	9560391	713726	2026	27.11.2010	Beating
BT_0434_Galerucinae sp. 72	KJ677805	Podocarpus NP/RBSF, Ecuador	ECSF	9560391	713726	2026	27.11.2010	Beating
BT_0436_Alticinae sp. 149	KJ677708	Podocarpus NP/RBSF, Ecuador	Bombuscaro	9544705	725544	1026	29.11.2010	Sweep-netting
BT_0438_Galerucinae sp. 15	KJ677747	Podocarpus NP/RBSF, Ecuador	Bombuscaro	9544705	725544	1026	29.11.2010	Sweep-netting
BT_0441_Galerucinae sp. 82	KJ677687	Podocarpus NP/RBSF, Ecuador	Bombuscaro	9544705	725544	1026	29.11.2010	Sweep-netting
BT_0442_Galerucinae sp. 49	KJ677647	Podocarpus NP/RBSF, Ecuador	Bombuscaro	9544705	725544	1026	29.11.2010	Sweep-netting
BT_0443_Alticinae sp. 51	KJ677368	Podocarpus NP/RBSF, Ecuador	Bombuscaro	9544705	725544	1026	29.11.2010	Beating
BT_0444_Alticinae sp. 143	KJ677574	Podocarpus NP/RBSF, Ecuador	Bombuscaro	9544705	725544	1026	29.11.2010	Beating
BT_0447_Galerucinae sp. 7	KJ677541	Podocarpus NP/RBSF, Ecuador	Bombuscaro	9544705	725544	1026	29.11.2010	Beating
BT_0448_Galerucinae sp. 49	KJ677648	Podocarpus NP/RBSF, Ecuador	Bombuscaro	9544705	725544	1026	29.11.2010	Beating
BT_0449_Galerucinae sp. 49	KJ677646	Podocarpus NP/RBSF, Ecuador	Bombuscaro	9544705	725544	1026	29.11.2010	Beating
BT_0451_Galerucinae sp. 15	KJ677748	Podocarpus NP/RBSF, Ecuador	Bombuscaro	9544705	725544	1026	29.11.2010	Hand-Coll.on Plot
BT_0452_Alticinae sp. 9	KJ677722	Podocarpus NP/RBSF, Ecuador	Bombuscaro	9544705	725544	1026	29.11.2010	Hand-Coll.on Plot
BT_0454_Alticinae sp. 26	KJ677408	Podocarpus NP/RBSF, Ecuador	Bombuscaro	9544734	725457	1046	29.11.2010	Sweep-netting
BT_0457_Alticinae sp. 181	KJ677279	Podocarpus NP/RBSF, Ecuador	Bombuscaro	9544734	725457	1046	29.11.2010	Sweep-netting
BT_0459_Galerucinae sp. 50	KJ677432	Podocarpus NP/RBSF, Ecuador	Bombuscaro	9544734	725457	1046	29.11.2010	Sweep-netting
BT_0460_Galerucinae sp. 45	KJ677629	Podocarpus NP/RBSF, Ecuador	Bombuscaro	9544734	725457	1046	29.11.2010	Sweep-netting
BT_0461_Galerucinae sp. 45	KJ677628	Podocarpus NP/RBSF, Ecuador	Bombuscaro	9544734	725457	1046	29.11.2010	Sweep-netting
BT_0462_Eumolpinae sp. 42	KJ677858	Podocarpus NP/RBSF, Ecuador	Bombuscaro	9544734	725457	1046	29.11.2010	Beating
BT_0463_Galerucinae sp. 70	KJ677803	Podocarpus NP/RBSF, Ecuador	Bombuscaro	9544734	725457	1046	29.11.2010	Beating
BT_0465_Alticinae sp. 50	KJ677382	Podocarpus NP/RBSF, Ecuador	Bombuscaro	9544734	725457	1046	29.11.2010	Beating
BT_0468_Alticinae sp. 72	KJ677620	Podocarpus NP/RBSF, Ecuador	Bombuscaro	9544734	725457	1046	29.11.2010	Beating
BT_0469_Galerucinae sp. 45	KJ677631	Podocarpus NP/RBSF, Ecuador	Bombuscaro	9544734	725457	1046	29.11.2010	Beating
BT_0473_Galerucinae sp. 69	KJ677779	Podocarpus NP/RBSF, Ecuador	ECSF	9560455	713721	2002	01.12.2010	Sweep-netting
BT_0474_Alticinae sp. 80	KJ677615	Podocarpus NP/RBSF, Ecuador	ECSF	9560455	713721	2002	01.12.2010	Sweep-netting
BT_0475_Eumolpinae sp. 39	KJ677885	Podocarpus NP/RBSF, Ecuador	ECSF	9560455	713721	2002	01.12.2010	Sweep-netting
BT_0489_Galerucinae sp. 67	KJ677802	Podocarpus NP/RBSF, Ecuador	ECSF	9560455	713721	2002	01.12.2010	Beating
BT_0490_Hispinae sp. 5	KJ677839	Podocarpus NP/RBSF, Ecuador	ECSF	9560455	713721	2002	01.12.2010	Beating
BT_0491_Alticinae sp. 157	KJ677597	Podocarpus NP/RBSF, Ecuador	ECSF	9560307	713655	1993	02.12.2010	Sweep-netting
BT_0492_Galerucinae sp. 61	KJ677515	Podocarpus NP/RBSF, Ecuador	ECSF	9560307	713655	1993	02.12.2010	Sweep-netting
BT_0494_Alticinae sp. 250	KJ677450	Podocarpus NP/RBSF, Ecuador	ECSF	9560307	713655	1993	02.12.2010	Sweep-netting
BT_0496_Alticinae sp. 112	KJ677782	Podocarpus NP/RBSF, Ecuador	ECSF	9560307	713655	1993	02.12.2010	Sweep-netting
BT_0499_Alticinae sp. 44	KJ677375	Podocarpus NP/RBSF, Ecuador	ECSF	9560307	713655	1993	02.12.2010	Beating
BT_0501_Galerucinae sp. 66	KJ677795	Podocarpus NP/RBSF, Ecuador	ECSF	9560307	713655	1993	02.12.2010	Beating
BT_0502_Eumolpinae sp. 42	KJ677864	Podocarpus NP/RBSF, Ecuador	ECSF	9560307	713655	1993	02.12.2010	Beating
BT_0503_Alticinae sp. 111	KJ677601	Podocarpus NP/RBSF, Ecuador	ECSF	9560307	713655	1993	02.12.2010	Beating
BT_0505_Alticinae sp. 96	KJ677470	Podocarpus NP/RBSF, Ecuador	ECSF	9560307	713655	1993	02.12.2010	Hand-Coll.on Plot
BT_0506_Alticinae sp. 92	KJ677737	Podocarpus NP/RBSF, Ecuador	ECSF	9560307	713655	1993	02.12.2010	Hand-Coll.on Plot
BT_0508_Galerucinae sp. 53	KJ677757	Podocarpus NP/RBSF, Ecuador	ECSF	9560307	713655	1993	02.12.2010	Hand-Coll.on Plot
BT_0510_Cassidinae sp. 4	KJ677852	Podocarpus NP/RBSF, Ecuador	ECSF	9560307	713655	1993	02.12.2010	Hand-Coll.on Plot
BT_0511_Cassidinae sp. 8	KJ677821	Podocarpus NP/RBSF, Ecuador	ECSF	9560307	713655	1993	02.12.2010	Hand-Coll.on Plot
BT_0512_Hispinae sp. 6	KJ677847	Podocarpus NP/RBSF, Ecuador	ECSF	9560307	713655	1993	02.12.2010	Hand-Coll.on Plot
BT_0514_Eumolpinae sp. 74	KJ677834	Podocarpus NP/RBSF, Ecuador	ECSF	9560143	713666	2089	03.12.2010	Sweep-netting
BT_0516_Alticinae sp. 126	KJ677783	Podocarpus NP/RBSF, Ecuador	ECSF	9560143	713666	2089	03.12.2010	Sweep-netting
BT_0517_Alticinae sp. 104	KJ677325	Podocarpus NP/RBSF, Ecuador	ECSF	9560143	713666	2089	03.12.2010	Sweep-netting
BT_0518_Alticinae sp. 85	KJ677388	Podocarpus NP/RBSF, Ecuador	ECSF	9560143	713666	2089	03.12.2010	Sweep-netting
BT_0519_Alticinae sp. 68	KJ677462	Podocarpus NP/RBSF, Ecuador	ECSF	9560143	713666	2089	03.12.2010	Beating
BT_0520_Alticinae sp. 159	KJ677595	Podocarpus NP/RBSF, Ecuador	ECSF	9560143	713666	2089	03.12.2010	Beating
BT_0524_Eumolpinae sp. 39	KJ677895	Podocarpus NP/RBSF, Ecuador	ECSF	9560143	713666	2089	03.12.2010	Beating
BT_0525_Hispinae sp. 23	KJ677844	Podocarpus NP/RBSF, Ecuador	ECSF	9560143	713666	2089	03.12.2010	Beating
BT_0526_Eumolpinae sp. 39	KJ677886	Podocarpus NP/RBSF, Ecuador	ECSF	9560143	713666	2089	03.12.2010	Hand-Coll.on Plot
BT_0527_Galerucinae sp. 36	KJ677761	Podocarpus NP/RBSF, Ecuador	ECSF	9560235	713688	2039	08.12.2010	Sweep-netting
BT_0528_Alticinae sp. 150	KJ677714	Podocarpus NP/RBSF, Ecuador	ECSF	9560235	713688	2039	08.12.2010	Sweep-netting
BT_0529_Alticinae sp. 113	KJ677755	Podocarpus NP/RBSF, Ecuador	ECSF	9560235	713688	2039	08.12.2010	Sweep-netting
BT_0530_Galerucinae sp. 31	KJ677750	Podocarpus NP/RBSF, Ecuador	ECSF	9560235	713688	2039	08.12.2010	Beating
BT_0531_Galerucinae sp. 62	KJ677758	Podocarpus NP/RBSF, Ecuador	ECSF	9560235	713688	2039	08.12.2010	Beating
BT_0532_Galerucinae sp. 75	KJ677800	Podocarpus NP/RBSF, Ecuador	ECSF	9560235	713688	2039	08.12.2010	Beating
BT_0533_Alticinae sp. 97	KJ677302	Podocarpus NP/RBSF, Ecuador	ECSF	9560235	713688	2039	08.12.2010	Beating
BT_0535_Alticinae sp. 97	KJ677310	Podocarpus NP/RBSF, Ecuador	ECSF	9560235	713688	2039	08.12.2010	Hand-Coll.on Plot
BT_0537_Alticinae sp. 142	KJ677591	Podocarpus NP/RBSF, Ecuador	ECSF	9560245	713695	2030	08.12.2010	Sweep-netting
BT_0538_Alticinae sp. 238	KJ677679	Podocarpus NP/RBSF, Ecuador	ECSF	9560245	713695	2030	08.12.2010	Sweep-netting
BT_0539_Alticinae sp. 13	KJ677676	Podocarpus NP/RBSF, Ecuador	ECSF	9560245	713695	2030	08.12.2010	Beating
BT_0540_Galerucinae sp. 64	KJ677788	Podocarpus NP/RBSF, Ecuador	ECSF	9560245	713695	2030	08.12.2010	Beating
BT_0544_Cassidinae sp. 12	KJ677824	Podocarpus NP/RBSF, Ecuador	ECSF	9560245	713695	2030	08.12.2010	Hand-Coll.on Plot
BT_0546_Alticinae sp. 83	KJ677335	Podocarpus NP/RBSF, Ecuador	ECSF	9560423	714258	1933	09.12.2010	Sweep-netting
BT_0547_Alticinae sp. 96	KJ677471	Podocarpus NP/RBSF, Ecuador	ECSF	9560423	714258	1933	09.12.2010	Sweep-netting
BT_0549_Alticinae sp. 150	KJ677716	Podocarpus NP/RBSF, Ecuador	ECSF	9560423	714258	1933	09.12.2010	Sweep-netting
BT_0550_Alticinae sp. 265	KJ677429	Podocarpus NP/RBSF, Ecuador	ECSF	9560423	714258	1933	09.12.2010	Sweep-netting
BT_0551_Galerucinae sp. 64	KJ677789	Podocarpus NP/RBSF, Ecuador	ECSF	9560423	714258	1933	09.12.2010	Beating
BT_0552_Alticinae sp. 117	KJ677492	Podocarpus NP/RBSF, Ecuador	ECSF	9560423	714258	1933	09.12.2010	Beating
BT_0553_Eumolpinae sp. 42	KJ677865	Podocarpus NP/RBSF, Ecuador	ECSF	9560423	714258	1933	09.12.2010	Beating
BT_0554_Galerucinae sp. 71	KJ677808	Podocarpus NP/RBSF, Ecuador	ECSF	9560423	714258	1933	09.12.2010	Beating
BT_0555_Alticinae sp. 86	KJ677403	Podocarpus NP/RBSF, Ecuador	ECSF	9560467	714261	1954	09.12.2010	Sweep-netting
BT_0556_Alticinae sp. 96	KJ677472	Podocarpus NP/RBSF, Ecuador	ECSF	9560467	714261	1954	09.12.2010	Sweep-netting
BT_0557_Alticinae sp. 181	KJ677784	Podocarpus NP/RBSF, Ecuador	ECSF	9560467	714261	1954	09.12.2010	Sweep-netting

BT_0558_Alticinae sp. 149	KJ677718	Podocarpus NP/RBSF, Ecuador	ECSF	9560467	714261	1954	09.12.2010	Beating
BT_0559_Eumolpinae sp. 42	KJ677866	Podocarpus NP/RBSF, Ecuador	ECSF	9560467	714261	1954	09.12.2010	Beating
BT_0560_Galerucinae sp. 64	KJ677790	Podocarpus NP/RBSF, Ecuador	ECSF	9560467	714261	1954	09.12.2010	Beating
BT_0561_Galerucinae sp. 66	KJ677797	Podocarpus NP/RBSF, Ecuador	ECSF	9560467	714261	1954	09.12.2010	Beating
BT_0565_Alticinae sp. 150	KJ677712	Podocarpus NP/RBSF, Ecuador	ECSF	9560467	714261	1954	09.12.2010	Hand-Coll.on Plot
BT_0566_Hispinae sp. 6	KJ677848	Podocarpus NP/RBSF, Ecuador	ECSF	9560467	714261	1954	09.12.2010	Hand-Coll.on Plot
BT_0567_Alticinae sp. 104	KJ677326	Podocarpus NP/RBSF, Ecuador	ECSF	9560354	714028	2054	11.12.2010	Sweep-netting
BT_0574_Eumolpinae sp. 39	KJ677887	Podocarpus NP/RBSF, Ecuador	ECSF	9560354	714028	2054	11.12.2010	Beating
BT_0575_Alticinae sp. 140	KJ677570	Podocarpus NP/RBSF, Ecuador	ECSF	9560354	714028	2054	11.12.2010	Hand-Coll.on Plot
BT_0577_Eumolpinae sp. 39	KJ677888	Podocarpus NP/RBSF, Ecuador	ECSF	9560321	714040	2063	11.12.2010	Sweep-netting
BT_0579_Alticinae sp. 104	KJ677321	Podocarpus NP/RBSF, Ecuador	ECSF	9560321	714040	2063	11.12.2010	Sweep-netting
BT_0587_Alticinae sp. 6	KJ677583	Podocarpus NP/RBSF, Ecuador	ECSF	9560321	714040	2063	11.12.2010	Beating
BT_0588_Galerucinae sp. 69	KJ677781	Podocarpus NP/RBSF, Ecuador	ECSF	9560321	714040	2063	11.12.2010	Beating
BT_0589_Eumolpinae sp. 17	KJ677910	Podocarpus NP/RBSF, Ecuador	ECSF	9560321	714040	2063	11.12.2010	Beating
BT_0590_Alticinae sp. 140	KJ677571	Podocarpus NP/RBSF, Ecuador	ECSF	9560321	714040	2063	11.12.2010	Beating
BT_0592_Eumolpinae sp. 39	KJ677889	Podocarpus NP/RBSF, Ecuador	ECSF	9560321	714040	2063	11.12.2010	Beating
BT_0594_Alticinae sp. 118	KJ677663	Podocarpus NP/RBSF, Ecuador	ECSF	9560321	714040	2063	11.12.2010	Hand-Coll.on Plot
BT_0596_Eumolpinae sp. 39	KJ677890	Podocarpus NP/RBSF, Ecuador	ECSF	9560383	714029	2039	14.12.2010	Sweep-netting
BT_0597_Alticinae sp. 104	KJ677322	Podocarpus NP/RBSF, Ecuador	ECSF	9560383	714029	2039	14.12.2010	Sweep-netting
BT_0604_Hispinae sp. 5	KJ677840	Podocarpus NP/RBSF, Ecuador	ECSF	9560383	714029	2039	14.12.2010	Sweep-netting
BT_0605_Alticinae sp. 87	KJ677498	Podocarpus NP/RBSF, Ecuador	ECSF	9560383	714029	2039	14.12.2010	Beating
BT_0606_Eumolpinae sp. 39	KJ677891	Podocarpus NP/RBSF, Ecuador	ECSF	9560383	714029	2039	14.12.2010	Beating
BT_0628_Cassidinae sp. 12	KJ677825	Podocarpus NP/RBSF, Ecuador	ECSF	9560505	714170	1913	14.12.2010	Sweep-netting
BT_0630_Alticinae sp. 36	KJ677610	Podocarpus NP/RBSF, Ecuador	ECSF	9560505	714170	1913	14.12.2010	Sweep-netting
BT_0631_Alticinae sp. 64	KJ677451	Podocarpus NP/RBSF, Ecuador	ECSF	9560505	714170	1913	14.12.2010	Sweep-netting
BT_0632_Alticinae sp. 86	KJ677392	Podocarpus NP/RBSF, Ecuador	ECSF	9560505	714170	1913	14.12.2010	Sweep-netting
BT_0633_Alticinae sp. 85	KJ677404	Podocarpus NP/RBSF, Ecuador	ECSF	9560505	714170	1913	14.12.2010	Sweep-netting
BT_0634_Alticinae sp. 66	KJ677473	Podocarpus NP/RBSF, Ecuador	ECSF	9560505	714170	1913	14.12.2010	Sweep-netting
BT_0635_Alticinae sp. 96	KJ677474	Podocarpus NP/RBSF, Ecuador	ECSF	9560505	714170	1913	14.12.2010	Sweep-netting
BT_0640_Alticinae sp. 18	KJ677425	Podocarpus NP/RBSF, Ecuador	ECSF	9560505	714170	1913	14.12.2010	Beating
BT_0641_Alticinae sp. 13	KJ677674	Podocarpus NP/RBSF, Ecuador	ECSF	9560505	714170	1913	14.12.2010	Beating
BT_0642_Eumolpinae sp. 42	KJ677859	Podocarpus NP/RBSF, Ecuador	ECSF	9560505	714170	1913	14.12.2010	Beating
BT_0644_Galerucinae sp. 66	KJ677796	Podocarpus NP/RBSF, Ecuador	ECSF	9560505	714170	1913	14.12.2010	Beating
BT_0645_Galerucinae sp. 11	KJ677552	Podocarpus NP/RBSF, Ecuador	ECSF	9560505	714170	1913	14.12.2010	Hand-Coll.on Plot
BT_0646_Alticinae sp. 71	KJ677379	Podocarpus NP/RBSF, Ecuador	ECSF	9560505	714170	1913	14.12.2010	Hand-Coll.on Plot
BT_0647_Galerucinae sp. 35	KJ677660	Podocarpus NP/RBSF, Ecuador	ECSF	9560505	714170	1913	14.12.2010	Hand-Coll.on Plot
BT_0648_Alticinae sp. 140	KJ677568	Podocarpus NP/RBSF, Ecuador	ECSF	9560455	713721	2002	15.12.2010	Sweep-netting
BT_0650_Eumolpinae sp. 39	KJ677892	Podocarpus NP/RBSF, Ecuador	ECSF	9560455	713721	2002	15.12.2010	Sweep-netting
BT_0652_Alticinae sp. 104	KJ677318	Podocarpus NP/RBSF, Ecuador	ECSF	9560455	713721	2002	15.12.2010	Sweep-netting
BT_0655_Alticinae sp. 257	KJ677449	Podocarpus NP/RBSF, Ecuador	ECSF	9560455	713721	2002	15.12.2010	Sweep-netting
BT_0656_Alticinae sp. 51	KJ677351	Podocarpus NP/RBSF, Ecuador	ECSF	9560455	713721	2002	15.12.2010	Beating
BT_0657_Eumolpinae sp. 39	KJ677893	Podocarpus NP/RBSF, Ecuador	ECSF	9560455	713721	2002	15.12.2010	Beating
BT_0660_Alticinae sp. 104	KJ677327	Podocarpus NP/RBSF, Ecuador	ECSF	9560455	713721	2002	15.12.2010	Beating
BT_0662_Alticinae sp. 126	KJ677313	Podocarpus NP/RBSF, Ecuador	ECSF	9560455	713721	2002	15.12.2010	Beating
BT_0663_Alticinae sp. 150	KJ677713	Podocarpus NP/RBSF, Ecuador	ECSF	9560455	713721	2002	15.12.2010	Beating
BT_0664_Alticinae sp. 150	KJ677720	Podocarpus NP/RBSF, Ecuador	ECSF	9560455	713721	2002	15.12.2010	Beating
BT_0665_Alticinae sp. 150	KJ677717	Podocarpus NP/RBSF, Ecuador	ECSF	9560455	713721	2002	15.12.2010	Beating
BT_0675_Alticinae sp. 8	KJ677710	Podocarpus NP/RBSF, Ecuador	ECSF	9560455	713721	2002	15.12.2010	Beating
BT_0676_Alticinae sp. 64	KJ677457	Podocarpus NP/RBSF, Ecuador	ECSF	9560455	713721	2002	15.12.2010	Hand-Coll.on Plot
BT_0677_Eumolpinae sp. 39	KJ677894	Podocarpus NP/RBSF, Ecuador	ECSF	9560455	713721	2002	15.12.2010	Hand-Coll.on Plot
BT_0680_Galerucinae sp. 76	KJ677560	Podocarpus NP/RBSF, Ecuador	ECSF	9560781.7	713299.6	1826	21.03.2011	Light trapping
BT_0683_Alticinae sp. 115	KJ677298	Podocarpus NP/RBSF, Ecuador	ECSF	9560781.7	713299.6	1826	21.03.2011	Light trapping
BT_0686_Eumolpinae sp. 23	KJ677901	Podocarpus NP/RBSF, Ecuador	ECSF	9560352	713911	2011	22.03.2011	Hand-Collection
BT_0687_Alticinae sp. 87	KJ677499	Podocarpus NP/RBSF, Ecuador	ECSF	9560352	713911	2011	22.03.2011	Hand-Collection
BT_0688_Criocerinae sp. 1	KJ677815	Podocarpus NP/RBSF, Ecuador	ECSF	9560352	713911	2011	22.03.2011	Hand-Collection
BT_0690_Alticinae sp. 124	KJ677493	Podocarpus NP/RBSF, Ecuador	ECSF	9560352	713911	2011	22.03.2011	Hand-Collection
BT_0691_Galerucinae sp. 31	KJ677753	Podocarpus NP/RBSF, Ecuador	ECSF	9560352	713911	2011	22.03.2011	Hand-Collection
BT_0692_Alticinae sp. 14	KJ677588	Podocarpus NP/RBSF, Ecuador	ECSF	9560352	713911	2011	22.03.2011	Hand-Collection
BT_0698_Alticinae sp. 13	KJ677675	Podocarpus NP/RBSF, Ecuador	ECSF	9559929	712405	1800	22.03.2011	Light trapping
BT_0699_Alticinae sp. 83	KJ677336	Podocarpus NP/RBSF, Ecuador	ECSF	9559929	712405	1800	22.03.2011	Light trapping
BT_0705_Galerucinae sp. 8	KJ677539	Podocarpus NP/RBSF, Ecuador	Bombuscaro	NA	NA	2170	23.03.2011	Light trapping
BT_0709_Galerucinae sp. 64	KJ677791	Podocarpus NP/RBSF, Ecuador	ECSF	9560352	713911	2180	24.03.2011	Light trapping
BT_0710_Alticinae sp. 13	KJ677678	Podocarpus NP/RBSF, Ecuador	ECSF	9560200	713660	2000	25.03.2011	Light trapping
BT_0711_Galerucinae sp. 73	KJ677785	Podocarpus NP/RBSF, Ecuador	Cajanuma	9545638	702126	2863	26.03.2011	Light trapping
BT_0713_Galerucinae sp. 73	KJ677786	Podocarpus NP/RBSF, Ecuador	Cajanuma	9545638	702126	2863	26.03.2011	Light trapping
BT_0715_Galerucinae sp. 76	KJ677561	Podocarpus NP/RBSF, Ecuador	ECSF	9560781.7	713299.6	1826	28.03.2011	Hand-Collection
BT_0716_Galerucinae sp. 11	KJ677534	Podocarpus NP/RBSF, Ecuador	ECSF	9560781.7	713299.6	1826	28.03.2011	Hand-Collection
BT_0717_Galerucinae sp. 2	KJ677553	Podocarpus NP/RBSF, Ecuador	ECSF	9560781.7	713299.6	1826	28.03.2011	Hand-Collection
BT_0719_Eumolpinae sp. 43	KJ677810	Podocarpus NP/RBSF, Ecuador	ECSF	9560352	713911	1800	29.03.2011	Light trapping
BT_0721_Eumolpinae sp. 20	KJ677945	Podocarpus NP/RBSF, Ecuador	ECSF	9560352	713911	1800	29.03.2011	Light trapping
BT_0722_Cassidinae sp. 13	KJ677811	Podocarpus NP/RBSF, Ecuador	Bombuscaro	9544506	725125	1025	30.03.2011	Light trapping
BT_0725_Alticinae sp. 243	KJ677416	Podocarpus NP/RBSF, Ecuador	ECSF*	9560352	711647	2670	31.03.2011	Light trapping
BT_0727_Galerucinae sp. 73	KJ677787	Podocarpus NP/RBSF, Ecuador	ECSF*	9560352	711647	2670	31.03.2011	Light trapping
BT_0728_Galerucinae sp. 64	KJ677792	Podocarpus NP/RBSF, Ecuador	ECSF*	9560352	711647	2670	31.03.2011	Light trapping
BT_0729_Galerucinae sp. 15	KJ677749	Podocarpus NP/RBSF, Ecuador	Bombuscaro	9544506	725125	1025	01.04.2011	Light trapping
BT_0730_Alticinae sp. 136	KJ677658	Podocarpus NP/RBSF, Ecuador	Bombuscaro	9544506	725125	1025	01.04.2011	Light trapping
BT_0732_Galerucinae sp. 4	KJ677538	Podocarpus NP/RBSF, Ecuador	Bombuscaro	9544506	725125	1104	01.04.2011	Light trapping
BT_0733_Galerucinae sp. 97	KJ677540	Podocarpus NP/RBSF, Ecuador	Bombuscaro	9544506	725125	1104	01.04.2011	Light trapping
BT_0734_Galerucinae sp. 39	KJ677524	Podocarpus NP/RBSF, Ecuador	ECSF	9560200	713660	2000	02.04.2011	Light trapping
BT_0735_Galerucinae sp. 38	KJ677527	Podocarpus NP/RBSF, Ecuador	El Tiro	NA	NA	2590	03.04.2011	Light trapping
BT_0736_Galerucinae sp. 26	KJ677768	Podocarpus NP/RBSF, Ecuador	ECSF	9560445	714260	1963 m	04.04.2011	Light trapping
BT_0738_Eumolpinae sp. 4	KJ677905	Podocarpus NP/RBSF, Ecuador	ECSF	9560781.7	713299.6	1826	May 2011	Hand-Collection
BT_0739_Galerucinae sp. 18	KJ677522	Podocarpus NP/RBSF, Ecuador	ECSF	9560781.7	713299.6	1826	May 2011	Hand-Collection
BT_0740_Galerucinae sp. 76	KJ677562	Podocarpus NP/RBSF, Ecuador	ECSF	9560781.7	713299.6	1826	May 2011	Hand-Collection
BT_0741_Galerucinae sp. 2	KJ677549	Podocarpus NP/RBSF, Ecuador	ECSF	9560781.7	713299.6	1826	May 2011	Hand-Collection
BT_0742_Galerucinae sp. 19	KJ677565	Podocarpus NP/RBSF, Ecuador	ECSF	9560781.7	713299.6	1826	May 2011	Hand-Collection

BT_0743_Galerucinae sp. 14	KJ677531	Podocarpus NP/RBSF, Ecuador	ECSF	9560781.7	713299.6	1826	May 2011	Hand-Collection
BT_0744_Galerucinae sp. 7	KJ677544	Podocarpus NP/RBSF, Ecuador	ECSF	9560781.7	713299.6	1826	May 2011	Hand-Collection
BT_0747_Galerucinae sp. 38	KJ677528	Podocarpus NP/RBSF, Ecuador	ECSF	9560781.7	713299.6	1826	May 2011	Hand-Collection
BT_0749_Galerucinae sp. 39	KJ677525	Podocarpus NP/RBSF, Ecuador	ECSF	9560781.7	713299.6	1826	May 2011	Hand-Collection
BT_0750_Cassidinae sp. 5	KJ677823	Podocarpus NP/RBSF, Ecuador	ECSF	9560352	713911	2011	08.05.2011	Hand-Collection
BT_0752_Alticinae sp. 39	KJ677653	Podocarpus NP/RBSF, Ecuador	ECSF	9560352	713911	2011	08.05.2011	Hand-Collection
BT_0753_Alticinae sp. 10	KJ677731	Podocarpus NP/RBSF, Ecuador	ECSF	9560352	713911	2011	08.05.2011	Hand-Collection
BT_0755_Galerucinae sp. 36	KJ677760	Podocarpus NP/RBSF, Ecuador	ECSF	9560352	713911	2011	08.05.2011	Hand-Collection
BT_0756_Eumolpinae sp. 22	KJ677896	Podocarpus NP/RBSF, Ecuador	ECSF	9560352	713911	2011	08.05.2011	Hand-Collection
BT_0766_Alticinae sp. 96	KJ677475	Podocarpus NP/RBSF, Ecuador	ECSF	9560352	713911	2011	08.05.2011	Hand-Collection
BT_0770_Eumolpinae sp. 16	KJ677937	Podocarpus NP/RBSF, Ecuador	ECSF	9560352	713911	2011	08.05.2011	Hand-Collection
BT_0779_Alticinae sp. 115	KJ677290	Podocarpus NP/RBSF, Ecuador	ECSF	9560352	713911	2011	08.05.2011	Hand-Collection
BT_0781_Eumolpinae sp. 20	KJ677942	Podocarpus NP/RBSF, Ecuador	ECSF	9560352	713911	2011	08.05.2011	Hand-Collection
BT_0782_Galerucinae sp. 34	KJ677695	Podocarpus NP/RBSF, Ecuador	ECSF	9560352	713911	2011	08.05.2011	Hand-Collection
BT_0788_Alticinae sp. 6	KJ677580	Podocarpus NP/RBSF, Ecuador	ECSF	9560352	713911	2011	10.05.2011	Hand-Collection
BT_0789_Alticinae sp.14	KJ677589	Podocarpus NP/RBSF, Ecuador	ECSF	9560352	713911	2011	10.05.2011	Hand-Collection
BT_0791_Eumolpinae sp. 16	KJ677935	Podocarpus NP/RBSF, Ecuador	ECSF	9560352	713911	2011	10.05.2011	Hand-Collection
BT_0792_Alticinae sp. 115	KJ677291	Podocarpus NP/RBSF, Ecuador	ECSF	9560352	713911	2011	10.05.2011	Hand-Collection
BT_0794_Alticinae sp. 129	KJ677770	Podocarpus NP/RBSF, Ecuador	ECSF	9560352	713911	2011	10.05.2011	Hand-Collection
BT_0795_Alticinae sp. 96	KJ677463	Podocarpus NP/RBSF, Ecuador	ECSF	9560352	713911	2011	10.05.2011	Hand-Collection
BT_0796_Alticinae sp. 97	KJ677305	Podocarpus NP/RBSF, Ecuador	ECSF	9560352	713911	2011	10.05.2011	Hand-Collection
BT_0799_Cassidinae sp. 14	KJ677875	Podocarpus NP/RBSF, Ecuador	ECSF	9560352	713911	2011	12.05.2011	Hand-Collection
BT_0800_Galerucinae sp. 30	KJ677702	Podocarpus NP/RBSF, Ecuador	ECSF	9560352	713911	2011	12.05.2011	Hand-Collection
BT_0803_Galerucinae sp. 76	KJ677563	Podocarpus NP/RBSF, Ecuador	ECSF	9560352	713911	2011	12.05.2011	Hand-Collection
BT_0804_Alticinae sp. 28	KJ677347	Podocarpus NP/RBSF, Ecuador	ECSF	9560352	713911	2011	12.05.2011	Hand-Collection
BT_0805_Alticinae sp. 14	KJ677590	Podocarpus NP/RBSF, Ecuador	ECSF	9560352	713911	2011	12.05.2011	Hand-Collection
BT_0806_Alticinae sp. 13	KJ677677	Podocarpus NP/RBSF, Ecuador	ECSF	9560352	713911	2011	12.05.2011	Hand-Collection
BT_0807_Alticinae sp. 54	KJ677378	Podocarpus NP/RBSF, Ecuador	ECSF	9560352	713911	2011	12.05.2011	Hand-Collection
BT_0809_Alticinae sp. 12	KJ677622	Podocarpus NP/RBSF, Ecuador	ECSF	9560352	713911	2011	12.05.2011	Hand-Collection
BT_0810_Galerucinae sp. 46	KJ677637	Podocarpus NP/RBSF, Ecuador	ECSF	9560352	713911	2011	12.05.2011	Hand-Collection
BT_0811_Criocerinae sp. 6	KJ677817	Podocarpus NP/RBSF, Ecuador	ECSF	9560352	713911	2011	12.05.2011	Hand-Collection
BT_0813_Criocerinae sp. 4	KJ677818	Podocarpus NP/RBSF, Ecuador	ECSF	9560352	713911	2011	12.05.2011	Hand-Collection
BT_0814_Alticinae sp. 86	KJ677405	Podocarpus NP/RBSF, Ecuador	ECSF	9560352	713911	2011	12.05.2011	Hand-Collection
BT_0815_Alticinae sp. 32	KJ677623	Podocarpus NP/RBSF, Ecuador	ECSF	9560352	713911	2011	12.05.2011	Hand-Collection
BT_0816_Galerucinae sp. 19	KJ677566	Podocarpus NP/RBSF, Ecuador	ECSF	9560352	713911	2011	12.05.2011	Hand-Collection
BT_0817_Alticinae sp. 87	KJ677502	Podocarpus NP/RBSF, Ecuador	ECSF	9560352	713911	2011	12.05.2011	Hand-Collection
BT_0818_Galerucinae sp. 61	KJ677516	Podocarpus NP/RBSF, Ecuador	ECSF	9560352	713911	2011	12.05.2011	Hand-Collection
BT_0819_Eumolpinae sp. 74	KJ677836	Podocarpus NP/RBSF, Ecuador	ECSF	9560352	713911	2011	12.05.2011	Hand-Collection
BT_0820_Eumolpinae sp. 38	KJ677928	Podocarpus NP/RBSF, Ecuador	ECSF	9560352	713911	2011	12.05.2011	Hand-Collection
BT_0823_Galerucinae sp. 34	KJ677690	Podocarpus NP/RBSF, Ecuador	ECSF	9560352	713911	2011	12.05.2011	Hand-Collection
BT_0827_Eumolpinae sp. 20	KJ677943	Podocarpus NP/RBSF, Ecuador	ECSF	9560352	713911	2011	12.05.2011	Hand-Collection
BT_0828_Alticinae sp. 118	KJ677664	Podocarpus NP/RBSF, Ecuador	ECSF	9560352	713911	2011	12.05.2011	Hand-Collection
BT_0829_Alticinae sp. 115	KJ677292	Podocarpus NP/RBSF, Ecuador	ECSF	9560352	713911	2011	12.05.2011	Hand-Collection
BT_0831_Alticinae sp. 147	KJ677439	Podocarpus NP/RBSF, Ecuador	ECSF	9560352	713911	2011	12.05.2011	Hand-Collection
BT_0835_Galerucinae sp. 29	KJ677519	Podocarpus NP/RBSF, Ecuador	Bombuscaro	9544506	725125	1104	14.05.2011	Hand-Collection
BT_0836_Alticinae sp. 3	KJ677703	Podocarpus NP/RBSF, Ecuador	Bombuscaro	9544506	725125	1104	14.05.2011	Hand-Collection
BT_0837_Alticinae sp. 9	KJ677724	Podocarpus NP/RBSF, Ecuador	Bombuscaro	9544506	725125	1104	14.05.2011	Hand-Collection
BT_0839_Alticinae sp. 40	KJ677420	Podocarpus NP/RBSF, Ecuador	Bombuscaro	9544506	725125	1104	14.05.2011	Hand-Collection
BT_0840_Alticinae sp. 140	KJ677575	Podocarpus NP/RBSF, Ecuador	Bombuscaro	9544506	725125	1104	14.05.2011	Hand-Collection
BT_0841_Alticinae sp. 127	KJ677277	Podocarpus NP/RBSF, Ecuador	Bombuscaro	9544506	725125	1104	14.05.2011	Hand-Collection
BT_0843_Alticinae sp. 136	KJ677659	Podocarpus NP/RBSF, Ecuador	Bombuscaro	9544506	725125	1104	14.05.2011	Hand-Collection
BT_0847_Eumolpinae sp. 5	KJ677933	Podocarpus NP/RBSF, Ecuador	ECSF	9560580	713650	1900	15.05.2011	Malaise Trapping
BT_0848_Alticinae sp. 45	KJ677419	Podocarpus NP/RBSF, Ecuador	ECSF	9560580	713650	1900	15.05.2011	Malaise Trapping
BT_0850_Alticinae sp. 49	KJ677607	Podocarpus NP/RBSF, Ecuador	ECSF	9560580	713650	1900	15.05.2011	Malaise Trapping
BT_0851_Alticinae sp. 65	KJ677476	Podocarpus NP/RBSF, Ecuador	ECSF	9560580	713650	1900	15.05.2011	Malaise Trapping
BT_0852_Alticinae sp. 133	KJ677586	Podocarpus NP/RBSF, Ecuador	ECSF	9560580	713650	1900	15.05.2011	Malaise Trapping
BT_0855_Alticinae sp. 18	KJ677426	Podocarpus NP/RBSF, Ecuador	ECSF	9560580	713650	1900	15.05.2011	Malaise Trapping
BT_0861_Eumolpinae sp. 20	KJ677944	Podocarpus NP/RBSF, Ecuador	ECSF	9560580	713650	1900	15.05.2011	Malaise Trapping
BT_0862_Alticinae sp. 115	KJ677293	Podocarpus NP/RBSF, Ecuador	ECSF	9560580	713650	1900	15.05.2011	Malaise Trapping
BT_0864_Alticinae sp. 253	KJ677306	Podocarpus NP/RBSF, Ecuador	ECSF	9560580	713650	1900	15.05.2011	Malaise Trapping
BT_0865_Alticinae sp. 96	KJ677477	Podocarpus NP/RBSF, Ecuador	ECSF	9560580	713650	1900	15.05.2011	Malaise Trapping
BT_0871_Alticinae sp. 86	KJ677391	Podocarpus NP/RBSF, Ecuador	ECSF	9560580	713650	1900	15.05.2011	Malaise Trapping
BT_0873_Alticinae sp. 85	KJ677397	Podocarpus NP/RBSF, Ecuador	ECSF	9560580	713650	1900	15.05.2011	Malaise Trapping
BT_0949_Cassidinae sp. 2	KJ677871	Podocarpus NP/RBSF, Ecuador	Bombuscaro	9544506	725125	1104	20.05.2011	Hand-Collection
BT_0950_Cassidinae sp. 10	KJ677849	Podocarpus NP/RBSF, Ecuador	Bombuscaro	9544506	725125	1104	20.05.2011	Hand-Collection
BT_0951_Cassidinae sp. 11	KJ677872	Podocarpus NP/RBSF, Ecuador	Bombuscaro	9544506	725125	1104	20.05.2011	Hand-Collection
BT_0952_Alticinae sp. 269	KJ677331	Podocarpus NP/RBSF, Ecuador	Bombuscaro	9544506	725125	1104	20.05.2011	Hand-Collection
BT_0953_Galerucinae sp. 28	KJ677434	Podocarpus NP/RBSF, Ecuador	Bombuscaro	9544506	725125	1104	20.05.2011	Hand-Collection
BT_0954_Galerucinae sp. 29	KJ677520	Podocarpus NP/RBSF, Ecuador	Bombuscaro	9544506	725125	1104	20.05.2011	Hand-Collection
BT_0955_Galerucinae sp. 20	KJ677564	Podocarpus NP/RBSF, Ecuador	Bombuscaro	9544506	725125	1104	20.05.2011	Hand-Collection
BT_0957_Galerucinae sp. 36	KJ677764	Podocarpus NP/RBSF, Ecuador	Bombuscaro	9544506	725125	1104	20.05.2011	Hand-Collection
BT_0958_Alticinae sp. 154	KJ677376	Podocarpus NP/RBSF, Ecuador	Bombuscaro	9544506	725125	1104	20.05.2011	Hand-Collection
BT_0959_Galerucinae sp. 52	KJ677746	Podocarpus NP/RBSF, Ecuador	Bombuscaro	9544506	725125	1104	20.05.2011	Hand-Collection
BT_0960_Galerucinae sp. 47	KJ677639	Podocarpus NP/RBSF, Ecuador	Bombuscaro	9544506	725125	1104	20.05.2011	Hand-Collection
BT_0961_Alticinae sp. 77	KJ677489	Podocarpus NP/RBSF, Ecuador	Bombuscaro	9544506	725125	1104	20.05.2011	Hand-Collection
BT_0962_Alticinae sp. 76	KJ677343	Podocarpus NP/RBSF, Ecuador	Bombuscaro	9544506	725125	1104	20.05.2011	Hand-Collection
BT_0964_Alticinae sp. 30	KJ677339	Podocarpus NP/RBSF, Ecuador	Bombuscaro	9544506	725125	1104	20.05.2011	Hand-Collection
BT_0965_Criocerinae sp. 2	KJ677812	Podocarpus NP/RBSF, Ecuador	Bombuscaro	9544506	725125	1104	20.05.2011	Hand-Collection
BT_0966_Criocerinae sp. 3	KJ677274	Podocarpus NP/RBSF, Ecuador	Bombuscaro	9544506	725125	1104	20.05.2011	Hand-Collection
BT_0967_Eumolpinae sp. 46	KJ677920	Podocarpus NP/RBSF, Ecuador	Bombuscaro	9544506	725125	1104	20.05.2011	Hand-Collection
BT_0968_Alticinae sp. 99	KJ677440	Podocarpus NP/RBSF, Ecuador	Bombuscaro	9544506	725125	1104	20.05.2011	Hand-Collection
BT_0971_Alticinae sp. 194	KJ677341	Podocarpus NP/RBSF, Ecuador	Bombuscaro	9544506	725125	1104	20.05.2011	Hand-Collection
BT_0972_Alticinae sp. 127	KJ677276	Podocarpus NP/RBSF, Ecuador	Bombuscaro	9544506	725125	1104	20.05.2011	Hand-Collection
BT_0973_Alticinae sp. 97	KJ677285	Podocarpus NP/RBSF, Ecuador	Bombuscaro	9544506	725125	1104	20.05.2011	Hand-Collection
BT_1030_Alticinae sp. 240	KJ677332	Podocarpus NP/RBSF, Ecuador	ECSF	9560423	714258	1933	27.05.2011	Malaise Trapping
BT_1031_Alticinae sp. 2	KJ677707	Podocarpus NP/RBSF, Ecuador	ECSF	9560423	714258	1933	27.05.2011	Malaise Trapping

BT_1033_Alticinae sp. 149	KJ677719	Podocarpus NP/RBSF, Ecuador	ECSF	9560423	714258	1933	27.05.2011	Malaise Trapping
BT_1034_Alticinae sp. 48	KJ677377	Podocarpus NP/RBSF, Ecuador	ECSF	9560423	714258	1933	27.05.2011	Malaise Trapping
BT_1035_Alticinae sp. 150	KJ677721	Podocarpus NP/RBSF, Ecuador	ECSF	9560423	714258	1933	27.05.2011	Malaise Trapping
BT_1036_Alticinae sp. 62	KJ677422	Podocarpus NP/RBSF, Ecuador	ECSF	9560423	714258	1933	27.05.2011	Malaise Trapping
BT_1038_Alticinae sp. 65	KJ677478	Podocarpus NP/RBSF, Ecuador	ECSF	9560423	714258	1933	27.05.2011	Malaise Trapping
BT_1043_Alticinae sp. 66	KJ677479	Podocarpus NP/RBSF, Ecuador	ECSF	9560423	714258	1933	27.05.2011	Malaise Trapping
BT_1055_Alticinae sp. 85	KJ677393	Podocarpus NP/RBSF, Ecuador	ECSF	9560423	714258	1933	27.05.2011	Malaise Trapping
BT_1060_Alticinae sp. 85	KJ677399	Podocarpus NP/RBSF, Ecuador	ECSF	9560423	714258	1933	27.05.2011	Malaise Trapping
BT_1061_Galerucinae sp. 96	KJ677684	Podocarpus NP/RBSF, Ecuador	ECSF	9560423	714258	1933	27.05.2011	Malaise Trapping
BT_1064_Alticinae sp. 142	KJ677592	Podocarpus NP/RBSF, Ecuador	ECSF	9560423	714258	1933	27.05.2011	Malaise Trapping
BT_1065_Alticinae sp. 20	KJ677681	Podocarpus NP/RBSF, Ecuador	ECSF	9560423	714258	1933	27.05.2011	Malaise Trapping
BT_1070_Alticinae sp. 97	KJ677307	Podocarpus NP/RBSF, Ecuador	ECSF	9560423	714258	1933	27.05.2011	Malaise Trapping
BT_1071_Alticinae sp. 96	KJ677480	Podocarpus NP/RBSF, Ecuador	ECSF	9560423	714258	1933	27.05.2011	Malaise Trapping
BT_1079_Alticinae sp. 104	KJ677328	Podocarpus NP/RBSF, Ecuador	ECSF	9560455	713721	2002	07.05.2011	Sweep-netting
BT_1080_Eumolpinae sp. 74	KJ677835	Podocarpus NP/RBSF, Ecuador	ECSF	9560455	713721	2002	07.05.2011	Sweep-netting
BT_1081_Alticinae sp. 27	KJ677409	Podocarpus NP/RBSF, Ecuador	ECSF	9560455	713721	2002	07.05.2011	Beating
BT_1082_Alticinae sp. 104	KJ677319	Podocarpus NP/RBSF, Ecuador	ECSF	9560455	713721	2002	07.05.2011	Beating
BT_1083_Alticinae sp. 96	KJ677481	Podocarpus NP/RBSF, Ecuador	ECSF	9560235	713688	2039	08.05.2011	Sweep-netting
BT_1085_Alticinae sp. 61	KJ677283	Podocarpus NP/RBSF, Ecuador	ECSF	9560235	713688	2039	08.05.2011	Sweep-netting
BT_1086_Alticinae sp. 83	KJ677337	Podocarpus NP/RBSF, Ecuador	ECSF	9560235	713688	2039	08.05.2011	Sweep-netting
BT_1087_Alticinae sp. 109	KJ677670	Podocarpus NP/RBSF, Ecuador	ECSF	9560235	713688	2039	08.05.2011	Sweep-netting
BT_1088_Galerucinae sp. 31	KJ677754	Podocarpus NP/RBSF, Ecuador	ECSF	9560235	713688	2039	08.05.2011	Sweep-netting
BT_1090_Alticinae sp. 19	KJ677423	Podocarpus NP/RBSF, Ecuador	ECSF	9560235	713688	2039	08.05.2011	Sweep-netting
BT_1091_Cassidinae sp. 12	KJ677826	Podocarpus NP/RBSF, Ecuador	ECSF	9560235	713688	2039	08.05.2011	Beating
BT_1092_Cassidinae sp. 3	KJ677854	Podocarpus NP/RBSF, Ecuador	ECSF	9560235	713688	2039	08.05.2011	Beating
BT_1093_Hispinae sp. 7	KJ677869	Podocarpus NP/RBSF, Ecuador	ECSF	9560235	713688	2039	08.05.2011	Beating
BT_1094_Eumolpinae sp. 38	KJ677929	Podocarpus NP/RBSF, Ecuador	ECSF	9560235	713688	2039	08.05.2011	Beating
BT_1095_Alticinae sp. 96	KJ677464	Podocarpus NP/RBSF, Ecuador	ECSF	9560235	713688	2039	08.05.2011	Beating
BT_1096_Galerucinae sp. 36	KJ677762	Podocarpus NP/RBSF, Ecuador	ECSF	9560235	713688	2039	08.05.2011	Beating
BT_1098_Alticinae sp. 97	KJ677303	Podocarpus NP/RBSF, Ecuador	ECSF	9560235	713688	2039	08.05.2011	Hand-Coll.on Plot
BT_1104_Alticinae sp. 140	KJ677569	Podocarpus NP/RBSF, Ecuador	ECSF	9560143	713666	2089	10.05.2011	Beating
BT_1105_Alticinae sp. 74	KJ677680	Podocarpus NP/RBSF, Ecuador	ECSF	9560143	713666	2089	10.05.2011	Beating
BT_1106_Galerucinae sp. 64	KJ677793	Podocarpus NP/RBSF, Ecuador	ECSF	9560143	713666	2089	10.05.2011	Beating
BT_1107_Alticinae sp. 99	KJ677672	Podocarpus NP/RBSF, Ecuador	ECSF	9560467	714261	1954	12.05.2011	Sweep-netting
BT_1108_Alticinae sp. 92	KJ677738	Podocarpus NP/RBSF, Ecuador	ECSF	9560467	714261	1954	12.05.2011	Sweep-netting
BT_1109_Alticinae sp. 41	KJ677598	Podocarpus NP/RBSF, Ecuador	ECSF	9560467	714261	1954	12.05.2011	Sweep-netting
BT_1110_Galerucinae sp. 46	KJ677638	Podocarpus NP/RBSF, Ecuador	ECSF	9560467	714261	1954	12.05.2011	Sweep-netting
BT_1112_Alticinae sp. 112	KJ677613	Podocarpus NP/RBSF, Ecuador	ECSF	9560467	714261	1954	12.05.2011	Sweep-netting
BT_1114_Alticinae sp. 41	KJ677599	Podocarpus NP/RBSF, Ecuador	ECSF	9560467	714261	1954	12.05.2011	Beating
BT_1117_Alticinae sp. 49	KJ677609	Podocarpus NP/RBSF, Ecuador	ECSF	9560467	714261	1954	12.05.2011	Hand-Coll.on Plot
BT_1118_Alticinae sp. 123	KJ677619	Podocarpus NP/RBSF, Ecuador	ECSF	9560467	714261	1954	12.05.2011	Hand-Coll.on Plot
BT_1119_Alticinae sp. 96	KJ677482	Podocarpus NP/RBSF, Ecuador	ECSF	9560467	714261	1954	12.05.2011	Hand-Coll.on Plot
BT_1121_Alticinae sp. 124	KJ677490	Podocarpus NP/RBSF, Ecuador	ECSF	9560467	714261	1954	12.05.2011	Hand-Coll.on Plot
BT_1122_Alticinae sp. 265	KJ677430	Podocarpus NP/RBSF, Ecuador	ECSF	9560467	714261	1954	12.05.2011	Hand-Coll.on Plot
BT_1124_Cassidinae sp. 7	KJ677838	Podocarpus NP/RBSF, Ecuador	ECSF	9560423	714258	1933	12.05.2011	Sweep-netting
BT_1125_Galerucinae sp. 59	KJ677734	Podocarpus NP/RBSF, Ecuador	ECSF	9560423	714258	1933	12.05.2011	Sweep-netting
BT_1126_Hispinae sp. 3	KJ677843	Podocarpus NP/RBSF, Ecuador	ECSF	9560423	714258	1933	12.05.2011	Sweep-netting
BT_1127_Alticinae sp. 66	KJ677483	Podocarpus NP/RBSF, Ecuador	ECSF	9560423	714258	1933	12.05.2011	Sweep-netting
BT_1128_Alticinae sp. 18	KJ677280	Podocarpus NP/RBSF, Ecuador	ECSF	9560423	714258	1933	12.05.2011	Sweep-netting
BT_1129_Alticinae sp. 123	KJ677617	Podocarpus NP/RBSF, Ecuador	ECSF	9560423	714258	1933	12.05.2011	Sweep-netting
BT_1131_Alticinae sp. 81	KJ677767	Podocarpus NP/RBSF, Ecuador	ECSF	9560423	714258	1933	12.05.2011	Sweep-netting
BT_1132_Alticinae sp. 86	KJ677400	Podocarpus NP/RBSF, Ecuador	ECSF	9560423	714258	1933	12.05.2011	Sweep-netting
BT_1138_Alticinae sp. 86	KJ677394	Podocarpus NP/RBSF, Ecuador	ECSF	9560423	714258	1933	12.05.2011	Sweep-netting
BT_1142_Alticinae sp. 96	KJ677484	Podocarpus NP/RBSF, Ecuador	ECSF	9560423	714258	1933	12.05.2011	Beating
BT_1143_Hispinae sp. 7	KJ677870	Podocarpus NP/RBSF, Ecuador	ECSF	9560423	714258	1933	12.05.2011	Beating
BT_1145_Cassidinae sp. 3	KJ677855	Podocarpus NP/RBSF, Ecuador	ECSF	9560423	714258	1933	12.05.2011	Hand-Coll.on Plot
BT_1146_Eumolpinae sp. 14	KJ677934	Podocarpus NP/RBSF, Ecuador	ECSF	9560423	714258	1933	12.05.2011	Hand-Coll.on Plot
BT_1147_Alticinae sp. 152	KJ677355	Podocarpus NP/RBSF, Ecuador	ECSF	9560423	714258	1933	12.05.2011	Hand-Coll.on Plot
BT_1148_Alticinae sp. 96	KJ677485	Podocarpus NP/RBSF, Ecuador	ECSF	9560423	714258	1933	12.05.2011	Hand-Coll.on Plot
BT_1151_Alticinae sp. 57	KJ677445	Podocarpus NP/RBSF, Ecuador	Bombuscaro	9544970	725703	1066	14.05.2011	Sweep-netting
BT_1152_Alticinae sp. 141	KJ677773	Podocarpus NP/RBSF, Ecuador	Bombuscaro	9544713	725565	1020	14.05.2011	Sweep-netting
BT_1157_Alticinae sp. 149	KJ677709	Podocarpus NP/RBSF, Ecuador	Bombuscaro	9544705	725544	1026	14.05.2011	Sweep-netting
BT_1158_Alticinae sp. 146	KJ677567	Podocarpus NP/RBSF, Ecuador	Bombuscaro	9544705	725544	1026	14.05.2011	Sweep-netting
BT_1160_Alticinae sp. 55	KJ677602	Podocarpus NP/RBSF, Ecuador	Bombuscaro	9544705	725544	1026	14.05.2011	Sweep-netting
BT_1161_Alticinae sp. 143	KJ677578	Podocarpus NP/RBSF, Ecuador	Bombuscaro	9544705	725544	1026	14.05.2011	Sweep-netting
BT_1165_Alticinae sp. 61	KJ677284	Podocarpus NP/RBSF, Ecuador	ECSF	9560321	714040	2063	17.05.2011	Sweep-netting
BT_1166_Alticinae sp. 140	KJ677572	Podocarpus NP/RBSF, Ecuador	ECSF	9560321	714040	2063	17.05.2011	Sweep-netting
BT_1170_Alticinae sp. 105	KJ677316	Podocarpus NP/RBSF, Ecuador	ECSF	9560321	714040	2063	17.05.2011	Sweep-netting
BT_1171_Alticinae sp. 85	KJ677386	Podocarpus NP/RBSF, Ecuador	ECSF	9560321	714040	2063	17.05.2011	Sweep-netting
BT_1175_Alticinae sp. 87	KJ677503	Podocarpus NP/RBSF, Ecuador	ECSF	9560321	714040	2063	17.05.2011	Beating
BT_1176_Alticinae sp. 52	KJ677353	Podocarpus NP/RBSF, Ecuador	ECSF	9560321	714040	2063	17.05.2011	Beating
BT_1178_Alticinae sp. 87	KJ677504	Podocarpus NP/RBSF, Ecuador	ECSF	9560321	714040	2063	17.05.2011	Hand-Coll.on Plot
BT_1179_Alticinae sp. 118	KJ677665	Podocarpus NP/RBSF, Ecuador	ECSF	9560354	714028	2054	17.05.2011	Sweep-netting
BT_1194_Alticinae sp. 87	KJ677500	Podocarpus NP/RBSF, Ecuador	ECSF	9560354	714028	2054	17.05.2011	Beating
BT_1196_Alticinae sp. 108	KJ677455	Podocarpus NP/RBSF, Ecuador	ECSF	9560354	714028	2054	17.05.2011	Beating
BT_1197_Alticinae sp. 104	KJ677320	Podocarpus NP/RBSF, Ecuador	ECSF	9560354	714028	2054	17.05.2011	Beating
BT_1198_Alticinae sp. 131	KJ677655	Podocarpus NP/RBSF, Ecuador	ECSF	9560354	714028	2054	17.05.2011	Beating
BT_1199_Alticinae sp. 118	KJ677661	Podocarpus NP/RBSF, Ecuador	ECSF	9560354	714028	2054	17.05.2011	Hand-Coll.on Plot
BT_1205_Alticinae sp. 52	KJ677357	Podocarpus NP/RBSF, Ecuador	ECSF	9560505	714170	1913	18.05.2011	Sweep-netting
BT_1208_Galerucinae sp. 46	KJ677634	Podocarpus NP/RBSF, Ecuador	ECSF	9560505	714170	1913	18.05.2011	Sweep-netting
BT_1210_Alticinae sp. 64	KJ677452	Podocarpus NP/RBSF, Ecuador	ECSF	9560505	714170	1913	18.05.2011	Sweep-netting
BT_1211_Alticinae sp. 18	KJ677427	Podocarpus NP/RBSF, Ecuador	ECSF	9560505	714170	1913	18.05.2011	Sweep-netting
BT_1212_Alticinae sp. 17	KJ677380	Podocarpus NP/RBSF, Ecuador	ECSF	9560505	714170	1913	18.05.2011	Sweep-netting
BT_1213_Alticinae sp. 96	KJ677486	Podocarpus NP/RBSF, Ecuador	ECSF	9560505	714170	1913	18.05.2011	Sweep-netting
BT_1214_Alticinae sp. 104	KJ677649	Podocarpus NP/RBSF, Ecuador	ECSF	9560505	714170	1913	18.05.2011	Sweep-netting
BT_1215_Alticinae sp. 144	KJ677584	Podocarpus NP/RBSF, Ecuador	ECSF	9560505	714170	1913	18.05.2011	Sweep-netting

BT_1216_Alticinae sp. 49	KJ677606	Podocarpus NP/RBSF, Ecuador	ECSF	9560505	714170	1913	18.05.2011	Sweep-netting
BT_1217_Alticinae sp. 145	KJ677579	Podocarpus NP/RBSF, Ecuador	ECSF	9560505	714170	1913	18.05.2011	Sweep-netting
BT_1219_Alticinae sp. 86	KJ677406	Podocarpus NP/RBSF, Ecuador	ECSF	9560505	714170	1913	18.05.2011	Sweep-netting
BT_1220_Galerucinae sp. 34	KJ677696	Podocarpus NP/RBSF, Ecuador	ECSF	9560505	714170	1913	18.05.2011	Sweep-netting
BT_1222_Alticinae sp. 52	KJ677358	Podocarpus NP/RBSF, Ecuador	ECSF	9560505	714170	1913	18.05.2011	Beating
BT_1223_Alticinae sp. 28	KJ677348	Podocarpus NP/RBSF, Ecuador	ECSF	9560505	714170	1913	18.05.2011	Beating
BT_1224_Galerucinae sp. 75	KJ677801	Podocarpus NP/RBSF, Ecuador	ECSF	9560505	714170	1913	18.05.2011	Beating
BT_1225_Eumolpinae sp. 40	KJ677867	Podocarpus NP/RBSF, Ecuador	ECSF	9560505	714170	1913	18.05.2011	Beating
BT_1226_Galerucinae sp. 34	KJ677697	Podocarpus NP/RBSF, Ecuador	ECSF	9560505	714170	1913	18.05.2011	Beating
BT_1228_Alticinae sp. 96	KJ677487	Podocarpus NP/RBSF, Ecuador	ECSF	9560505	714170	1913	18.05.2011	Beating
BT_1230_Alticinae sp. 115	KJ677295	Podocarpus NP/RBSF, Ecuador	ECSF	9560505	714170	1913	18.05.2011	Beating
BT_1233_Alticinae sp. 52	KJ677356	Podocarpus NP/RBSF, Ecuador	ECSF	9560505	714170	1913	18.05.2011	Hand-Coll.on Plot
BT_1235_Alticinae sp. 96	KJ677465	Podocarpus NP/RBSF, Ecuador	ECSF	9560505	714170	1913	18.05.2011	Hand-Coll.on Plot
BT_1240_Eumolpinae sp. 41	KJ677857	Podocarpus NP/RBSF, Ecuador	ECSF	9560505	714170	1913	18.05.2011	Hand-Coll.on Plot
BT_1245_Alticinae sp. 85	KJ677389	Podocarpus NP/RBSF, Ecuador	ECSF	9560383	714029	2039	18.05.2011	Sweep-netting
BT_1249_Alticinae sp. 104	KJ677323	Podocarpus NP/RBSF, Ecuador	ECSF	9560383	714029	2039	18.05.2011	Beating
BT_1251_Alticinae sp. 131	KJ677656	Podocarpus NP/RBSF, Ecuador	ECSF	9560383	714029	2039	18.05.2011	Beating
BT_1252_Alticinae sp. 86	KJ677401	Podocarpus NP/RBSF, Ecuador	ECSF	9560383	714029	2039	18.05.2011	Beating
BT_1258_Galerucinae sp. 28	KJ677438	Podocarpus NP/RBSF, Ecuador	Bombuscaro	9544210	724474	1268	20.05.2011	Sweep-netting
BT_1259_Galerucinae sp. 49	KJ677643	Podocarpus NP/RBSF, Ecuador	Bombuscaro	9544210	724474	1268	20.05.2011	Sweep-netting
BT_1260_Eumolpinae sp. 24	KJ677916	Podocarpus NP/RBSF, Ecuador	Bombuscaro	9544210	724474	1268	20.05.2011	Sweep-netting
BT_1263_Alticinae sp. 51	KJ677369	Podocarpus NP/RBSF, Ecuador	Bombuscaro	9544210	724474	1268	20.05.2011	Sweep-netting
BT_1264_Alticinae sp. 78	KJ677616	Podocarpus NP/RBSF, Ecuador	Bombuscaro	9544210	724474	1268	20.05.2011	Sweep-netting
BT_1267_Alticinae sp. 128	KJ677510	Podocarpus NP/RBSF, Ecuador	Bombuscaro	9544210	724474	1268	20.05.2011	Beating
BT_1269_Alticinae sp. 9	KJ677725	Podocarpus NP/RBSF, Ecuador	Bombuscaro	9544210	724474	1268	20.05.2011	Hand-Coll.on Plot
BT_1270_Galerucinae sp. 28	KJ677435	Podocarpus NP/RBSF, Ecuador	Bombuscaro	9544210	724474	1268	20.05.2011	Hand-Coll.on Plot
BT_1271_Alticinae sp. 57	KJ677444	Podocarpus NP/RBSF, Ecuador	Bombuscaro	9544210	724474	1268	20.05.2011	Hand-Coll.on Plot
BT_1272_Alticinae sp. 128	KJ677507	Podocarpus NP/RBSF, Ecuador	Bombuscaro	9544210	724474	1268	20.05.2011	Hand-Coll.on Plot
BT_1273_Galerucinae sp. 28	KJ677436	Podocarpus NP/RBSF, Ecuador	Bombuscaro	9544174	724502	1266	20.05.2011	Sweep-netting
BT_1274_Alticinae sp. 42	KJ677410	Podocarpus NP/RBSF, Ecuador	Bombuscaro	9544174	724502	1266	20.05.2011	Sweep-netting
BT_1275_Alticinae sp. 51	KJ677363	Podocarpus NP/RBSF, Ecuador	Bombuscaro	9544174	724502	1266	20.05.2011	Sweep-netting
BT_1278_Alticinae sp. 143	KJ677576	Podocarpus NP/RBSF, Ecuador	Bombuscaro	9544174	724502	1266	20.05.2011	Sweep-netting
BT_1280_Alticinae sp. 69	KJ677461	Podocarpus NP/RBSF, Ecuador	Bombuscaro	9544174	724502	1266	20.05.2011	Sweep-netting
BT_1281_Galerucinae sp. 47	KJ677640	Podocarpus NP/RBSF, Ecuador	Bombuscaro	9544174	724502	1266	20.05.2011	Sweep-netting
BT_1282_Galerucinae sp. 52	KJ677744	Podocarpus NP/RBSF, Ecuador	Bombuscaro	9544174	724502	1266	20.05.2011	Sweep-netting
BT_1283_Galerucinae sp. 6	KJ677536	Podocarpus NP/RBSF, Ecuador	Bombuscaro	9544174	724502	1266	20.05.2011	Beating
BT_1284_Galerucinae sp. 63	KJ677529	Podocarpus NP/RBSF, Ecuador	Bombuscaro	9544174	724502	1266	20.05.2011	Beating
BT_1286_Alticinae sp. 21	KJ677441	Podocarpus NP/RBSF, Ecuador	Bombuscaro	9544174	724502	1266	20.05.2011	Beating
BT_1287_Alticinae sp. 67	KJ677458	Podocarpus NP/RBSF, Ecuador	Bombuscaro	9544174	724502	1266	20.05.2011	Beating
BT_1288_Alticinae sp. 103	KJ677364	Podocarpus NP/RBSF, Ecuador	Bombuscaro	9544174	724502	1266	20.05.2011	Beating
BT_1293_Alticinae sp. 50	KJ677383	Podocarpus NP/RBSF, Ecuador	Bombuscaro	9544174	724502	1266	20.05.2011	Beating
BT_1294_Galerucinae sp. 72	KJ677804	Podocarpus NP/RBSF, Ecuador	Bombuscaro	9544174	724502	1266	20.05.2011	Beating
BT_1295_Alticinae sp. 57	KJ677446	Podocarpus NP/RBSF, Ecuador	Bombuscaro	9544174	724502	1266	20.05.2011	Beating
BT_1304_Galerucinae sp. 47	KJ677641	Podocarpus NP/RBSF, Ecuador	Bombuscaro	9544174	724502	1266	20.05.2011	Hand-Coll.on Plot
BT_1305_Alticinae sp. 93	KJ677359	Podocarpus NP/RBSF, Ecuador	Bombuscaro	9544174	724502	1266	20.05.2011	Hand-Coll.on Plot
BT_1307_Eumolpinae sp. 16	KJ677938	Podocarpus NP/RBSF, Ecuador	Bombuscaro	9544174	724502	1266	20.05.2011	Hand-Coll.on Plot
BT_1308_Alticinae sp. 134	KJ677596	Podocarpus NP/RBSF, Ecuador	Bombuscaro	9544205	724491	1257	20.05.2011	Sweep-netting
BT_1310_Alticinae sp. 35	KJ677612	Podocarpus NP/RBSF, Ecuador	Bombuscaro	9544205	724491	1257	20.05.2011	Sweep-netting
BT_1311_Alticinae sp. 69	KJ677460	Podocarpus NP/RBSF, Ecuador	Bombuscaro	9544205	724491	1257	20.05.2011	Sweep-netting
BT_1312_Alticinae sp. 93	KJ677360	Podocarpus NP/RBSF, Ecuador	Bombuscaro	9544205	724491	1257	20.05.2011	Sweep-netting
BT_1318_Eumolpinae sp. 24	KJ677917	Podocarpus NP/RBSF, Ecuador	Bombuscaro	9544205	724491	1257	20.05.2011	Beating
BT_1319_Alticinae sp. 51	KJ677370	Podocarpus NP/RBSF, Ecuador	Bombuscaro	9544205	724491	1257	20.05.2011	Beating
BT_1321_Galerucinae sp. 61	KJ677517	Podocarpus NP/RBSF, Ecuador	ECSF	9560307	713655	1993	23.05.2011	Sweep-netting
BT_1322_Alticinae sp. 19	KJ677424	Podocarpus NP/RBSF, Ecuador	ECSF	9560307	713655	1993	23.05.2011	Sweep-netting
BT_1323_Alticinae sp. 104	KJ677651	Podocarpus NP/RBSF, Ecuador	ECSF	9560307	713655	1993	23.05.2011	Sweep-netting
BT_1324_Alticinae sp. 92	KJ677739	Podocarpus NP/RBSF, Ecuador	ECSF	9560307	713655	1993	23.05.2011	Sweep-netting
BT_1326_Eumolpinae sp. 19	KJ677880	Podocarpus NP/RBSF, Ecuador	ECSF	9560307	713655	1993	23.05.2011	Hand-Coll.on Plot
BT_1335_Alticinae sp. 118	KJ677662	Podocarpus NP/RBSF, Ecuador	Cajanuma	9545616	701966	2880	24.05.2011	Sweep-netting
BT_1340_Galerucinae sp. 66	KJ677798	Podocarpus NP/RBSF, Ecuador	Cajanuma	9545590	702017	2865	24.05.2011	Beating
BT_1349_Alticinae sp. 51	KJ677352	Podocarpus NP/RBSF, Ecuador	ECSF	9560391	713726	2026	27.05.2011	Beating
BT_1350_Alticinae sp. 131	KJ677657	Podocarpus NP/RBSF, Ecuador	ECSF	9560391	713726	2026	27.05.2011	Hand-Coll.on Plot
BT_2073_Alticinae sp. 22	KJ677340	Podocarpus NP/RBSF, Ecuador	ECSF	9560200	713660	1990	05.07.2011	Malaise Trapping
BT_2074_Galerucinae sp. 34	KJ677698	Podocarpus NP/RBSF, Ecuador	ECSF	9560200	713660	1990	05.07.2011	Malaise Trapping
BT_2076_Alticinae sp. 2	KJ677706	Podocarpus NP/RBSF, Ecuador	ECSF	9560200	713660	1990	05.07.2011	Malaise Trapping
BT_2077_Galerucinae sp. 7	KJ677542	Podocarpus NP/RBSF, Ecuador	ECSF	9560200	713660	1990	05.07.2011	Malaise Trapping
BT_2078_Alticinae sp. 265	KJ677431	Podocarpus NP/RBSF, Ecuador	ECSF	9560200	713660	1990	05.07.2011	Malaise Trapping
BT_2081_Eumolpinae sp. 42	KJ677860	Podocarpus NP/RBSF, Ecuador	ECSF	9560200	713660	1990	05.07.2011	Malaise Trapping
BT_2082_Alticinae sp. 64	KJ677448	Podocarpus NP/RBSF, Ecuador	ECSF	9560200	713660	1990	05.07.2011	Malaise Trapping
BT_2084_Alticinae sp. 32	KJ677624	Podocarpus NP/RBSF, Ecuador	ECSF	9560200	713660	1990	05.07.2011	Malaise Trapping
BT_2085_Eumolpinae sp. 42	KJ677861	Podocarpus NP/RBSF, Ecuador	ECSF	9560200	713660	1990	05.07.2011	Malaise Trapping
BT_2087_Alticinae sp. 49	KJ677608	Podocarpus NP/RBSF, Ecuador	ECSF	9560200	713660	1990	05.07.2011	Malaise Trapping
BT_2090_Alticinae sp. 81	KJ677766	Podocarpus NP/RBSF, Ecuador	ECSF	9560200	713660	1990	05.07.2011	Malaise Trapping
BT_2093_Alticinae sp. 96	KJ677466	Podocarpus NP/RBSF, Ecuador	ECSF	9560200	713660	1990	05.07.2011	Malaise Trapping
BT_2097_Alticinae sp. 122	KJ677685	Podocarpus NP/RBSF, Ecuador	ECSF	9560200	713660	1990	05.07.2011	Malaise Trapping
BT_2101_Alticinae sp. 86	KJ677402	Podocarpus NP/RBSF, Ecuador	ECSF	9560200	713660	1990	05.07.2011	Malaise Trapping
BT_2107_Alticinae sp. 85	KJ677390	Podocarpus NP/RBSF, Ecuador	ECSF	9560200	713660	1990	05.07.2011	Malaise Trapping
BT_2109_Alticinae sp. 133	KJ677587	Podocarpus NP/RBSF, Ecuador	ECSF	9560200	713660	1990	05.07.2011	Malaise Trapping
BT_2113_Alticinae sp. 142	KJ677594	Podocarpus NP/RBSF, Ecuador	ECSF	9560200	713660	1990	05.07.2011	Malaise Trapping
BT_2119_Eumolpinae sp. 16	KJ677936	Podocarpus NP/RBSF, Ecuador	ECSF	9560200	713660	1990	05.07.2011	Malaise Trapping
BT_2123_Alticinae sp. 18	KJ677281	Podocarpus NP/RBSF, Ecuador	ECSF	9560200	713660	1990	05.07.2011	Malaise Trapping
BT_2135_Alticinae sp. 122	KJ677686	Podocarpus NP/RBSF, Ecuador	ECSF	9560200	713660	1990	05.07.2011	Malaise Trapping
BT_2136_Alticinae sp. 110	KJ677673	Podocarpus NP/RBSF, Ecuador	ECSF	9560200	713660	1990	05.07.2011	Malaise Trapping
BT_2138_Alticinae sp. 115	KJ677294	Podocarpus NP/RBSF, Ecuador	ECSF	9560200	713660	1990	05.07.2011	Malaise Trapping
BT_2152_Eumolpinae sp. 38	KJ677930	Podocarpus NP/RBSF, Ecuador	ECSF	9560352	713911	2011	14.07.2011	Hand-Collection
BT_2154_Galerucinae sp. 17	KJ677554	Podocarpus NP/RBSF, Ecuador	ECSF	9560352	713911	2011	14.07.2011	Hand-Collection
BT_2155_Alticinae sp. 115	KJ677296	Podocarpus NP/RBSF, Ecuador	ECSF	9560352	713911	2011	14.07.2011	Hand-Collection

BT_2156_Cassidinae sp. 12	KJ677830	Podocarpus NP/RBSF, Ecuador	ECSF	9560352	713911	2011	14.07.2011	Hand-Collection
BT_2157_Eumolpinae sp. 24	KJ677911	Podocarpus NP/RBSF, Ecuador	ECSF	9560352	713911	2011	14.07.2011	Hand-Collection
BT_2158_Alticinae sp. 83	KJ677338	Podocarpus NP/RBSF, Ecuador	ECSF	9560352	713911	2011	14.07.2011	Hand-Collection
BT_2161_Cassidinae sp. 12	KJ677827	Podocarpus NP/RBSF, Ecuador	ECSF	9560352	713911	2011	19.07.2011	Hand-Collection
BT_2168_Galerucinae sp. 36	KJ677763	Podocarpus NP/RBSF, Ecuador	ECSF	9560352	713911	2011	19.07.2011	Hand-Collection
BT_2170_Galerucinae sp. 34	KJ677699	Podocarpus NP/RBSF, Ecuador	ECSF	9560352	713911	2011	19.07.2011	Hand-Collection
BT_2173_Eumolpinae sp. 34	KJ677882	Podocarpus NP/RBSF, Ecuador	ECSF	9560352	713911	2011	19.07.2011	Hand-Collection
BT_2176_Galerucinae sp. 43	KJ677682	Podocarpus NP/RBSF, Ecuador	Bombuscaro	9544506	725125	1104	21.07.2011	Hand-Collection
BT_2179_Alticinae sp. 11	KJ677726	Podocarpus NP/RBSF, Ecuador	Bombuscaro	9544506	725125	1104	21.07.2011	Hand-Collection
BT_2180_Alticinae sp. 75	KJ677621	Podocarpus NP/RBSF, Ecuador	Bombuscaro	9544506	725125	1104	21.07.2011	Hand-Collection
BT_2181_Alticinae sp. 251	KJ677508	Podocarpus NP/RBSF, Ecuador	Bombuscaro	9544506	725125	1104	21.07.2011	Hand-Collection
BT_2182_Galerucinae sp. 29	KJ677521	Podocarpus NP/RBSF, Ecuador	Bombuscaro	9544506	725125	1104	21.07.2011	Hand-Collection
BT_2189_Hispinae sp. 8	KJ677845	Podocarpus NP/RBSF, Ecuador	Bombuscaro	9544506	725125	1104	21.07.2011	Hand-Collection
BT_2191_Criocerinae sp. 5	KJ677820	Podocarpus NP/RBSF, Ecuador	Bombuscaro	9544506	725125	1104	21.07.2011	Hand-Collection
BT_2192_Galerucinae sp. 28	KJ677433	Podocarpus NP/RBSF, Ecuador	Bombuscaro	9544506	725125	1104	21.07.2011	Hand-Collection
BT_2193_Alticinae sp. 76	KJ677344	Podocarpus NP/RBSF, Ecuador	Bombuscaro	9544506	725125	1104	21.07.2011	Hand-Collection
BT_2194_Alticinae sp. 127	KJ677275	Podocarpus NP/RBSF, Ecuador	Bombuscaro	9544506	725125	1104	21.07.2011	Hand-Collection
BT_2196_Alticinae sp. 127	KJ677278	Podocarpus NP/RBSF, Ecuador	Bombuscaro	9544506	725125	1104	21.07.2011	Hand-Collection
BT_2197_Eumolpinae sp. 47	KJ677919	Podocarpus NP/RBSF, Ecuador	Bombuscaro	9544506	725125	1104	21.07.2011	Hand-Collection
BT_2208_Alticinae sp. 112	KJ677614	Podocarpus NP/RBSF, Ecuador	Bombuscaro	9544506	725125	1104	02.08.2011	Hand-Collection
BT_2312_Alticinae sp. 42	KJ677413	Podocarpus NP/RBSF, Ecuador	ECSF	9560455	713721	2002	04.08.2011	Malaise Trapping
BT_2491_Alticinae sp. 85	KJ677387	Podocarpus NP/RBSF, Ecuador	ECSF	9560321	714040	2063	03.06.2011	Sweep-netting
BT_2492_Alticinae sp. 104	KJ677329	Podocarpus NP/RBSF, Ecuador	ECSF	9560321	714040	2063	03.06.2011	Sweep-netting
BT_2495_Alticinae sp. 243	KJ677418	Podocarpus NP/RBSF, Ecuador	ECSF	9560321	714040	2063	03.06.2011	Beating
BT_2496_Alticinae sp. 140	KJ677573	Podocarpus NP/RBSF, Ecuador	ECSF	9560321	714040	2063	03.06.2011	Beating
BT_2498_Alticinae sp. 52	KJ677354	Podocarpus NP/RBSF, Ecuador	ECSF	9560321	714040	2063	03.06.2011	Hand-Coll.on Plot
BT_2499_Alticinae sp. 118	KJ677668	Podocarpus NP/RBSF, Ecuador	Cajanuma	9545609	702375	2805	07.06.2011	Beating
BT_2502_Alticinae sp.160	KJ677362	Podocarpus NP/RBSF, Ecuador	ECSF	9560505	714170	1913	14.06.2011	Sweep-netting
BT_2504_Galerucinae sp. 98	KJ677627	Podocarpus NP/RBSF, Ecuador	ECSF	9560505	714170	1913	14.06.2011	Sweep-netting
BT_2505_Galerucinae sp. 46	KJ677635	Podocarpus NP/RBSF, Ecuador	ECSF	9560505	714170	1913	14.06.2011	Sweep-netting
BT_2506_Alticinae sp. 47	KJ677735	Podocarpus NP/RBSF, Ecuador	ECSF	9560505	714170	1913	14.06.2011	Beating
BT_2516_Galerucinae sp. 9	KJ677530	Podocarpus NP/RBSF, Ecuador	Bombuscaro	9544713	725565	1020	21.06.2011	Beating
BT_2517_Alticinae sp. 201	KJ677333	Podocarpus NP/RBSF, Ecuador	Bombuscaro	9544713	725565	1020	21.06.2011	Hand-Coll.on Plot
BT_2518_Alticinae sp. 70	KJ677511	Podocarpus NP/RBSF, Ecuador	Bombuscaro	9544713	725565	1020	21.06.2011	Hand-Coll.on Plot
BT_2519_Alticinae sp. 153	KJ677384	Podocarpus NP/RBSF, Ecuador	Bombuscaro	9544705	725544	1026	21.06.2011	Sweep-netting
BT_2521_Galerucinae sp. 82	KJ677688	Podocarpus NP/RBSF, Ecuador	Bombuscaro	9544705	725544	1026	21.06.2011	Beating
BT_2522_Alticinae sp. 51	KJ677365	Podocarpus NP/RBSF, Ecuador	Bombuscaro	9544705	725544	1026	21.06.2011	Beating
BT_2523_Alticinae sp. 153	KJ677385	Podocarpus NP/RBSF, Ecuador	Bombuscaro	9544705	725544	1026	21.06.2011	Beating
BT_2529_Galerucinae sp. 55	KJ677745	Podocarpus NP/RBSF, Ecuador	Bombuscaro	9544734	725457	1046	21.06.2011	Sweep-netting
BT_2544_Alticinae sp. 97	KJ677312	Podocarpus NP/RBSF, Ecuador	ECSF	9560245	713695	2030	28.06.2011	Sweep-netting
BT_2546_Cassidinae sp. 4	KJ677853	Podocarpus NP/RBSF, Ecuador	ECSF	9560245	713695	2030	28.06.2011	Hand-Coll.on Plot
BT_2548_Cassidinae sp. 12	KJ677828	Podocarpus NP/RBSF, Ecuador	ECSF	9560245	713695	2030	28.06.2011	Hand-Coll.on Plot
BT_2550_Alticinae sp. 56	KJ677314	Podocarpus NP/RBSF, Ecuador	Cajanuma	9545656	702160	2885	30.06.2011	Beating
BT_2572_Alticinae sp. 18	KJ677428	Podocarpus NP/RBSF, Ecuador	ECSF	9560467	714261	1954	14.07.2011	Sweep-netting
BT_2573_Cassidinae sp. 12	KJ677829	Podocarpus NP/RBSF, Ecuador	ECSF	9560467	714261	1954	14.07.2011	Beating
BT_2575_Eumolpinae sp. 31	KJ677939	Podocarpus NP/RBSF, Ecuador	ECSF	9560467	714261	1954	14.07.2011	Beating
BT_2576_Alticinae sp. 96	KJ677467	Podocarpus NP/RBSF, Ecuador	ECSF	9560467	714261	1954	14.07.2011	Beating
BT_2578_Galerucinae sp. 34	KJ677700	Podocarpus NP/RBSF, Ecuador	ECSF	9560467	714261	1954	14.07.2011	Hand-Coll.on Plot
BT_2579_Alticinae sp. 115	KJ677299	Podocarpus NP/RBSF, Ecuador	ECSF	9560467	714261	1954	14.07.2011	Hand-Coll.on Plot
BT_2629_Eumolpinae sp. 29	KJ677903	Podocarpus NP/RBSF, Ecuador	ECSF	9560383	714029	2039	19.07.2011	Beating
BT_2631_Alticinae sp. 135	KJ677581	Podocarpus NP/RBSF, Ecuador	Bombuscaro	9544089	724773	1044	21.07.2011	Sweep-netting
BT_2632_Alticinae sp. 143	KJ677577	Podocarpus NP/RBSF, Ecuador	Bombuscaro	9544089	724773	1044	21.07.2011	Beating
BT_2637_Eumolpinae sp. 40	KJ677868	Podocarpus NP/RBSF, Ecuador	ECSF	9560307	713655	1993	26.07.2011	Sweep-netting
BT_2638_Galerucinae sp. 61	KJ677518	Podocarpus NP/RBSF, Ecuador	ECSF	9560307	713655	1993	26.07.2011	Sweep-netting
BT_2640_Alticinae sp. 104	KJ677650	Podocarpus NP/RBSF, Ecuador	ECSF	9560307	713655	1993	26.07.2011	Sweep-netting
BT_2641_Alticinae sp. 97	KJ677304	Podocarpus NP/RBSF, Ecuador	ECSF	9560307	713655	1993	26.07.2011	Sweep-netting
BT_2642_Eumolpinae sp. 19	KJ677881	Podocarpus NP/RBSF, Ecuador	ECSF	9560307	713655	1993	26.07.2011	Beating
BT_2643_Alticinae sp. 42	KJ677414	Podocarpus NP/RBSF, Ecuador	ECSF	9560307	713655	1993	26.07.2011	Beating
BT_2644_Criocerinae sp. 1	KJ677816	Podocarpus NP/RBSF, Ecuador	ECSF	9560307	713655	1993	26.07.2011	Beating
BT_2646_Alticinae sp. 96	KJ677488	Podocarpus NP/RBSF, Ecuador	ECSF	9560307	713655	1993	26.07.2011	Beating
BT_2657_Alticinae sp. 1	KJ677704	Podocarpus NP/RBSF, Ecuador	ECSF	9560235	713688	2039	26.07.2011	Sweep-netting
BT_2658_Alticinae sp. 41	KJ677600	Podocarpus NP/RBSF, Ecuador	ECSF	9560235	713688	2039	26.07.2011	Sweep-netting
BT_2659_Alticinae sp. 86	KJ677395	Podocarpus NP/RBSF, Ecuador	ECSF	9560235	713688	2039	26.07.2011	Sweep-netting
BT_2661_Hispinae sp. 9	KJ677846	Podocarpus NP/RBSF, Ecuador	ECSF	9560235	713688	2039	26.07.2011	Sweep-netting
BT_2662_Alticinae sp. 87	KJ677505	Podocarpus NP/RBSF, Ecuador	ECSF	9560235	713688	2039	26.07.2011	Beating
BT_2663_Hispinae sp. 5	KJ677841	Podocarpus NP/RBSF, Ecuador	ECSF	9560235	713688	2039	26.07.2011	Beating
BT_2665_Criocerinae sp. 4	KJ677819	Podocarpus NP/RBSF, Ecuador	ECSF	9560235	713688	2039	26.07.2011	Hand-Coll.on Plot
BT_2666_Alticinae sp. 89	KJ677736	Podocarpus NP/RBSF, Ecuador	Cajanuma	9545590	702017	2865	28.07.2011	Sweep-netting
BT_2670_Alticinae sp. 51	KJ677371	Podocarpus NP/RBSF, Ecuador	Bombuscaro	9545001	725696	1075	02.08.2011	Sweep-netting
BT_2671_Galerucinae sp. 72	KJ677806	Podocarpus NP/RBSF, Ecuador	Bombuscaro	9544970	725703	1066	02.08.2011	Sweep-netting
BT_2672_Alticinae sp. 51	KJ677372	Podocarpus NP/RBSF, Ecuador	Bombuscaro	9544970	725703	1066	02.08.2011	Sweep-netting
BT_2673_Galerucinae sp. 49	KJ677644	Podocarpus NP/RBSF, Ecuador	Bombuscaro	9544970	725703	1066	02.08.2011	Beating
BT_2697_Alticinae sp. 53	KJ677345	Podocarpus NP/RBSF, Ecuador	Cajanuma	9545631	702155	2891	11.08.2011	Sweep-netting
BT_2698_Eumolpinae sp. 71	KJ677908	Podocarpus NP/RBSF, Ecuador	Cajanuma	9545631	702155	2891	11.08.2011	Beating
BT_2705_Alticinae sp. 94	KJ677605	Podocarpus NP/RBSF, Ecuador	Cajanuma	9545630	702367	2818	11.08.2011	Sweep-netting
BT_2707_Alticinae sp. 34	KJ677626	Podocarpus NP/RBSF, Ecuador	Cajanuma	9545630	702367	2818	11.08.2011	Beating

Outgroup:

**Species**	**SequenceID/Accession Nr.**	**Source**
Anthonomus eugenii	SequenceID ARBCP010-10	BOLD
Dichromacalles dromedarius	Accession number GU987917	GenBank
Acalles camelus	Accession number GU987989	GenBank
