# Supplementary material for: Exploring the Leaf Beetle Fauna (Coleoptera: Chrysomelidae) of an Ecuadorian Mountain Forest Using DNA Barcoding
Source: PLoS One. 2016 Feb 5;11(2):e0148268. doi: 10.1371/journal.pone.0148268 (PMC4744027; doi:10.1371/journal.pone.0148268)
Supplement: S3 Table — (PDF) [file pone.0148268.s005.pdf]

Table S3. Results of species delimitation for each specimen.

Specimen	Morphospecies	Haplotype-Network	3% Dist.- Cluster	5% Dist.- Cluster	7.5% Dist.-Cluster	GMYC-Cluster	PTP-Cluster	Haplo-type
BT_0001_Eumolpinae sp. 1	Eumolpinae sp. 1	Network282	3Cluster001	5Cluster001	75Cluster001	GMYC008	PTP023	H416
BT_0002_Alticinae sp. 42	Alticinae sp. 42	Network52	3Cluster002	5Cluster002	75Cluster002	GMYC219	PTP114	H085
BT_0004_Eumolpinae sp. 42	Eumolpinae sp. 42	Network257	3Cluster003	5Cluster003	75Cluster003	GMYC001	PTP003	H388
BT_0005_Galerucinae sp. 40	Galerucinae sp. 40	Network216	3Cluster004	5Cluster004	75Cluster004	GMYC100	PTP238	H329
BT_0007_Galerucinae sp. 38	Galerucinae sp. 38	Network98	3Cluster005	5Cluster005	75Cluster005	GMYC117	PTP272	H154
BT_0008_Alticinae sp. 243	Alticinae sp. 243	Network54	3Cluster006	5Cluster006	75Cluster006	GMYC220	PTP115	H088
BT_0012_Eumolpinae sp. 21	Eumolpinae sp. 21	Network270	3Cluster007	5Cluster007	75Cluster007	GMYC022	PTP007	H401
BT_0015_Galerucinae sp. 76	Galerucinae sp. 76	Network118	3Cluster008	5Cluster008	75Cluster008	GMYC113	PTP288	H185
BT_0017_Alticinae sp. 43	Alticinae sp. 43	Network49	3Cluster009	5Cluster009	75Cluster009	GMYC227	PTP122	H081
BT_0021_Alticinae sp. 7	Alticinae sp. 7	Network187	3Cluster010	5Cluster010	75Cluster010	GMYC087	PTP204	H285
BT_0022_Alticinae sp. 219	Alticinae sp. 219	Network190	3Cluster115	5Cluster011	75Cluster011	GMYC089	PTP202	H288
BT_0024_Galerucinae sp. 1	Galerucinae sp. 1	Network112	3Cluster011	5Cluster012	75Cluster012	GMYC138	PTP264	H176
BT_0033_Galerucinae sp. 37	Galerucinae sp. 37	Network116	3Cluster012	5Cluster013	75Cluster013	GMYC115	PTP286	H181
BT_0034_Eumolpinae sp. 14	Eumolpinae sp. 14	Network285	3Cluster013	5Cluster014	75Cluster014	GMYC009	PTP016	H419
BT_0035_Eumolpinae sp. 006	Eumolpinae sp. 006	Network275	3Cluster014	5Cluster015	75Cluster015	GMYC017	PTP025	H407
BT_0036_Galerucinae sp. 11	Galerucinae sp. 11	Network103	3Cluster015	5Cluster016	75Cluster016	GMYC143	PTP258	H160
BT_0043_Galerucinae sp. 5	Galerucinae sp. 5	Network110	3Cluster016	5Cluster017	75Cluster017	GMYC137	PTP265	H172
BT_0046_Alticinae sp. 243	Alticinae sp. 243	Network53	3Cluster006	5Cluster006	75Cluster006	GMYC221	PTP116	H087
BT_0047_Alticinae sp. 42	Alticinae sp. 42	Network52	3Cluster002	5Cluster002	75Cluster002	GMYC219	PTP114	H086
BT_0048_Galerucinae sp. 39	Galerucinae sp. 39	Network97	3Cluster017	5Cluster018	75Cluster018	GMYC118	PTP271	H153
BT_0049_Galerucinae sp. 41	Galerucinae sp. 41	Network217	3Cluster018	5Cluster019	75Cluster019	GMYC101	PTP237	H330
BT_0088_Galerucinae sp. 7	Galerucinae sp. 7	Network109	3Cluster019	5Cluster020	75Cluster020	GMYC134	PTP267	H170
BT_0089_Eumolpinae sp. 1	Eumolpinae sp. 1	Network282	3Cluster001	5Cluster001	75Cluster001	GMYC008	PTP023	H416
BT_0090_Galerucinae sp. 76	Galerucinae sp. 76	Network118	3Cluster008	5Cluster008	75Cluster008	GMYC113	PTP288	H184
BT_0091_Eumolpinae sp. 1	Eumolpinae sp. 1	Network282	3Cluster001	5Cluster001	75Cluster001	GMYC008	PTP023	H416
BT_0094_Galerucinae sp. 11	Galerucinae sp. 11	Network103	3Cluster015	5Cluster016	75Cluster016	GMYC143	PTP258	H161
BT_0095_Cassidinae sp. 1	Cassidinae sp. 1	Network261	3Cluster020	5Cluster021	75Cluster021	GMYC280	PTP038	H392
BT_0096_Alticinae sp. 10	Alticinae sp. 10	Network195	3Cluster021	5Cluster022	75Cluster022	GMYC097	PTP195	H303
BT_0098_Galerucinae sp. 002	Galerucinae sp. 002	Network111	3Cluster022	5Cluster023	75Cluster023	GMYC139	PTP263	H173
BT_0099_Galerucinae sp. 7	Galerucinae sp. 7	Network92	3Cluster023	5Cluster024	75Cluster024	GMYC126	PTP284	H147
BT_0102_Alticinae sp. 44	Alticinae sp. 44	Network42	3Cluster024	5Cluster025	75Cluster025	GMYC214	PTP102	H060
BT_0103_Eumolpinae sp. 38	Eumolpinae sp. 38	Network284	3Cluster025	5Cluster026	75Cluster026	GMYC010	PTP017	H418
BT_0107_Galerucinae sp. 46	Galerucinae sp. 46	Network155	3Cluster026	5Cluster027	75Cluster027	GMYC178	PTP249	H241
BT_0109_Alticinae sp. 251	Alticinae sp. 251	Network81	3Cluster027	5Cluster028	75Cluster028	GMYC057	PTP156	H124
BT_0110_Alticinae sp. 87	Alticinae sp. 87	Network88	3Cluster028	5Cluster029	75Cluster029	GMYC061	PTP164	H136
BT_0114_Galerucinae sp. 62	Galerucinae sp. 62	Network205	3Cluster029	5Cluster030	75Cluster030	GMYC111	PTP239	H317
BT_0115_Alticinae sp. 98	Alticinae sp. 98	Network10	3Cluster030	5Cluster031	75Cluster031	GMYC253	PTP060	H010
BT_0118_Eumolpinae sp. 19	Eumolpinae sp. 19	Network265	3Cluster031	5Cluster032	75Cluster032	GMYC028	PTP004	H396
BT_0119_Alticinae sp. 124	Alticinae sp. 124	Network87	3Cluster032	5Cluster033	75Cluster033	GMYC048	PTP166	H135
BT_0121_Alticinae sp. 107	Alticinae sp. 107	Network218	3Cluster033	5Cluster034	75Cluster034	GMYC062	PTP141	H331
BT_0123_Alticinae sp. 129	Alticinae sp. 129	Network212	3Cluster034	5Cluster035	75Cluster035	GMYC065	PTP098	H325
BT_0125_Alticinae sp. 97	Alticinae sp. 97	Network13	3Cluster035	5Cluster036	75Cluster036	GMYC258	PTP065	H020
BT_0126_Alticinae sp. 123	Alticinae sp. 123	Network148	3Cluster036	5Cluster037	75Cluster037	GMYC070	PTP144	H228
BT_0130_Galerucinae sp. 34	Galerucinae sp. 34	Network183	3Cluster037	5Cluster038	75Cluster038	GMYC103	PTP232	H280
BT_0134_Galerucinae sp. 7	Galerucinae sp. 7	Network92	3Cluster023	5Cluster024	75Cluster024	GMYC126	PTP284	H148
BT_0135_Eumolpinae sp. 19	Eumolpinae sp. 19	Network265	3Cluster031	5Cluster032	75Cluster032	GMYC028	PTP004	H396
BT_0137_Cassidinae sp. 4	Cassidinae sp. 4	Network253	3Cluster038	5Cluster039	75Cluster039	GMYC284	PTP053	H379
BT_0139_Alticinae sp. 10	Alticinae sp. 10	Network195	3Cluster021	5Cluster022	75Cluster022	GMYC097	PTP195	H304
BT_0140_Alticinae sp. 28	Alticinae sp. 28	Network29	3Cluster039	5Cluster040	75Cluster040	GMYC209	PTP129	H041
BT_0144_Eumolpinae sp. 38	Eumolpinae sp. 38	Network283	3Cluster040	5Cluster041	75Cluster041	GMYC011	PTP018	H417
BT_0145_Galerucinae sp. 61	Galerucinae sp. 61	Network93	3Cluster041	5Cluster042	75Cluster042	GMYC131	PTP279	H149
BT_0146_Alticinae sp. 29	Alticinae sp. 29	Network68	3Cluster042	5Cluster043	75Cluster043	GMYC049	PTP168	H107
BT_0147_Alticinae sp. 62	Alticinae sp. 62	Network57	3Cluster043	5Cluster044	75Cluster044	GMYC224	PTP117	H092
BT_0148_Alticinae sp. 66	Alticinae sp. 66	Network85	3Cluster044	5Cluster045	75Cluster045	GMYC051	PTP169	H131
BT_0149_Alticinae sp. 249	Alticinae sp. 249	Network79	3Cluster045	5Cluster046	75Cluster046	GMYC037	PTP147	H121
BT_0153_Alticinae sp. 109	Alticinae sp. 109	Network171	3Cluster046	5Cluster047	75Cluster047	GMYC254	PTP062	H264
BT_0154_Alticinae sp. 115	Alticinae sp. 115	Network10	3Cluster030	5Cluster031	75Cluster031	GMYC253	PTP060	H010
BT_0155_Alticinae sp. 193	Alticinae sp. 193	Network172	3Cluster047	5Cluster048	75Cluster048	GMYC243	PTP206	H266
BT_0157_Alticinae sp. 97	Alticinae sp. 97	Network11	3Cluster048	5Cluster049	75Cluster049	GMYC256	PTP064	H015
BT_0158_Eumolpinae sp. 2	Eumolpinae sp. 2	Network285	3Cluster013	5Cluster014	75Cluster014	GMYC009	PTP016	H419
BT_0159_Galerucinae sp. 096	Galerucinae sp. 096	Network180	3Cluster049	5Cluster050	75Cluster050	GMYC174	PTP252	H277
BT_0174_Galerucinae sp. 46	Galerucinae sp. 46	Network155	3Cluster026	5Cluster027	75Cluster027	GMYC178	PTP249	H241
BT_0176_Galerucinae sp. 46	Galerucinae sp. 46	Network156	3Cluster050	5Cluster051	75Cluster051	GMYC179	PTP250	H242
BT_0183_Galerucinae sp. 34	Galerucinae sp. 34	Network183	3Cluster037	5Cluster038	75Cluster038	GMYC103	PTP232	H280
BT_0188_Galerucinae sp. 11	Galerucinae sp. 11	Network103	3Cluster015	5Cluster016	75Cluster016	GMYC143	PTP258	H162

BT_0189_Alticinae sp.161	Alticinae sp.161	Network38	3Cluster051	5Cluster052	75Cluster052	GMYC151	PTP071	H053
BT_0190_Eumolpinae sp. 1	Eumolpinae sp. 1	Network282	3Cluster001	5Cluster001	75Cluster001	GMYC008	PTP023	H416
BT_0196_Galerucinae sp. 10	Galerucinae sp. 10	Network232	3Cluster052	5Cluster053	75Cluster053	GMYC076	PTP228	H353
BT_0199_Alticinae sp. 118	Alticinae sp. 118	Network170	3Cluster053	5Cluster054	75Cluster054	GMYC187	PTP108	H262
BT_0201_Chrysomelinae sp. 2	Chrysomelinae sp. 2	Network206	3Cluster054	5Cluster055	75Cluster055	GMYC102	PTP236	H318
BT_0202_Galerucinae sp. 32	Galerucinae sp. 32	Network2	3Cluster055	5Cluster056	75Cluster056	GMYC175	PTP247	H002
BT_0204_Hispinae sp. 2	Hispinae sp. 2	Network255	3Cluster056	5Cluster057	75Cluster057	GMYC273	PTP051	H382
BT_0207_Galerucinae sp. 69	Galerucinae sp. 69	Network219	3Cluster057	5Cluster058	75Cluster058	GMYC086	PTP215	H333
BT_0208_Eumolpinae sp. 19	Eumolpinae sp. 19	Network265	3Cluster031	5Cluster032	75Cluster032	GMYC028	PTP004	H396
BT_0209_Cassidinae sp. 5	Cassidinae sp. 5	Network242	3Cluster058	5Cluster059	75Cluster059	GMYC277	PTP043	H364
BT_0211_Alticinae sp. 87	Alticinae sp. 87	Network88	3Cluster028	5Cluster029	75Cluster029	GMYC061	PTP164	H136
BT_0212_Galerucinae sp. 66	Galerucinae sp. 66	Network223	3Cluster059	5Cluster060	75Cluster060	GMYC079	PTP222	H341
BT_0213_Galerucinae sp. 24	Galerucinae sp. 24	Network197	3Cluster060	5Cluster061	75Cluster061	GMYC108	PTP230	H306
BT_0214_Alticinae sp. 28	Alticinae sp. 28	Network29	3Cluster039	5Cluster040	75Cluster040	GMYC209	PTP129	H043
BT_0218_Galerucinae sp. 31	Galerucinae sp. 31	Network203	3Cluster061	5Cluster062	75Cluster062	GMYC084	PTP217	H313
BT_0219_Eumolpinae sp. 073	Eumolpinae sp. 073	Network244	3Cluster062	5Cluster063	75Cluster063	GMYC031	PTP002	H368
BT_0220_Alticinae sp. 115	Alticinae sp. 115	Network10	3Cluster030	5Cluster031	75Cluster031	GMYC253	PTP060	H010
BT_0221_Eumolpinae sp. 10	Eumolpinae sp. 10	Network274	3Cluster063	5Cluster064	75Cluster064	GMYC021	PTP008	H406
BT_0223_Galerucinae sp. 34	Galerucinae sp. 34	Network183	3Cluster037	5Cluster038	75Cluster038	GMYC103	PTP232	H280
BT_0227_Cassidinae sp. 14	Cassidinae sp. 14	Network262	3Cluster064	5Cluster065	75Cluster065	GMYC281	PTP039	H393
BT_0228_Cassidinae sp. 7	Cassidinae sp. 7	Network245	3Cluster065	5Cluster066	75Cluster066	GMYC276	PTP042	H370
BT_0231_Hispinae sp. 3	Hispinae sp. 3	Network247	3Cluster066	5Cluster067	75Cluster067	GMYC272	PTP050	H372
BT_0232_Hispinae sp. 4	Hispinae sp. 4	Network1	3Cluster067	5Cluster068	75Cluster068	GMYC288	PTP033	H001
BT_0233_Alticinae sp. 61	Alticinae sp. 61	Network8	3Cluster068	5Cluster069	75Cluster069	GMYC259	PTP066	H008
BT_0234_Alticinae sp. 97	Alticinae sp. 97	Network13	3Cluster035	5Cluster036	75Cluster036	GMYC258	PTP065	H019
BT_0235_Alticinae sp. 10	Alticinae sp. 10	Network195	3Cluster021	5Cluster022	75Cluster022	GMYC097	PTP195	H302
BT_0236_Alticinae sp. 156	Alticinae sp. 156	Network194	3Cluster069	5Cluster070	75Cluster022	GMYC098	PTP194	H301
BT_0239_Galerucinae sp. 2	Galerucinae sp. 2	Network112	3Cluster011	5Cluster012	75Cluster012	GMYC138	PTP264	H174
BT_0240_Eumolpinae sp. 21	Eumolpinae sp. 21	Network270	3Cluster007	5Cluster007	75Cluster007	GMYC022	PTP007	H402
BT_0243_Alticinae sp. 118	Alticinae sp. 118	Network170	3Cluster053	5Cluster054	75Cluster054	GMYC187	PTP108	H261
BT_0244_Alticinae sp. 97	Alticinae sp. 97	Network13	3Cluster035	5Cluster036	75Cluster036	GMYC258	PTP065	H019
BT_0245_Galerucinae sp. 34	Galerucinae sp. 34	Network183	3Cluster037	5Cluster038	75Cluster038	GMYC103	PTP232	H280
BT_0246_Galerucinae sp. 30	Galerucinae sp. 30	Network184	3Cluster070	5Cluster071	75Cluster070	GMYC110	PTP240	H282
BT_0247_Criocerinae sp. 1	Criocerinae sp. 1	Network237	3Cluster071	5Cluster072	75Cluster071	GMYC264	PTP057	H358
BT_0249_Criocerinae sp. 1	Criocerinae sp. 1	Network237	3Cluster071	5Cluster072	75Cluster071	GMYC264	PTP057	H358
BT_0252_Galerucinae sp. 34	Galerucinae sp. 34	Network183	3Cluster037	5Cluster038	75Cluster038	GMYC103	PTP232	H280
BT_0254_Eumolpinae sp. 23	Eumolpinae sp. 23	Network270	3Cluster007	5Cluster007	75Cluster007	GMYC022	PTP007	H402
BT_0256_Alticinae sp. 31	Alticinae sp. 31	Network151	3Cluster072	5Cluster073	75Cluster072	GMYC241	PTP083	H234
BT_0257_Galerucinae sp. 1	Galerucinae sp. 1	Network112	3Cluster011	5Cluster012	75Cluster012	GMYC138	PTP264	H177
BT_0258_Galerucinae sp. 5	Galerucinae sp. 5	Network110	3Cluster016	5Cluster017	75Cluster017	GMYC137	PTP265	H172
BT_0259_Alticinae sp. 96	Alticinae sp. 96	Network85	3Cluster044	5Cluster045	75Cluster045	GMYC051	PTP169	H131
BT_0267_Alticinae sp. 29	Alticinae sp. 29	Network68	3Cluster042	5Cluster043	75Cluster043	GMYC049	PTP168	H108
BT_0268_Alticinae sp. 158	Alticinae sp. 158	Network127	3Cluster073	5Cluster074	75Cluster073	GMYC165	PTP179	H200
BT_0269_Alticinae sp. 86	Alticinae sp. 86	Network48	3Cluster074	5Cluster075	75Cluster074	GMYC216	PTP105	H076
BT_0271_Alticinae sp. 64	Alticinae sp. 64	Network71	3Cluster075	5Cluster076	75Cluster075	GMYC044	PTP153	H112
BT_0273_Alticinae sp. 141	Alticinae sp. 141	Network130	3Cluster076	5Cluster077	75Cluster076	GMYC170	PTP192	H203
BT_0276_Alticinae sp. 122	Alticinae sp. 122	Network218	3Cluster033	5Cluster034	75Cluster034	GMYC062	PTP141	H332
BT_0278_Alticinae sp. 124	Alticinae sp. 124	Network87	3Cluster032	5Cluster033	75Cluster033	GMYC048	PTP166	H133
BT_0279_Alticinae sp. 115	Alticinae sp. 115	Network10	3Cluster030	5Cluster031	75Cluster031	GMYC253	PTP060	H010
BT_0283_Eumolpinae sp. 20	Eumolpinae sp. 20	Network289	3Cluster077	5Cluster078	75Cluster077	GMYC003	PTP010	H425
BT_0284_Alticinae sp. 87	Alticinae sp. 87	Network88	3Cluster028	5Cluster029	75Cluster029	GMYC061	PTP164	H137
BT_0285_Galerucinae sp. 22	Galerucinae sp. 22	Network117	3Cluster078	5Cluster008	75Cluster008	GMYC114	PTP287	H182
BT_0288_Eumolpinae sp. 17	Eumolpinae sp. 17	Network277	3Cluster079	5Cluster079	75Cluster078	GMYC018	PTP027	H409
BT_0289_Eumolpinae sp. 1	Eumolpinae sp. 1	Network282	3Cluster001	5Cluster001	75Cluster001	GMYC008	PTP023	H416
BT_0292_Galerucinae sp. 76	Galerucinae sp. 76	Network118	3Cluster008	5Cluster008	75Cluster008	GMYC113	PTP288	H183
BT_0295_Galerucinae sp. 31	Galerucinae sp. 31	Network203	3Cluster061	5Cluster062	75Cluster062	GMYC084	PTP217	H314
BT_0296_Alticinae sp. 5	Alticinae sp. 5	Network162	3Cluster080	5Cluster080	75Cluster079	GMYC092	PTP205	H252
BT_0297_Alticinae sp. 142	Alticinae sp. 142	Network132	3Cluster081	5Cluster081	75Cluster080	GMYC167	PTP189	H205
BT_0298_Alticinae sp. 63	Alticinae sp. 63	Network26	3Cluster082	5Cluster082	75Cluster081	GMYC201	PTP131	H038
BT_0301_Alticinae sp. 83	Alticinae sp. 83	Network22	3Cluster083	5Cluster083	75Cluster082	GMYC230	PTP072	H033
BT_0302_Alticinae sp. 86	Alticinae sp. 86	Network48	3Cluster074	5Cluster075	75Cluster074	GMYC216	PTP105	H078
BT_0305_Alticinae sp. 81	Alticinae sp. 81	Network209	3Cluster084	5Cluster084	75Cluster083	GMYC069	PTP143	H322
BT_0307_Alticinae sp. 115	Alticinae sp. 115	Network10	3Cluster030	5Cluster031	75Cluster031	GMYC253	PTP060	H012
BT_0309_Alticinae sp. 256	Alticinae sp. 256	Network12	3Cluster085	5Cluster085	75Cluster084	GMYC257	PTP063	H016
BT_0311_Eumolpinae sp. 42	Eumolpinae sp. 42	Network257	3Cluster003	5Cluster003	75Cluster003	GMYC001	PTP003	H388
BT_0312_Eumolpinae sp. 21	Eumolpinae sp. 21	Network270	3Cluster007	5Cluster007	75Cluster007	GMYC022	PTP007	H402
BT_0314_Eumolpinae sp. 43	Eumolpinae sp. 43	Network234	3Cluster086	5Cluster086	75Cluster085	GMYC020	PTP009	H355
BT_0336_Galerucinae sp. 56	Galerucinae sp. 56	Network201	3Cluster087	5Cluster087	75Cluster086	GMYC107	PTP231	H310
BT_0337_Galerucinae sp. 56	Galerucinae sp. 56	Network201	3Cluster087	5Cluster087	75Cluster086	GMYC107	PTP231	H310

BT_0338_Galerucinae sp. 55	Galerucinae sp. 55	Network201	3Cluster087	5Cluster087	75Cluster086	GMYC107	PTP231	H310
BT_0339_Alticinae sp. 71	Alticinae sp. 71	Network18	3Cluster088	5Cluster088	75Cluster087	GMYC195	PTP112	H029
BT_0343_Galerucinae sp. 52	Galerucinae sp. 52	Network201	3Cluster087	5Cluster087	75Cluster086	GMYC107	PTP231	H310
BT_0346_Alticinae sp. 51	Alticinae sp. 51	Network41	3Cluster089	5Cluster089	75Cluster088	GMYC197	PTP126	H058
BT_0349_Eumolpinae sp. 20	Eumolpinae sp. 20	Network288	3Cluster090	5Cluster090	75Cluster089	GMYC004	PTP011	H424
BT_0352_Alticinae sp. 130	Alticinae sp. 130	Network213	3Cluster091	5Cluster091	75Cluster090	GMYC063	PTP139	H326
BT_0353_Alticinae sp. 132	Alticinae sp. 132	Network196	3Cluster092	5Cluster092	75Cluster091	GMYC212	PTP101	H305
BT_0356_Alticinae sp. 128	Alticinae sp. 128	Network91	3Cluster093	5Cluster093	75Cluster092	GMYC055	PTP160	H144
BT_0361_Alticinae sp. 50	Alticinae sp. 50	Network45	3Cluster094	5Cluster094	75Cluster093	GMYC211	PTP107	H066
BT_0363_Alticinae sp. 51	Alticinae sp. 51	Network41	3Cluster089	5Cluster089	75Cluster088	GMYC197	PTP126	H059
BT_0365_Galerucinae sp. 49	Galerucinae sp. 49	Network160	3Cluster095	5Cluster095	75Cluster094	GMYC183	PTP246	H247
BT_0372_Alticinae sp. 9	Alticinae sp. 9	Network192	3Cluster096	5Cluster096	75Cluster095	GMYC099	PTP196	H297
BT_0375_Eumolpinae sp. 32	Eumolpinae sp. 32	Network272	3Cluster097	5Cluster097	75Cluster096	GMYC007	PTP014	H404
BT_0376_Alticinae sp. 51	Alticinae sp. 51	Network41	3Cluster089	5Cluster089	75Cluster088	GMYC197	PTP126	H058
BT_0377_Eumolpinae sp. 24	Eumolpinae sp. 24	Network279	3Cluster098	5Cluster098	75Cluster097	GMYC014	PTP021	H412
BT_0380_Eumolpinae sp. 24	Eumolpinae sp. 24	Network279	3Cluster098	5Cluster098	75Cluster097	GMYC014	PTP021	H413
BT_0382_Galerucinae sp. 13	Galerucinae sp. 13	Network105	3Cluster099	5Cluster099	75Cluster098	GMYC144	PTP259	H164
BT_0383_Alticinae sp. 87	Alticinae sp. 87	Network88	3Cluster028	5Cluster029	75Cluster029	GMYC061	PTP164	H138
BT_0384_Eumolpinae sp. 39	Eumolpinae sp. 39	Network267	3Cluster100	5Cluster100	75Cluster099	GMYC025	PTP028	H398
BT_0387_Alticinae sp. 104	Alticinae sp. 104	Network17	3Cluster101	5Cluster101	75Cluster100	GMYC235	PTP080	H027
BT_0390_Alticinae sp. 90	Alticinae sp. 90	Network76	3Cluster102	5Cluster102	75Cluster101	GMYC041	PTP149	H118
BT_0391_Alticinae sp. 90	Alticinae sp. 90	Network77	3Cluster103	5Cluster102	75Cluster101	GMYC040	PTP150	H119
BT_0392_Eumolpinae sp. 074	Eumolpinae sp. 074	Network244	3Cluster062	5Cluster063	75Cluster063	GMYC031	PTP002	H368
BT_0395_Eumolpinae sp. 074	Eumolpinae sp. 074	Network244	3Cluster062	5Cluster063	75Cluster063	GMYC031	PTP002	H368
BT_0396_Cassidinae sp. 4	Cassidinae sp. 4	Network253	3Cluster038	5Cluster039	75Cluster039	GMYC284	PTP053	H379
BT_0397_Alticinae sp. 131	Alticinae sp. 131	Network164	3Cluster104	5Cluster103	75Cluster102	GMYC237	PTP077	H254
BT_0399_Galerucinae sp. 28	Galerucinae sp. 28	Network64	3Cluster105	5Cluster104	75Cluster103	GMYC128	PTP283	H102
BT_0402_Galerucinae sp. 44	Galerucinae sp. 44	Network139	3Cluster106	5Cluster105	75Cluster104	GMYC176	PTP248	H214
BT_0403_Galerucinae sp. 44	Galerucinae sp. 44	Network139	3Cluster106	5Cluster105	75Cluster104	GMYC176	PTP248	H215
BT_0405_Galerucinae sp. 45	Galerucinae sp. 45	Network154	3Cluster107	5Cluster106	75Cluster105	GMYC177	PTP251	H239
BT_0407_Galerucinae sp. 049	Galerucinae sp. 049	Network158	3Cluster108	5Cluster107	75Cluster106	GMYC182	PTP244	H244
BT_0408_Eumolpinae sp. 24	Eumolpinae sp. 24	Network279	3Cluster098	5Cluster098	75Cluster097	GMYC014	PTP021	H412
BT_0409_Eumolpinae sp. 24	Eumolpinae sp. 24	Network279	3Cluster098	5Cluster098	75Cluster097	GMYC014	PTP021	H412
BT_0410_Alticinae sp. 141	Alticinae sp. 141	Network214	3Cluster109	5Cluster108	75Cluster107	GMYC171	PTP182	H327
BT_0411_Alticinae sp. 58	Alticinae sp. 58	Network30	3Cluster110	5Cluster109	75Cluster108	GMYC248	PTP094	H044
BT_0415_Alticinae sp. 242	Alticinae sp. 242	Network143	3Cluster111	5Cluster110	75Cluster109	GMYC033	PTP086	H221
BT_0417_Galerucinae sp. 74	Galerucinae sp. 74	Network226	3Cluster112	5Cluster111	75Cluster110	GMYC083	PTP218	H345
BT_0420_Alticinae sp. 128	Alticinae sp. 128	Network89	3Cluster113	5Cluster112	75Cluster111	GMYC054	PTP163	H141
BT_0423_Eumolpinae sp. 24	Eumolpinae sp. 24	Network279	3Cluster098	5Cluster098	75Cluster097	GMYC014	PTP021	H412
BT_0425_Eumolpinae sp. 7	Eumolpinae sp. 7	Network264	3Cluster114	5Cluster113	75Cluster112	GMYC012	PTP019	H395
BT_0426_Alticinae sp. 150	Alticinae sp. 150	Network191	3Cluster115	5Cluster011	75Cluster011	GMYC088	PTP203	H291
BT_0427_Alticinae sp. 104	Alticinae sp. 104	Network17	3Cluster101	5Cluster101	75Cluster100	GMYC235	PTP080	H026
BT_0428_Alticinae sp. 105	Alticinae sp. 105	Network16	3Cluster116	5Cluster114	75Cluster113	GMYC234	PTP079	H024
BT_0429_Eumolpinae sp. 39	Eumolpinae sp. 39	Network267	3Cluster100	5Cluster100	75Cluster099	GMYC025	PTP028	H398
BT_0432_Eumolpinae sp. 30	Eumolpinae sp. 30	Network271	3Cluster117	5Cluster115	75Cluster114	GMYC019	PTP024	H403
BT_0433_Galerucinae sp. 69	Galerucinae sp. 69	Network219	3Cluster057	5Cluster058	75Cluster058	GMYC086	PTP215	H335
BT_0434_Galerucinae sp. 72	Galerucinae sp. 72	Network231	3Cluster118	5Cluster116	75Cluster115	GMYC073	PTP225	H351
BT_0436_Alticinae sp. 149	Alticinae sp. 149	Network188	3Cluster119	5Cluster117	75Cluster116	GMYC091	PTP200	H286
BT_0438_Galerucinae sp. 15	Galerucinae sp. 15	Network202	3Cluster120	5Cluster118	75Cluster117	GMYC105	PTP234	H311
BT_0441_Galerucinae sp. 082	Galerucinae sp. 082	Network182	3Cluster121	5Cluster119	75Cluster118	GMYC106	PTP235	H279
BT_0442_Galerucinae sp. 049	Galerucinae sp. 049	Network160	3Cluster095	5Cluster095	75Cluster094	GMYC183	PTP246	H247
BT_0443_Alticinae sp. 51	Alticinae sp. 51	Network41	3Cluster089	5Cluster089	75Cluster088	GMYC197	PTP126	H058
BT_0444_Alticinae sp. 143	Alticinae sp. 143	Network123	3Cluster122	5Cluster120	75Cluster119	GMYC158	PTP186	H193
BT_0447_Galerucinae sp. 7	Galerucinae sp. 7	Network109	3Cluster019	5Cluster020	75Cluster020	GMYC134	PTP267	H168
BT_0448_Galerucinae sp. 049	Galerucinae sp. 049	Network160	3Cluster095	5Cluster095	75Cluster094	GMYC183	PTP246	H247
BT_0449_Galerucinae sp. 049	Galerucinae sp. 049	Network160	3Cluster095	5Cluster095	75Cluster094	GMYC183	PTP246	H248
BT_0451_Galerucinae sp. 15	Galerucinae sp. 15	Network202	3Cluster120	5Cluster118	75Cluster117	GMYC105	PTP234	H311
BT_0452_Alticinae sp. 9	Alticinae sp. 9	Network192	3Cluster096	5Cluster096	75Cluster095	GMYC099	PTP196	H296
BT_0454_Alticinae sp. 26	Alticinae sp. 26	Network50	3Cluster123	5Cluster121	75Cluster120	GMYC213	PTP103	H082
BT_0457_Alticinae sp. 181	Alticinae sp. 181	Network5	3Cluster124	5Cluster122	75Cluster121	GMYC193	PTP212	H005
BT_0459_Galerucinae sp. 50	Galerucinae sp. 50	Network61	3Cluster125	5Cluster123	75Cluster122	GMYC127	PTP280	H097
BT_0460_Galerucinae sp. 45	Galerucinae sp. 45	Network154	3Cluster107	5Cluster106	75Cluster105	GMYC177	PTP251	H238
BT_0461_Galerucinae sp. 45	Galerucinae sp. 45	Network154	3Cluster107	5Cluster106	75Cluster105	GMYC177	PTP251	H237
BT_0462_Eumolpinae sp. 42	Eumolpinae sp. 42	Network257	3Cluster003	5Cluster003	75Cluster003	GMYC001	PTP003	H384
BT_0463_Galerucinae sp. 70	Galerucinae sp. 70	Network229	3Cluster126	5Cluster124	75Cluster123	GMYC081	PTP229	H349
BT_0465_Alticinae sp. 050	Alticinae sp. 050	Network45	3Cluster094	5Cluster094	75Cluster093	GMYC211	PTP107	H066
BT_0468_Alticinae sp. 72	Alticinae sp. 72	Network149	3Cluster127	5Cluster125	75Cluster124	GMYC240	PTP082	H230
BT_0469_Galerucinae sp. 45	Galerucinae sp. 45	Network154	3Cluster107	5Cluster106	75Cluster105	GMYC177	PTP251	H240
BT_0473_Galerucinae sp. 69	Galerucinae sp. 69	Network219	3Cluster057	5Cluster058	75Cluster058	GMYC086	PTP215	H334

BT_0474_Alticinae sp. 80	Alticinae sp. 80	Network146	3Cluster128	5Cluster126	75Cluster125	GMYC156	PTP171	H225
BT_0475_Eumolpinae sp. 39	Eumolpinae sp. 39	Network267	3Cluster100	5Cluster100	75Cluster099	GMYC025	PTP028	H398
BT_0489_Galerucinae sp. 67	Galerucinae sp. 67	Network228	3Cluster129	5Cluster127	75Cluster126	GMYC085	PTP216	H348
BT_0490_Hispinae sp. 5	Hispinae sp. 5	Network246	3Cluster130	5Cluster128	75Cluster127	GMYC287	PTP034	H371
BT_0491_Alticinae sp. 157	Alticinae sp. 157	Network135	3Cluster131	5Cluster129	75Cluster128	GMYC169	PTP190	H209
BT_0492_Galerucinae sp. 61	Galerucinae sp. 61	Network93	3Cluster041	5Cluster042	75Cluster042	GMYC131	PTP279	H149
BT_0494_Alticinae sp. 250	Alticinae sp. 250	Network74	3Cluster132	5Cluster130	75Cluster129	GMYC039	PTP146	H115
BT_0496_Alticinae sp. 112	Alticinae sp. 112	Network220	3Cluster133	5Cluster131	75Cluster130	GMYC071	PTP214	H336
BT_0499_Alticinae sp. 44	Alticinae sp. 44	Network42	3Cluster024	5Cluster025	75Cluster025	GMYC214	PTP102	H060
BT_0501_Galerucinae sp. 66	Galerucinae sp. 66	Network223	3Cluster059	5Cluster060	75Cluster060	GMYC079	PTP222	H341
BT_0502_Eumolpinae sp. 42	Eumolpinae sp. 42	Network257	3Cluster003	5Cluster003	75Cluster003	GMYC001	PTP003	H388
BT_0503_Alticinae sp. 111	Alticinae sp. 111	Network137	3Cluster134	5Cluster132	75Cluster131	GMYC192	PTP213	H212
BT_0505_Alticinae sp. 96	Alticinae sp. 96	Network85	3Cluster044	5Cluster045	75Cluster045	GMYC051	PTP169	H131
BT_0506_Alticinae sp. 92	Alticinae sp. 92	Network200	3Cluster135	5Cluster133	75Cluster132	GMYC191	PTP208	H309
BT_0508_Galerucinae sp. 53	Galerucinae sp. 53	Network205	3Cluster029	5Cluster030	75Cluster030	GMYC111	PTP239	H317
BT_0510_Cassidinae sp. 4	Cassidinae sp. 4	Network253	3Cluster038	5Cluster039	75Cluster039	GMYC284	PTP053	H379
BT_0511_Cassidinae sp. 8	Cassidinae sp. 8	Network241	3Cluster136	5Cluster134	75Cluster133	GMYC286	PTP032	H363
BT_0512_Hispinae sp. 6	Hispinae sp. 6	Network251	3Cluster137	5Cluster135	75Cluster134	GMYC269	PTP048	H376
BT_0514_Eumolpinae sp. 074	Eumolpinae sp. 074	Network244	3Cluster062	5Cluster063	75Cluster063	GMYC031	PTP002	H368
BT_0516_Alticinae sp. 126	Alticinae sp. 126	Network220	3Cluster133	5Cluster131	75Cluster130	GMYC071	PTP214	H336
BT_0517_Alticinae sp. 104	Alticinae sp. 104	Network17	3Cluster101	5Cluster101	75Cluster100	GMYC235	PTP080	H027
BT_0518_Alticinae sp. 85	Alticinae sp. 85	Network47	3Cluster138	5Cluster136	75Cluster135	GMYC217	PTP104	H071
BT_0519_Alticinae sp. 68	Alticinae sp. 68	Network84	3Cluster139	5Cluster137	75Cluster136	GMYC052	PTP162	H127
BT_0520_Alticinae sp. 159	Alticinae sp. 159	Network133	3Cluster140	5Cluster138	75Cluster137	GMYC164	PTP180	H207
BT_0524_Eumolpinae sp. 39	Eumolpinae sp. 39	Network268	3Cluster141	5Cluster139	75Cluster099	GMYC026	PTP029	H399
BT_0525_Hispinae sp. 023	Hispinae sp. 023	Network248	3Cluster142	5Cluster140	75Cluster067	GMYC271	PTP049	H373
BT_0526_Eumolpinae sp. 39	Eumolpinae sp. 39	Network267	3Cluster100	5Cluster100	75Cluster099	GMYC025	PTP028	H398
BT_0527_Galerucinae sp. 36	Galerucinae sp. 36	Network208	3Cluster143	5Cluster141	75Cluster138	GMYC146	PTP254	H320
BT_0528_Alticinae sp. 150	Alticinae sp. 150	Network191	3Cluster115	5Cluster011	75Cluster011	GMYC088	PTP203	H290
BT_0529_Alticinae sp. 113	Alticinae sp. 113	Network204	3Cluster144	5Cluster142	75Cluster139	GMYC064	PTP140	H316
BT_0530_Galerucinae sp. 31	Galerucinae sp. 31	Network203	3Cluster061	5Cluster062	75Cluster062	GMYC084	PTP217	H312
BT_0531_Galerucinae sp. 62	Galerucinae sp. 62	Network205	3Cluster029	5Cluster030	75Cluster030	GMYC111	PTP239	H317
BT_0532_Galerucinae sp. 75	Galerucinae sp. 75	Network227	3Cluster145	5Cluster143	75Cluster110	GMYC082	PTP219	H346
BT_0533_Alticinae sp. 97	Alticinae sp. 97	Network13	3Cluster035	5Cluster036	75Cluster036	GMYC258	PTP065	H017
BT_0535_Alticinae sp. 97	Alticinae sp. 97	Network13	3Cluster035	5Cluster036	75Cluster036	GMYC258	PTP065	H019
BT_0537_Alticinae sp. 142	Alticinae sp. 142	Network132	3Cluster081	5Cluster081	75Cluster080	GMYC167	PTP189	H205
BT_0538_Alticinae sp. 238	Alticinae sp. 238	Network176	3Cluster146	5Cluster144	75Cluster140	GMYC189	PTP210	H273
BT_0539_Alticinae sp. 13	Alticinae sp. 13	Network175	3Cluster147	5Cluster156	75Cluster141	GMYC066	PTP090	H271
BT_0540_Galerucinae sp. 64	Galerucinae sp. 64	Network222	3Cluster148	5Cluster145	75Cluster060	GMYC080	PTP221	H339
BT_0544_Cassidinae sp. 12	Cassidinae sp. 12	Network243	3Cluster149	5Cluster146	75Cluster142	GMYC278	PTP044	H366
BT_0546_Alticinae sp. 83	Alticinae sp. 83	Network22	3Cluster083	5Cluster083	75Cluster082	GMYC230	PTP072	H034
BT_0547_Alticinae sp. 96	Alticinae sp. 96	Network85	3Cluster044	5Cluster045	75Cluster045	GMYC051	PTP169	H131
BT_0549_Alticinae sp. 150	Alticinae sp. 150	Network191	3Cluster115	5Cluster011	75Cluster011	GMYC088	PTP203	H292
BT_0550_Alticinae sp. 265	Alticinae sp. 265	Network60	3Cluster150	5Cluster147	75Cluster143	GMYC068	PTP142	H096
BT_0551_Galerucinae sp. 64	Galerucinae sp. 64	Network222	3Cluster148	5Cluster145	75Cluster060	GMYC080	PTP221	H339
BT_0552_Alticinae sp. 117	Alticinae sp. 117	Network87	3Cluster032	5Cluster033	75Cluster033	GMYC048	PTP166	H133
BT_0553_Eumolpinae sp. 42	Eumolpinae sp. 42	Network257	3Cluster003	5Cluster003	75Cluster003	GMYC001	PTP003	H388
BT_0554_Galerucinae sp. 71	Galerucinae sp. 71	Network233	3Cluster151	5Cluster148	75Cluster053	GMYC075	PTP227	H354
BT_0555_Alticinae sp. 86	Alticinae sp. 86	Network48	3Cluster074	5Cluster075	75Cluster074	GMYC216	PTP105	H078
BT_0556_Alticinae sp. 96	Alticinae sp. 96	Network85	3Cluster044	5Cluster045	75Cluster045	GMYC051	PTP169	H131
BT_0557_Alticinae sp. 181	Alticinae sp. 181	Network220	3Cluster133	5Cluster131	75Cluster130	GMYC071	PTP214	H336
BT_0558_Alticinae sp. 149	Alticinae sp. 149	Network191	3Cluster115	5Cluster011	75Cluster011	GMYC088	PTP203	H293
BT_0559_Eumolpinae sp. 42	Eumolpinae sp. 42	Network257	3Cluster003	5Cluster003	75Cluster003	GMYC001	PTP003	H388
BT_0560_Galerucinae sp. 64	Galerucinae sp. 64	Network222	3Cluster148	5Cluster145	75Cluster060	GMYC080	PTP221	H339
BT_0561_Galerucinae sp. 66	Galerucinae sp. 66	Network224	3Cluster152	5Cluster149	75Cluster060	GMYC077	PTP224	H343
BT_0565_Alticinae sp. 150	Alticinae sp. 150	Network191	3Cluster115	5Cluster011	75Cluster011	GMYC088	PTP203	H289
BT_0566_Hispinae sp. 6	Hispinae sp. 6	Network251	3Cluster137	5Cluster135	75Cluster134	GMYC269	PTP048	H377
BT_0567_Alticinae sp. 104	Alticinae sp. 104	Network17	3Cluster101	5Cluster101	75Cluster100	GMYC235	PTP080	H027
BT_0574_Eumolpinae sp. 39	Eumolpinae sp. 39	Network267	3Cluster100	5Cluster100	75Cluster099	GMYC025	PTP028	H398
BT_0575_Alticinae sp. 140	Alticinae sp. 140	Network122	3Cluster153	5Cluster150	75Cluster144	GMYC157	PTP187	H191
BT_0577_Eumolpinae sp. 39	Eumolpinae sp. 39	Network267	3Cluster100	5Cluster100	75Cluster099	GMYC025	PTP028	H398
BT_0579_Alticinae sp. 104	Alticinae sp. 104	Network17	3Cluster101	5Cluster101	75Cluster100	GMYC235	PTP080	H027
BT_0587_Alticinae sp. 6	Alticinae sp. 6	Network128	3Cluster154	5Cluster151	75Cluster145	GMYC153	PTP099	H201
BT_0588_Galerucinae sp. 69	Galerucinae sp. 69	Network219	3Cluster057	5Cluster058	75Cluster058	GMYC086	PTP215	H335
BT_0589_Eumolpinae sp. 17	Eumolpinae sp. 17	Network277	3Cluster079	5Cluster079	75Cluster078	GMYC018	PTP027	H410
BT_0590_Alticinae sp. 140	Alticinae sp. 140	Network122	3Cluster153	5Cluster150	75Cluster144	GMYC157	PTP187	H191
BT_0592_Eumolpinae sp. 39	Eumolpinae sp. 39	Network267	3Cluster100	5Cluster100	75Cluster099	GMYC025	PTP028	H398
BT_0594_Alticinae sp. 118	Alticinae sp. 118	Network169	3Cluster155	5Cluster152	75Cluster054	GMYC188	PTP109	H260
BT_0596_Eumolpinae sp. 39	Eumolpinae sp. 39	Network267	3Cluster100	5Cluster100	75Cluster099	GMYC025	PTP028	H398

BT_0597_Alticinae sp. 104	Alticinae sp. 104	Network17	3Cluster101	5Cluster101	75Cluster100	GMYC235	PTP080	H027
BT_0604_Hispinae sp. 5	Hispinae sp. 5	Network246	3Cluster130	5Cluster128	75Cluster127	GMYC287	PTP034	H371
BT_0605_Alticinae sp. 87	Alticinae sp. 87	Network88	3Cluster028	5Cluster029	75Cluster029	GMYC061	PTP164	H136
BT_0606_Eumolpinae sp. 39	Eumolpinae sp. 39	Network267	3Cluster100	5Cluster100	75Cluster099	GMYC025	PTP028	H398
BT_0628_Cassidinae sp. 12	Cassidinae sp. 12	Network243	3Cluster149	5Cluster146	75Cluster142	GMYC278	PTP044	H366
BT_0630_Alticinae sp. 36	Alticinae sp. 36	Network142	3Cluster156	5Cluster153	75Cluster146	GMYC035	PTP088	H220
BT_0631_Alticinae sp. 64	Alticinae sp. 64	Network75	3Cluster157	5Cluster154	75Cluster101	GMYC042	PTP148	H116
BT_0632_Alticinae sp. 86	Alticinae sp. 86	Network48	3Cluster074	5Cluster075	75Cluster074	GMYC216	PTP105	H075
BT_0633_Alticinae sp. 85	Alticinae sp. 85	Network48	3Cluster074	5Cluster075	75Cluster074	GMYC216	PTP105	H078
BT_0634_Alticinae sp. 66	Alticinae sp. 66	Network85	3Cluster044	5Cluster045	75Cluster045	GMYC051	PTP169	H131
BT_0635_Alticinae sp. 96	Alticinae sp. 96	Network85	3Cluster044	5Cluster045	75Cluster045	GMYC051	PTP169	H131
BT_0640_Alticinae sp. 18	Alticinae sp. 18	Network59	3Cluster158	5Cluster155	75Cluster147	GMYC251	PTP067	H095
BT_0641_Alticinae sp. 13	Alticinae sp. 13	Network174	3Cluster159	5Cluster156	75Cluster141	GMYC067	PTP089	H269
BT_0642_Eumolpinae sp. 42	Eumolpinae sp. 42	Network257	3Cluster003	5Cluster003	75Cluster003	GMYC001	PTP003	H385
BT_0644_Galerucinae sp. 66	Galerucinae sp. 66	Network223	3Cluster059	5Cluster060	75Cluster060	GMYC079	PTP222	H342
BT_0645_Galerucinae sp. 11	Galerucinae sp. 11	Network113	3Cluster160	5Cluster157	75Cluster148	GMYC140	PTP260	H178
BT_0646_Alticinae sp. 71	Alticinae sp. 71	Network44	3Cluster161	5Cluster158	75Cluster149	GMYC215	PTP123	H064
BT_0647_Galerucinae sp. 35	Galerucinae sp. 35	Network166	3Cluster162	5Cluster159	75Cluster150	GMYC109	PTP241	H257
BT_0648_Alticinae sp. 140	Alticinae sp. 140	Network122	3Cluster153	5Cluster150	75Cluster144	GMYC157	PTP187	H190
BT_0650_Eumolpinae sp. 39	Eumolpinae sp. 39	Network267	3Cluster100	5Cluster100	75Cluster099	GMYC025	PTP028	H398
BT_0652_Alticinae sp. 104	Alticinae sp. 104	Network17	3Cluster101	5Cluster101	75Cluster100	GMYC235	PTP080	H026
BT_0655_Alticinae sp. 257	Alticinae sp. 257	Network73	3Cluster163	5Cluster160	75Cluster151	GMYC038	PTP151	H114
BT_0656_Alticinae sp. 51	Alticinae sp. 51	Network31	3Cluster164	5Cluster161	75Cluster152	GMYC207	PTP135	H045
BT_0657_Eumolpinae sp. 39	Eumolpinae sp. 39	Network267	3Cluster100	5Cluster100	75Cluster099	GMYC025	PTP028	H398
BT_0660_Alticinae sp. 104	Alticinae sp. 104	Network17	3Cluster101	5Cluster101	75Cluster100	GMYC235	PTP080	H027
BT_0662_Alticinae sp. 126	Alticinae sp. 126	Network14	3Cluster165	5Cluster162	75Cluster153	GMYC246	PTP093	H022
BT_0663_Alticinae sp. 150	Alticinae sp. 150	Network191	3Cluster115	5Cluster011	75Cluster011	GMYC088	PTP203	H289
BT_0664_Alticinae sp. 150	Alticinae sp. 150	Network191	3Cluster115	5Cluster011	75Cluster011	GMYC088	PTP203	H295
BT_0665_Alticinae sp. 150	Alticinae sp. 150	Network191	3Cluster115	5Cluster011	75Cluster011	GMYC088	PTP203	H292
BT_0675_Alticinae sp. 8	Alticinae sp. 8	Network189	3Cluster166	5Cluster163	75Cluster011	GMYC090	PTP201	H287
BT_0676_Alticinae sp. 64	Alticinae sp. 64	Network79	3Cluster045	5Cluster046	75Cluster046	GMYC037	PTP147	H122
BT_0677_Eumolpinae sp. 39	Eumolpinae sp. 39	Network267	3Cluster100	5Cluster100	75Cluster099	GMYC025	PTP028	H398
BT_0680_Galerucinae sp. 76	Galerucinae sp. 76	Network118	3Cluster008	5Cluster008	75Cluster008	GMYC113	PTP288	H185
BT_0683_Alticinae sp. 115	Alticinae sp. 115	Network10	3Cluster030	5Cluster031	75Cluster031	GMYC253	PTP060	H013
BT_0686_Eumolpinae sp. 23	Eumolpinae sp. 23	Network270	3Cluster007	5Cluster007	75Cluster007	GMYC022	PTP007	H402
BT_0687_Alticinae sp. 87	Alticinae sp. 87	Network88	3Cluster028	5Cluster029	75Cluster029	GMYC061	PTP164	H136
BT_0688_Criocerinae sp. 1	Criocerinae sp. 1	Network237	3Cluster071	5Cluster072	75Cluster071	GMYC264	PTP057	H359
BT_0690_Alticinae sp. 124	Alticinae sp. 124	Network87	3Cluster032	5Cluster033	75Cluster033	GMYC048	PTP166	H134
BT_0691_Galerucinae sp. 31	Galerucinae sp. 31	Network203	3Cluster061	5Cluster062	75Cluster062	GMYC084	PTP217	H314
BT_0692_Alticinae sp. 14	Alticinae sp. 14	Network131	3Cluster167	5Cluster164	75Cluster154	GMYC162	PTP188	H204
BT_0698_Alticinae sp. 13	Alticinae sp. 13	Network174	3Cluster159	5Cluster156	75Cluster141	GMYC067	PTP089	H270
BT_0699_Alticinae sp. 83	Alticinae sp. 83	Network22	3Cluster083	5Cluster083	75Cluster082	GMYC230	PTP072	H034
BT_0705_Galerucinae sp. 8	Galerucinae sp. 8	Network107	3Cluster168	5Cluster165	75Cluster155	GMYC136	PTP266	H166
BT_0709_Galerucinae sp. 64	Galerucinae sp. 64	Network222	3Cluster148	5Cluster145	75Cluster060	GMYC080	PTP221	H339
BT_0710_Alticinae sp. 13	Alticinae sp. 13	Network175	3Cluster147	5Cluster156	75Cluster141	GMYC066	PTP090	H272
BT_0711_Galerucinae sp. 73	Galerucinae sp. 73	Network221	3Cluster169	5Cluster166	75Cluster156	GMYC072	PTP220	H337
BT_0713_Galerucinae sp. 73	Galerucinae sp. 73	Network221	3Cluster169	5Cluster166	75Cluster156	GMYC072	PTP220	H337
BT_0715_Galerucinae sp. 76	Galerucinae sp. 76	Network118	3Cluster008	5Cluster008	75Cluster008	GMYC113	PTP288	H185
BT_0716_Galerucinae sp. 11	Galerucinae sp. 11	Network103	3Cluster015	5Cluster016	75Cluster016	GMYC143	PTP258	H161
BT_0717_Galerucinae sp. 002	Galerucinae sp. 002	Network114	3Cluster170	5Cluster167	75Cluster157	GMYC141	PTP262	H179
BT_0719_Eumolpinae sp. 43	Eumolpinae sp. 43	Network234	3Cluster086	5Cluster086	75Cluster085	GMYC020	PTP009	H355
BT_0721_Eumolpinae sp. 20	Eumolpinae sp. 20	Network289	3Cluster077	5Cluster078	75Cluster077	GMYC003	PTP010	H426
BT_0722_Cassidinae sp. 13	Cassidinae sp. 13	Network235	3Cluster171	5Cluster168	75Cluster158	GMYC275	PTP041	H356
BT_0725_Alticinae sp. 243	Alticinae sp. 243	Network53	3Cluster006	5Cluster006	75Cluster006	GMYC221	PTP116	H087
BT_0727_Galerucinae sp. 73	Galerucinae sp. 73	Network221	3Cluster169	5Cluster166	75Cluster156	GMYC072	PTP220	H338
BT_0728_Galerucinae sp. 64	Galerucinae sp. 64	Network222	3Cluster148	5Cluster145	75Cluster060	GMYC080	PTP221	H339
BT_0729_Galerucinae sp. 15	Galerucinae sp. 15	Network202	3Cluster120	5Cluster118	75Cluster117	GMYC105	PTP234	H311
BT_0730_Alticinae sp. 136	Alticinae sp. 136	Network165	3Cluster172	5Cluster169	75Cluster159	GMYC159	PTP185	H255
BT_0732_Galerucinae sp. 4	Galerucinae sp. 4	Network106	3Cluster173	5Cluster170	75Cluster160	GMYC145	PTP257	H165
BT_0733_Galerucinae sp. 097	Galerucinae sp. 097	Network108	3Cluster174	5Cluster171	75Cluster020	GMYC135	PTP268	H167
BT_0734_Galerucinae sp. 39	Galerucinae sp. 39	Network97	3Cluster017	5Cluster018	75Cluster018	GMYC118	PTP271	H153
BT_0735_Galerucinae sp. 38	Galerucinae sp. 38	Network99	3Cluster175	5Cluster005	75Cluster005	GMYC116	PTP273	H155
BT_0736_Galerucinae sp. 26	Galerucinae sp. 26	Network211	3Cluster176	5Cluster172	75Cluster161	GMYC104	PTP233	H324
BT_0738_Eumolpinae sp. 4	Eumolpinae sp. 4	Network273	3Cluster177	5Cluster173	75Cluster162	GMYC024	PTP005	H405
BT_0739_Galerucinae sp. 18	Galerucinae sp. 18	Network96	3Cluster178	5Cluster174	75Cluster163	GMYC119	PTP270	H152
BT_0740_Galerucinae sp. 76	Galerucinae sp. 76	Network118	3Cluster008	5Cluster008	75Cluster008	GMYC113	PTP288	H185
BT_0741_Galerucinae sp. 002	Galerucinae sp. 002	Network112	3Cluster011	5Cluster012	75Cluster012	GMYC138	PTP264	H175
BT_0742_Galerucinae sp. 19	Galerucinae sp. 19	Network120	3Cluster179	5Cluster175	75Cluster164	GMYC112	PTP285	H187
BT_0743_Galerucinae sp. 14	Galerucinae sp. 14	Network102	3Cluster180	5Cluster176	75Cluster165	GMYC132	PTP255	H159

BT_0744_Galerucinae sp. 7	Galerucinae sp. 7	Network109	3Cluster019	5Cluster020	75Cluster020	GMYC134	PTP267	H171
BT_0747_Galerucinae sp. 38	Galerucinae sp. 38	Network99	3Cluster175	5Cluster005	75Cluster005	GMYC116	PTP273	H156
BT_0749_Galerucinae sp. 39	Galerucinae sp. 39	Network97	3Cluster017	5Cluster018	75Cluster018	GMYC118	PTP271	H153
BT_0750_Cassidinae sp. 5	Cassidinae sp. 5	Network242	3Cluster058	5Cluster059	75Cluster059	GMYC277	PTP043	H365
BT_0752_Alticinae sp. 39	Alticinae sp. 39	Network163	3Cluster181	5Cluster177	75Cluster166	GMYC094	PTP198	H253
BT_0753_Alticinae sp. 10	Alticinae sp. 10	Network195	3Cluster021	5Cluster022	75Cluster022	GMYC097	PTP195	H304
BT_0755_Galerucinae sp. 36	Galerucinae sp. 36	Network207	3Cluster182	5Cluster178	75Cluster138	GMYC147	PTP253	H319
BT_0756_Eumolpinae sp. 22	Eumolpinae sp. 22	Network269	3Cluster183	5Cluster179	75Cluster167	GMYC023	PTP006	H400
BT_0766_Alticinae sp. 96	Alticinae sp. 96	Network85	3Cluster044	5Cluster045	75Cluster045	GMYC051	PTP169	H131
BT_0770_Eumolpinae sp. 16	Eumolpinae sp. 16	Network286	3Cluster184	5Cluster180	75Cluster168	GMYC002	PTP012	H422
BT_0779_Alticinae sp. 115	Alticinae sp. 115	Network10	3Cluster030	5Cluster031	75Cluster031	GMYC253	PTP060	H010
BT_0781_Eumolpinae sp. 20	Eumolpinae sp. 20	Network289	3Cluster077	5Cluster078	75Cluster077	GMYC003	PTP010	H425
BT_0782_Galerucinae sp. 34	Galerucinae sp. 34	Network183	3Cluster037	5Cluster038	75Cluster038	GMYC103	PTP232	H280
BT_0788_Alticinae sp. 6	Alticinae sp. 6	Network125	3Cluster185	5Cluster181	75Cluster169	GMYC154	PTP100	H198
BT_0789_Alticinae sp.14	Alticinae sp.14	Network131	3Cluster167	5Cluster164	75Cluster154	GMYC162	PTP188	H204
BT_0791_Eumolpinae sp. 16	Eumolpinae sp. 16	Network286	3Cluster184	5Cluster180	75Cluster168	GMYC002	PTP012	H421
BT_0792_Alticinae sp. 115	Alticinae sp. 115	Network10	3Cluster030	5Cluster031	75Cluster031	GMYC253	PTP060	H010
BT_0794_Alticinae sp. 129	Alticinae sp. 129	Network212	3Cluster034	5Cluster035	75Cluster035	GMYC065	PTP098	H325
BT_0795_Alticinae sp. 96	Alticinae sp. 96	Network85	3Cluster044	5Cluster045	75Cluster045	GMYC051	PTP169	H128
BT_0796_Alticinae sp. 97	Alticinae sp. 97	Network13	3Cluster035	5Cluster036	75Cluster036	GMYC258	PTP065	H018
BT_0799_Cassidinae sp. 14	Cassidinae sp. 14	Network263	3Cluster186	5Cluster065	75Cluster065	GMYC282	PTP040	H394
BT_0800_Galerucinae sp. 30	Galerucinae sp. 30	Network184	3Cluster070	5Cluster071	75Cluster070	GMYC110	PTP240	H282
BT_0803_Galerucinae sp. 76	Galerucinae sp. 76	Network118	3Cluster008	5Cluster008	75Cluster008	GMYC113	PTP288	H185
BT_0804_Alticinae sp. 28	Alticinae sp. 28	Network29	3Cluster039	5Cluster040	75Cluster040	GMYC209	PTP129	H042
BT_0805_Alticinae sp. 14	Alticinae sp. 14	Network131	3Cluster167	5Cluster164	75Cluster154	GMYC162	PTP188	H204
BT_0806_Alticinae sp. 13	Alticinae sp. 13	Network175	3Cluster147	5Cluster156	75Cluster141	GMYC066	PTP090	H271
BT_0807_Alticinae sp. 54	Alticinae sp. 54	Network44	3Cluster161	5Cluster158	75Cluster149	GMYC215	PTP123	H063
BT_0809_Alticinae sp. 12	Alticinae sp. 12	Network150	3Cluster187	5Cluster182	75Cluster170	GMYC239	PTP084	H231
BT_0810_Galerucinae sp. 46	Galerucinae sp. 46	Network156	3Cluster050	5Cluster051	75Cluster051	GMYC179	PTP250	H242
BT_0811_Criocerinae sp. 6	Criocerinae sp. 6	Network238	3Cluster188	5Cluster183	75Cluster171	GMYC266	PTP058	H360
BT_0813_Criocerinae sp. 4	Criocerinae sp. 4	Network239	3Cluster189	5Cluster184	75Cluster172	GMYC262	PTP055	H361
BT_0814_Alticinae sp. 86	Alticinae sp. 86	Network48	3Cluster074	5Cluster075	75Cluster074	GMYC216	PTP105	H078
BT_0815_Alticinae sp. 32	Alticinae sp. 32	Network150	3Cluster187	5Cluster182	75Cluster170	GMYC239	PTP084	H232
BT_0816_Galerucinae sp. 19	Galerucinae sp. 19	Network120	3Cluster179	5Cluster175	75Cluster164	GMYC112	PTP285	H188
BT_0817_Alticinae sp. 87	Alticinae sp. 87	Network88	3Cluster028	5Cluster029	75Cluster029	GMYC061	PTP164	H139
BT_0818_Galerucinae sp. 61	Galerucinae sp. 61	Network93	3Cluster041	5Cluster042	75Cluster042	GMYC131	PTP279	H149
BT_0819_Eumolpinae sp. 074	Eumolpinae sp. 074	Network244	3Cluster062	5Cluster063	75Cluster063	GMYC031	PTP002	H369
BT_0820_Eumolpinae sp. 38	Eumolpinae sp. 38	Network284	3Cluster025	5Cluster026	75Cluster026	GMYC010	PTP017	H418
BT_0823_Galerucinae sp. 34	Galerucinae sp. 34	Network183	3Cluster037	5Cluster038	75Cluster038	GMYC103	PTP232	H281
BT_0827_Eumolpinae sp. 20	Eumolpinae sp. 20	Network289	3Cluster077	5Cluster078	75Cluster077	GMYC003	PTP010	H425
BT_0828_Alticinae sp. 118	Alticinae sp. 118	Network169	3Cluster155	5Cluster152	75Cluster054	GMYC188	PTP109	H260
BT_0829_Alticinae sp. 115	Alticinae sp. 115	Network10	3Cluster030	5Cluster031	75Cluster031	GMYC253	PTP060	H010
BT_0831_Alticinae sp. 147	Alticinae sp. 147	Network65	3Cluster190	5Cluster185	75Cluster173	GMYC194	PTP211	H104
BT_0835_Galerucinae sp. 29	Galerucinae sp. 29	Network94	3Cluster191	5Cluster186	75Cluster174	GMYC125	PTP275	H150
BT_0836_Alticinae sp. 3	Alticinae sp. 3	Network185	3Cluster192	5Cluster187	75Cluster175	GMYC095	PTP197	H283
BT_0837_Alticinae sp. 9	Alticinae sp. 9	Network192	3Cluster096	5Cluster096	75Cluster095	GMYC099	PTP196	H298
BT_0839_Alticinae sp. 40	Alticinae sp. 40	Network56	3Cluster193	5Cluster188	75Cluster176	GMYC223	PTP118	H091
BT_0840_Alticinae sp. 140	Alticinae sp. 140	Network123	3Cluster122	5Cluster120	75Cluster119	GMYC158	PTP186	H194
BT_0841_Alticinae sp. 127	Alticinae sp. 127	Network4	3Cluster194	5Cluster189	75Cluster177	GMYC247	PTP092	H004
BT_0843_Alticinae sp. 136	Alticinae sp. 136	Network165	3Cluster172	5Cluster169	75Cluster159	GMYC159	PTP185	H256
BT_0847_Eumolpinae sp. 5	Eumolpinae sp. 5	Network285	3Cluster013	5Cluster014	75Cluster014	GMYC009	PTP016	H419
BT_0848_Alticinae sp. 45	Alticinae sp. 45	Network55	3Cluster195	5Cluster190	75Cluster178	GMYC225	PTP119	H090
BT_0850_Alticinae sp. 49	Alticinae sp. 49	Network141	3Cluster196	5Cluster191	75Cluster179	GMYC032	PTP085	H218
BT_0851_Alticinae sp. 65	Alticinae sp. 65	Network85	3Cluster044	5Cluster045	75Cluster045	GMYC051	PTP169	H131
BT_0852_Alticinae sp. 133	Alticinae sp. 133	Network130	3Cluster076	5Cluster077	75Cluster076	GMYC170	PTP192	H203
BT_0855_Alticinae sp. 018	Alticinae sp. 018	Network59	3Cluster158	5Cluster155	75Cluster147	GMYC251	PTP067	H095
BT_0861_Eumolpinae sp. 20	Eumolpinae sp. 20	Network289	3Cluster077	5Cluster078	75Cluster077	GMYC003	PTP010	H425
BT_0862_Alticinae sp. 115	Alticinae sp. 115	Network10	3Cluster030	5Cluster031	75Cluster031	GMYC253	PTP060	H010
BT_0864_Alticinae sp. 253	Alticinae sp. 253	Network13	3Cluster035	5Cluster036	75Cluster036	GMYC258	PTP065	H018
BT_0865_Alticinae sp. 96	Alticinae sp. 96	Network85	3Cluster044	5Cluster045	75Cluster045	GMYC051	PTP169	H131
BT_0871_Alticinae sp. 86	Alticinae sp. 86	Network48	3Cluster074	5Cluster075	75Cluster074	GMYC216	PTP105	H074
BT_0873_Alticinae sp. 85	Alticinae sp. 85	Network48	3Cluster074	5Cluster075	75Cluster074	GMYC216	PTP105	H077
BT_0949_Cassidinae sp. 2	Cassidinae sp. 2	Network259	3Cluster197	5Cluster192	75Cluster180	GMYC283	PTP037	H390
BT_0950_Cassidinae sp. 10	Cassidinae sp. 10	Network252	3Cluster198	5Cluster193	75Cluster181	GMYC274	PTP036	H378
BT_0951_Cassidinae sp. 11	Cassidinae sp. 11	Network260	3Cluster199	5Cluster194	75Cluster182	GMYC279	PTP045	H391
BT_0952_Alticinae sp. 269	Alticinae sp. 269	Network19	3Cluster200	5Cluster195	75Cluster183	GMYC228	PTP121	H030
BT_0953_Galerucinae sp. 28	Galerucinae sp. 28	Network63	3Cluster201	5Cluster196	75Cluster103	GMYC129	PTP282	H099
BT_0954_Galerucinae sp. 29	Galerucinae sp. 29	Network95	3Cluster202	5Cluster197	75Cluster184	GMYC124	PTP276	H151
BT_0955_Galerucinae sp. 20	Galerucinae sp. 20	Network119	3Cluster203	5Cluster198	75Cluster185	GMYC122	PTP277	H186

BT_0957_Galerucinae sp. 36	Galerucinae sp. 36	Network208	3Cluster143	5Cluster141	75Cluster138	GMYC146	PTP254	H321
BT_0958_Alticinae sp. 154	Alticinae sp. 154	Network43	3Cluster204	5Cluster199	75Cluster186	GMYC226	PTP120	H061
BT_0959_Galerucinae sp. 52	Galerucinae sp. 52	Network201	3Cluster087	5Cluster087	75Cluster086	GMYC107	PTP231	H310
BT_0960_Galerucinae sp. 47	Galerucinae sp. 47	Network157	3Cluster205	5Cluster200	75Cluster187	GMYC180	PTP242	H243
BT_0961_Alticinae sp. 77	Alticinae sp. 77	Network86	3Cluster206	5Cluster201	75Cluster188	GMYC047	PTP165	H132
BT_0962_Alticinae sp. 76	Alticinae sp. 76	Network27	3Cluster207	5Cluster202	75Cluster189	GMYC210	PTP127	H039
BT_0964_Alticinae sp. 30	Alticinae sp. 30	Network23	3Cluster208	5Cluster203	75Cluster190	GMYC232	PTP074	H035
BT_0965_Criocerinae sp. 2	Criocerinae sp. 2	Network236	3Cluster209	5Cluster204	75Cluster191	GMYC265	PTP054	H357
BT_0966_Criocerinae sp. 3	Criocerinae sp. 3	Network3	3Cluster210	5Cluster205	75Cluster192	GMYC030	PTP031	H003
BT_0967_Eumolpinae sp. 46	Eumolpinae sp. 46	Network281	3Cluster211	5Cluster206	75Cluster193	GMYC006	PTP015	H415
BT_0968_Alticinae sp. 99	Alticinae sp. 99	Network66	3Cluster212	5Cluster207	75Cluster194	GMYC046	PTP170	H105
BT_0971_Alticinae sp. 194	Alticinae sp. 194	Network25	3Cluster213	5Cluster208	75Cluster195	GMYC202	PTP130	H037
BT_0972_Alticinae sp. 127	Alticinae sp. 127	Network4	3Cluster194	5Cluster189	75Cluster177	GMYC247	PTP092	H004
BT_0973_Alticinae sp. 97	Alticinae sp. 97	Network9	3Cluster214	5Cluster209	75Cluster196	GMYC252	PTP059	H009
BT_1030_Alticinae sp. 240	Alticinae sp. 240	Network20	3Cluster215	5Cluster210	75Cluster197	GMYC229	PTP073	H031
BT_1031_Alticinae sp. 2	Alticinae sp. 2	Network187	3Cluster010	5Cluster010	75Cluster010	GMYC087	PTP204	H285
BT_1033_Alticinae sp. 149	Alticinae sp. 149	Network191	3Cluster115	5Cluster011	75Cluster011	GMYC088	PTP203	H294
BT_1034_Alticinae sp. 48	Alticinae sp. 48	Network44	3Cluster161	5Cluster158	75Cluster149	GMYC215	PTP123	H062
BT_1035_Alticinae sp. 150	Alticinae sp. 150	Network191	3Cluster115	5Cluster011	75Cluster011	GMYC088	PTP203	H295
BT_1036_Alticinae sp. 62	Alticinae sp. 62	Network57	3Cluster043	5Cluster044	75Cluster044	GMYC224	PTP117	H093
BT_1038_Alticinae sp. 65	Alticinae sp. 65	Network85	3Cluster044	5Cluster045	75Cluster045	GMYC051	PTP169	H131
BT_1043_Alticinae sp. 66	Alticinae sp. 66	Network85	3Cluster044	5Cluster045	75Cluster045	GMYC051	PTP169	H131
BT_1055_Alticinae sp. 85	Alticinae sp. 85	Network48	3Cluster074	5Cluster075	75Cluster074	GMYC216	PTP105	H075
BT_1060_Alticinae sp. 85	Alticinae sp. 85	Network48	3Cluster074	5Cluster075	75Cluster074	GMYC216	PTP105	H079
BT_1061_Galerucinae sp. 096	Galerucinae sp. 096	Network180	3Cluster049	5Cluster050	75Cluster050	GMYC174	PTP252	H277
BT_1064_Alticinae sp. 142	Alticinae sp. 142	Network132	3Cluster081	5Cluster081	75Cluster080	GMYC167	PTP189	H206
BT_1065_Alticinae sp. 20	Alticinae sp. 20	Network178	3Cluster216	5Cluster211	75Cluster198	GMYC152	PTP070	H275
BT_1070_Alticinae sp. 97	Alticinae sp. 97	Network13	3Cluster035	5Cluster036	75Cluster036	GMYC258	PTP065	H018
BT_1071_Alticinae sp. 96	Alticinae sp. 96	Network85	3Cluster044	5Cluster045	75Cluster045	GMYC051	PTP169	H131
BT_1079_Alticinae sp. 104	Alticinae sp. 104	Network17	3Cluster101	5Cluster101	75Cluster100	GMYC235	PTP080	H027
BT_1080_Eumolpinae sp. 074	Eumolpinae sp. 074	Network244	3Cluster062	5Cluster063	75Cluster063	GMYC031	PTP002	H368
BT_1081_Alticinae sp. 27	Alticinae sp. 27	Network51	3Cluster217	5Cluster212	75Cluster199	GMYC222	PTP113	H083
BT_1082_Alticinae sp. 104	Alticinae sp. 104	Network17	3Cluster101	5Cluster101	75Cluster100	GMYC235	PTP080	H026
BT_1083_Alticinae sp. 96	Alticinae sp. 96	Network85	3Cluster044	5Cluster045	75Cluster045	GMYC051	PTP169	H131
BT_1085_Alticinae sp. 61	Alticinae sp. 61	Network8	3Cluster068	5Cluster069	75Cluster069	GMYC259	PTP066	H008
BT_1086_Alticinae sp. 83	Alticinae sp. 83	Network22	3Cluster083	5Cluster083	75Cluster082	GMYC230	PTP072	H034
BT_1087_Alticinae sp. 109	Alticinae sp. 109	Network171	3Cluster046	5Cluster047	75Cluster047	GMYC254	PTP062	H265
BT_1088_Galerucinae sp. 31	Galerucinae sp. 31	Network203	3Cluster061	5Cluster062	75Cluster062	GMYC084	PTP217	H315
BT_1090_Alticinae sp. 19	Alticinae sp. 19	Network58	3Cluster218	5Cluster213	75Cluster200	GMYC245	PTP097	H094
BT_1091_Cassidinae sp. 12	Cassidinae sp. 12	Network243	3Cluster149	5Cluster146	75Cluster142	GMYC278	PTP044	H366
BT_1092_Cassidinae sp. 3	Cassidinae sp. 3	Network254	3Cluster219	5Cluster214	75Cluster201	GMYC285	PTP052	H381
BT_1093_Hispinae sp. 7	Hispinae sp. 7	Network258	3Cluster220	5Cluster215	75Cluster202	GMYC267	PTP035	H389
BT_1094_Eumolpinae sp. 38	Eumolpinae sp. 38	Network284	3Cluster025	5Cluster026	75Cluster026	GMYC010	PTP017	H418
BT_1095_Alticinae sp. 96	Alticinae sp. 96	Network85	3Cluster044	5Cluster045	75Cluster045	GMYC051	PTP169	H128
BT_1096_Galerucinae sp. 36	Galerucinae sp. 36	Network208	3Cluster143	5Cluster141	75Cluster138	GMYC146	PTP254	H320
BT_1098_Alticinae sp. 97	Alticinae sp. 97	Network13	3Cluster035	5Cluster036	75Cluster036	GMYC258	PTP065	H017
BT_1104_Alticinae sp. 140	Alticinae sp. 140	Network122	3Cluster153	5Cluster150	75Cluster144	GMYC157	PTP187	H190
BT_1105_Alticinae sp. 74	Alticinae sp. 74	Network177	3Cluster221	5Cluster216	75Cluster203	GMYC244	PTP096	H274
BT_1106_Galerucinae sp. 64	Galerucinae sp. 64	Network222	3Cluster148	5Cluster145	75Cluster060	GMYC080	PTP221	H340
BT_1107_Alticinae sp. 099	Alticinae sp. 099	Network173	3Cluster222	5Cluster217	75Cluster204	GMYC242	PTP207	H267
BT_1108_Alticinae sp. 92	Alticinae sp. 92	Network200	3Cluster135	5Cluster133	75Cluster132	GMYC191	PTP208	H309
BT_1109_Alticinae sp. 41	Alticinae sp. 41	Network136	3Cluster223	5Cluster218	75Cluster205	GMYC161	PTP177	H210
BT_1110_Galerucinae sp. 46	Galerucinae sp. 46	Network156	3Cluster050	5Cluster051	75Cluster051	GMYC179	PTP250	H242
BT_1112_Alticinae sp. 112	Alticinae sp. 112	Network145	3Cluster224	5Cluster219	75Cluster206	GMYC190	PTP209	H223
BT_1114_Alticinae sp. 41	Alticinae sp. 41	Network136	3Cluster223	5Cluster218	75Cluster205	GMYC161	PTP177	H210
BT_1117_Alticinae sp. 49	Alticinae sp. 49	Network141	3Cluster196	5Cluster191	75Cluster179	GMYC032	PTP085	H219
BT_1118_Alticinae sp. 123	Alticinae sp. 123	Network148	3Cluster036	5Cluster037	75Cluster037	GMYC070	PTP144	H229
BT_1119_Alticinae sp. 96	Alticinae sp. 96	Network85	3Cluster044	5Cluster045	75Cluster045	GMYC051	PTP169	H131
BT_1121_Alticinae sp. 124	Alticinae sp. 124	Network87	3Cluster032	5Cluster033	75Cluster033	GMYC048	PTP166	H133
BT_1122_Alticinae sp. 265	Alticinae sp. 265	Network60	3Cluster150	5Cluster147	75Cluster143	GMYC068	PTP142	H096
BT_1124_Cassidinae sp. 7	Cassidinae sp. 7	Network245	3Cluster065	5Cluster066	75Cluster066	GMYC276	PTP042	H370
BT_1125_Galerucinae sp. 59	Galerucinae sp. 59	Network197	3Cluster060	5Cluster061	75Cluster061	GMYC108	PTP230	H306
BT_1126_Hispinae sp. 3	Hispinae sp. 3	Network247	3Cluster066	5Cluster067	75Cluster067	GMYC272	PTP050	H372
BT_1127_Alticinae sp. 66	Alticinae sp. 66	Network85	3Cluster044	5Cluster045	75Cluster045	GMYC051	PTP169	H131
BT_1128_Alticinae sp. 18	Alticinae sp. 18	Network6	3Cluster225	5Cluster220	75Cluster207	GMYC260	PTP069	H006
BT_1129_Alticinae sp. 123	Alticinae sp. 123	Network148	3Cluster036	5Cluster037	75Cluster037	GMYC070	PTP144	H227
BT_1131_Alticinae sp. 081	Alticinae sp. 081	Network210	3Cluster084	5Cluster084	75Cluster083	GMYC069	PTP143	H323
BT_1132_Alticinae sp. 86	Alticinae sp. 86	Network48	3Cluster074	5Cluster075	75Cluster074	GMYC216	PTP105	H079
BT_1138_Alticinae sp. 86	Alticinae sp. 86	Network48	3Cluster074	5Cluster075	75Cluster074	GMYC216	PTP105	H075

BT_1142_Alticinae sp. 96	Alticinae sp. 96	Network85	3Cluster044	5Cluster045	75Cluster045	GMYC051	PTP169	H131
BT_1143_Hispinae sp. 7	Hispinae sp. 7	Network258	3Cluster220	5Cluster215	75Cluster202	GMYC267	PTP035	H389
BT_1145_Cassidinae sp. 3	Cassidinae sp. 3	Network254	3Cluster219	5Cluster214	75Cluster201	GMYC285	PTP052	H381
BT_1146_Eumolpinae sp. 14	Eumolpinae sp. 14	Network285	3Cluster013	5Cluster014	75Cluster014	GMYC009	PTP016	H420
BT_1147_Alticinae sp. 152	Alticinae sp. 152	Network34	3Cluster226	5Cluster221	75Cluster208	GMYC205	PTP134	H048
BT_1148_Alticinae sp. 96	Alticinae sp. 96	Network85	3Cluster044	5Cluster045	75Cluster045	GMYC051	PTP169	H131
BT_1151_Alticinae sp. 57	Alticinae sp. 57	Network70	3Cluster227	5Cluster222	75Cluster209	GMYC059	PTP159	H110
BT_1152_Alticinae sp. 141	Alticinae sp. 141	Network215	3Cluster228	5Cluster108	75Cluster107	GMYC172	PTP183	H328
BT_1157_Alticinae sp. 149	Alticinae sp. 149	Network188	3Cluster119	5Cluster117	75Cluster116	GMYC091	PTP200	H286
BT_1158_Alticinae sp. 146	Alticinae sp. 146	Network121	3Cluster229	5Cluster223	75Cluster210	GMYC160	PTP184	H189
BT_1160_Alticinae sp. 55	Alticinae sp. 55	Network138	3Cluster230	5Cluster224	75Cluster211	GMYC250	PTP095	H213
BT_1161_Alticinae sp. 143	Alticinae sp. 143	Network123	3Cluster122	5Cluster120	75Cluster119	GMYC158	PTP186	H196
BT_1165_Alticinae sp. 61	Alticinae sp. 61	Network8	3Cluster068	5Cluster069	75Cluster069	GMYC259	PTP066	H008
BT_1166_Alticinae sp. 140	Alticinae sp. 140	Network122	3Cluster153	5Cluster150	75Cluster144	GMYC157	PTP187	H191
BT_1170_Alticinae sp. 105	Alticinae sp. 105	Network16	3Cluster116	5Cluster114	75Cluster113	GMYC234	PTP079	H025
BT_1171_Alticinae sp. 85	Alticinae sp. 85	Network47	3Cluster138	5Cluster136	75Cluster135	GMYC217	PTP104	H070
BT_1175_Alticinae sp. 87	Alticinae sp. 87	Network88	3Cluster028	5Cluster029	75Cluster029	GMYC061	PTP164	H140
BT_1176_Alticinae sp. 52	Alticinae sp. 52	Network33	3Cluster231	5Cluster225	75Cluster212	GMYC199	PTP137	H047
BT_1178_Alticinae sp. 87	Alticinae sp. 87	Network88	3Cluster028	5Cluster029	75Cluster029	GMYC061	PTP164	H140
BT_1179_Alticinae sp. 118	Alticinae sp. 118	Network169	3Cluster155	5Cluster152	75Cluster054	GMYC188	PTP109	H260
BT_1194_Alticinae sp. 87	Alticinae sp. 87	Network88	3Cluster028	5Cluster029	75Cluster029	GMYC061	PTP164	H136
BT_1196_Alticinae sp. 108	Alticinae sp. 108	Network78	3Cluster232	5Cluster226	75Cluster213	GMYC036	PTP145	H120
BT_1197_Alticinae sp. 104	Alticinae sp. 104	Network17	3Cluster101	5Cluster101	75Cluster100	GMYC235	PTP080	H026
BT_1198_Alticinae sp. 131	Alticinae sp. 131	Network164	3Cluster104	5Cluster103	75Cluster102	GMYC237	PTP077	H254
BT_1199_Alticinae sp. 118	Alticinae sp. 118	Network167	3Cluster233	5Cluster227	75Cluster214	GMYC186	PTP110	H258
BT_1205_Alticinae sp. 52	Alticinae sp. 52	Network36	3Cluster234	5Cluster228	75Cluster215	GMYC204	PTP132	H050
BT_1208_Galerucinae sp. 46	Galerucinae sp. 46	Network155	3Cluster026	5Cluster027	75Cluster027	GMYC178	PTP249	H241
BT_1210_Alticinae sp. 64	Alticinae sp. 64	Network75	3Cluster157	5Cluster154	75Cluster101	GMYC042	PTP148	H117
BT_1211_Alticinae sp. 18	Alticinae sp. 18	Network59	3Cluster158	5Cluster155	75Cluster147	GMYC251	PTP067	H095
BT_1212_Alticinae sp. 17	Alticinae sp. 17	Network44	3Cluster161	5Cluster158	75Cluster149	GMYC215	PTP123	H065
BT_1213_Alticinae sp. 96	Alticinae sp. 96	Network85	3Cluster044	5Cluster045	75Cluster045	GMYC051	PTP169	H131
BT_1214_Alticinae sp. 104	Alticinae sp. 104	Network161	3Cluster235	5Cluster229	75Cluster216	GMYC236	PTP078	H249
BT_1215_Alticinae sp. 144	Alticinae sp. 144	Network129	3Cluster236	5Cluster230	75Cluster217	GMYC163	PTP178	H202
BT_1216_Alticinae sp. 49	Alticinae sp. 49	Network141	3Cluster196	5Cluster191	75Cluster179	GMYC032	PTP085	H217
BT_1217_Alticinae sp. 145	Alticinae sp. 145	Network124	3Cluster237	5Cluster231	75Cluster218	GMYC168	PTP191	H197
BT_1219_Alticinae sp. 86	Alticinae sp. 86	Network48	3Cluster074	5Cluster075	75Cluster074	GMYC216	PTP105	H078
BT_1220_Galerucinae sp. 34	Galerucinae sp. 34	Network183	3Cluster037	5Cluster038	75Cluster038	GMYC103	PTP232	H280
BT_1222_Alticinae sp. 52	Alticinae sp. 52	Network36	3Cluster234	5Cluster228	75Cluster215	GMYC204	PTP132	H051
BT_1223_Alticinae sp. 28	Alticinae sp. 28	Network29	3Cluster039	5Cluster040	75Cluster040	GMYC209	PTP129	H042
BT_1224_Galerucinae sp. 75	Galerucinae sp. 75	Network227	3Cluster145	5Cluster143	75Cluster110	GMYC082	PTP219	H347
BT_1225_Eumolpinae sp. 40	Eumolpinae sp. 40	Network257	3Cluster003	5Cluster003	75Cluster003	GMYC001	PTP003	H388
BT_1226_Galerucinae sp. 34	Galerucinae sp. 34	Network183	3Cluster037	5Cluster038	75Cluster038	GMYC103	PTP232	H280
BT_1228_Alticinae sp. 96	Alticinae sp. 96	Network85	3Cluster044	5Cluster045	75Cluster045	GMYC051	PTP169	H131
BT_1230_Alticinae sp. 115	Alticinae sp. 115	Network10	3Cluster030	5Cluster031	75Cluster031	GMYC253	PTP060	H011
BT_1233_Alticinae sp. 52	Alticinae sp. 52	Network35	3Cluster238	5Cluster228	75Cluster215	GMYC203	PTP133	H049
BT_1235_Alticinae sp. 96	Alticinae sp. 96	Network85	3Cluster044	5Cluster045	75Cluster045	GMYC051	PTP169	H129
BT_1240_Eumolpinae sp. 41	Eumolpinae sp. 41	Network256	3Cluster239	5Cluster232	75Cluster219	GMYC029	PTP001	H383
BT_1245_Alticinae sp. 85	Alticinae sp. 85	Network47	3Cluster138	5Cluster136	75Cluster135	GMYC217	PTP104	H072
BT_1249_Alticinae sp. 104	Alticinae sp. 104	Network17	3Cluster101	5Cluster101	75Cluster100	GMYC235	PTP080	H028
BT_1251_Alticinae sp. 131	Alticinae sp. 131	Network164	3Cluster104	5Cluster103	75Cluster102	GMYC237	PTP077	H254
BT_1252_Alticinae sp. 86	Alticinae sp. 86	Network48	3Cluster074	5Cluster075	75Cluster074	GMYC216	PTP105	H079
BT_1258_Galerucinae sp. 28	Galerucinae sp. 28	Network64	3Cluster105	5Cluster104	75Cluster103	GMYC128	PTP283	H103
BT_1259_Galerucinae sp. 049	Galerucinae sp. 049	Network158	3Cluster108	5Cluster107	75Cluster106	GMYC182	PTP244	H245
BT_1260_Eumolpinae sp. 24	Eumolpinae sp. 24	Network279	3Cluster098	5Cluster098	75Cluster097	GMYC014	PTP021	H412
BT_1263_Alticinae sp. 51	Alticinae sp. 51	Network41	3Cluster089	5Cluster089	75Cluster088	GMYC197	PTP126	H058
BT_1264_Alticinae sp. 78	Alticinae sp. 78	Network147	3Cluster240	5Cluster233	75Cluster220	GMYC255	PTP061	H226
BT_1267_Alticinae sp. 128	Alticinae sp. 128	Network91	3Cluster093	5Cluster093	75Cluster092	GMYC055	PTP160	H145
BT_1269_Alticinae sp. 9	Alticinae sp. 9	Network192	3Cluster096	5Cluster096	75Cluster095	GMYC099	PTP196	H299
BT_1270_Galerucinae sp. 28	Galerucinae sp. 28	Network63	3Cluster201	5Cluster196	75Cluster103	GMYC129	PTP282	H100
BT_1271_Alticinae sp. 57	Alticinae sp. 57	Network69	3Cluster241	5Cluster234	75Cluster221	GMYC060	PTP158	H109
BT_1272_Alticinae sp. 128	Alticinae sp. 128	Network89	3Cluster113	5Cluster112	75Cluster111	GMYC054	PTP163	H142
BT_1273_Galerucinae sp. 28	Galerucinae sp. 28	Network64	3Cluster105	5Cluster104	75Cluster103	GMYC128	PTP283	H101
BT_1274_Alticinae sp. 42	Alticinae sp. 42	Network52	3Cluster002	5Cluster002	75Cluster002	GMYC219	PTP114	H084
BT_1275_Alticinae sp. 51	Alticinae sp. 51	Network39	3Cluster242	5Cluster235	75Cluster222	GMYC196	PTP124	H055
BT_1278_Alticinae sp. 143	Alticinae sp. 143	Network123	3Cluster122	5Cluster120	75Cluster119	GMYC158	PTP186	H194
BT_1280_Alticinae sp. 69	Alticinae sp. 69	Network83	3Cluster243	5Cluster236	75Cluster223	GMYC045	PTP154	H126
BT_1281_Galerucinae sp. 47	Galerucinae sp. 47	Network157	3Cluster205	5Cluster200	75Cluster187	GMYC180	PTP242	H243
BT_1282_Galerucinae sp. 52	Galerucinae sp. 52	Network201	3Cluster087	5Cluster087	75Cluster086	GMYC107	PTP231	H310
BT_1283_Galerucinae sp. 6	Galerucinae sp. 6	Network104	3Cluster244	5Cluster237	75Cluster224	GMYC133	PTP256	H163

BT_1284_Galerucinae sp. 63	Galerucinae sp. 63	Network100	3Cluster245	5Cluster238	75Cluster225	GMYC121	PTP269	H157
BT_1286_Alticinae sp. 21	Alticinae sp. 21	Network67	3Cluster246	5Cluster239	75Cluster226	GMYC050	PTP167	H106
BT_1287_Alticinae sp. 67	Alticinae sp. 67	Network80	3Cluster247	5Cluster240	75Cluster227	GMYC058	PTP155	H123
BT_1288_Alticinae sp. 103	Alticinae sp. 103	Network40	3Cluster248	5Cluster241	75Cluster228	GMYC198	PTP125	H056
BT_1293_Alticinae sp. 50	Alticinae sp. 50	Network45	3Cluster094	5Cluster094	75Cluster093	GMYC211	PTP107	H067
BT_1294_Galerucinae sp. 72	Galerucinae sp. 72	Network230	3Cluster118	5Cluster116	75Cluster115	GMYC074	PTP226	H350
BT_1295_Alticinae sp. 57	Alticinae sp. 57	Network70	3Cluster227	5Cluster222	75Cluster209	GMYC059	PTP159	H111
BT_1304_Galerucinae sp. 47	Galerucinae sp. 47	Network157	3Cluster205	5Cluster200	75Cluster187	GMYC180	PTP242	H243
BT_1305_Alticinae sp. 93	Alticinae sp. 93	Network37	3Cluster249	5Cluster242	75Cluster229	GMYC249	PTP091	H052
BT_1307_Eumolpinae sp. 16	Eumolpinae sp. 16	Network286	3Cluster184	5Cluster180	75Cluster168	GMYC002	PTP012	H422
BT_1308_Alticinae sp. 134	Alticinae sp. 134	Network134	3Cluster250	5Cluster243	75Cluster230	GMYC166	PTP176	H208
BT_1310_Alticinae sp. 35	Alticinae sp. 35	Network144	3Cluster251	5Cluster110	75Cluster109	GMYC034	PTP087	H222
BT_1311_Alticinae sp. 69	Alticinae sp. 69	Network82	3Cluster252	5Cluster244	75Cluster231	GMYC056	PTP157	H125
BT_1312_Alticinae sp. 93	Alticinae sp. 93	Network37	3Cluster249	5Cluster242	75Cluster229	GMYC249	PTP091	H052
BT_1318_Eumolpinae sp. 24	Eumolpinae sp. 24	Network279	3Cluster098	5Cluster098	75Cluster097	GMYC014	PTP021	H412
BT_1319_Alticinae sp. 51	Alticinae sp. 51	Network41	3Cluster089	5Cluster089	75Cluster088	GMYC197	PTP126	H058
BT_1321_Galerucinae sp. 61	Galerucinae sp. 61	Network93	3Cluster041	5Cluster042	75Cluster042	GMYC131	PTP279	H149
BT_1322_Alticinae sp. 19	Alticinae sp. 19	Network58	3Cluster218	5Cluster213	75Cluster200	GMYC245	PTP097	H094
BT_1323_Alticinae sp. 104	Alticinae sp. 104	Network161	3Cluster235	5Cluster229	75Cluster216	GMYC236	PTP078	H251
BT_1324_Alticinae sp. 92	Alticinae sp. 92	Network200	3Cluster135	5Cluster133	75Cluster132	GMYC191	PTP208	H309
BT_1326_Eumolpinae sp. 19	Eumolpinae sp. 19	Network265	3Cluster031	5Cluster032	75Cluster032	GMYC028	PTP004	H396
BT_1335_Alticinae sp. 118	Alticinae sp. 118	Network168	3Cluster253	5Cluster245	75Cluster232	GMYC185	PTP111	H259
BT_1340_Galerucinae sp. 66	Galerucinae sp. 66	Network225	3Cluster254	5Cluster246	75Cluster060	GMYC078	PTP223	H344
BT_1349_Alticinae sp. 51	Alticinae sp. 51	Network32	3Cluster164	5Cluster161	75Cluster152	GMYC206	PTP136	H046
BT_1350_Alticinae sp. 131	Alticinae sp. 131	Network164	3Cluster104	5Cluster103	75Cluster102	GMYC237	PTP077	H254
BT_2073_Alticinae sp. 22	Alticinae sp. 22	Network24	3Cluster255	5Cluster247	75Cluster233	GMYC231	PTP075	H036
BT_2074_Galerucinae sp. 34	Galerucinae sp. 34	Network183	3Cluster037	5Cluster038	75Cluster038	GMYC103	PTP232	H280
BT_2076_Alticinae sp. 2	Alticinae sp. 2	Network187	3Cluster010	5Cluster010	75Cluster010	GMYC087	PTP204	H285
BT_2077_Galerucinae sp. 7	Galerucinae sp. 7	Network109	3Cluster019	5Cluster020	75Cluster020	GMYC134	PTP267	H169
BT_2078_Alticinae sp. 265	Alticinae sp. 265	Network60	3Cluster150	5Cluster147	75Cluster143	GMYC068	PTP142	H096
BT_2081_Eumolpinae sp. 42	Eumolpinae sp. 42	Network257	3Cluster003	5Cluster003	75Cluster003	GMYC001	PTP003	H386
BT_2082_Alticinae sp. 64	Alticinae sp. 64	Network72	3Cluster256	5Cluster248	75Cluster234	GMYC043	PTP152	H113
BT_2084_Alticinae sp. 32	Alticinae sp. 32	Network150	3Cluster187	5Cluster182	75Cluster170	GMYC239	PTP084	H233
BT_2085_Eumolpinae sp. 42	Eumolpinae sp. 42	Network257	3Cluster003	5Cluster003	75Cluster003	GMYC001	PTP003	H387
BT_2087_Alticinae sp. 49	Alticinae sp. 49	Network141	3Cluster196	5Cluster191	75Cluster179	GMYC032	PTP085	H218
BT_2090_Alticinae sp. 81	Alticinae sp. 81	Network209	3Cluster084	5Cluster084	75Cluster083	GMYC069	PTP143	H322
BT_2093_Alticinae sp. 96	Alticinae sp. 96	Network85	3Cluster044	5Cluster045	75Cluster045	GMYC051	PTP169	H130
BT_2097_Alticinae sp. 122	Alticinae sp. 122	Network181	3Cluster257	5Cluster249	75Cluster235	GMYC149	PTP174	H278
BT_2101_Alticinae sp. 86	Alticinae sp. 86	Network48	3Cluster074	5Cluster075	75Cluster074	GMYC216	PTP105	H080
BT_2107_Alticinae sp. 85	Alticinae sp. 85	Network48	3Cluster074	5Cluster075	75Cluster074	GMYC216	PTP105	H073
BT_2109_Alticinae sp. 133	Alticinae sp. 133	Network130	3Cluster076	5Cluster077	75Cluster076	GMYC170	PTP192	H203
BT_2113_Alticinae sp. 142	Alticinae sp. 142	Network132	3Cluster081	5Cluster081	75Cluster080	GMYC167	PTP189	H205
BT_2119_Eumolpinae sp. 16	Eumolpinae sp. 16	Network286	3Cluster184	5Cluster180	75Cluster168	GMYC002	PTP012	H421
BT_2123_Alticinae sp. 18	Alticinae sp. 18	Network7	3Cluster258	5Cluster250	75Cluster236	GMYC261	PTP068	H007
BT_2135_Alticinae sp. 122	Alticinae sp. 122	Network181	3Cluster257	5Cluster249	75Cluster235	GMYC149	PTP174	H278
BT_2136_Alticinae sp. 110	Alticinae sp. 110	Network173	3Cluster222	5Cluster217	75Cluster204	GMYC242	PTP207	H268
BT_2138_Alticinae sp. 115	Alticinae sp. 115	Network10	3Cluster030	5Cluster031	75Cluster031	GMYC253	PTP060	H010
BT_2152_Eumolpinae sp. 38	Eumolpinae sp. 38	Network284	3Cluster025	5Cluster026	75Cluster026	GMYC010	PTP017	H418
BT_2154_Galerucinae sp. 17	Galerucinae sp. 17	Network115	3Cluster259	5Cluster251	75Cluster237	GMYC123	PTP278	H180
BT_2155_Alticinae sp. 115	Alticinae sp. 115	Network10	3Cluster030	5Cluster031	75Cluster031	GMYC253	PTP060	H011
BT_2156_Cassidinae sp. 12	Cassidinae sp. 12	Network243	3Cluster149	5Cluster146	75Cluster142	GMYC278	PTP044	H367
BT_2157_Eumolpinae sp. 24	Eumolpinae sp. 24	Network278	3Cluster260	5Cluster252	75Cluster097	GMYC013	PTP020	H411
BT_2158_Alticinae sp. 83	Alticinae sp. 83	Network22	3Cluster083	5Cluster083	75Cluster082	GMYC230	PTP072	H034
BT_2161_Cassidinae sp. 12	Cassidinae sp. 12	Network243	3Cluster149	5Cluster146	75Cluster142	GMYC278	PTP044	H366
BT_2168_Galerucinae sp. 36	Galerucinae sp. 36	Network208	3Cluster143	5Cluster141	75Cluster138	GMYC146	PTP254	H320
BT_2170_Galerucinae sp. 34	Galerucinae sp. 34	Network183	3Cluster037	5Cluster038	75Cluster038	GMYC103	PTP232	H280
BT_2173_Eumolpinae sp. 34	Eumolpinae sp. 34	Network266	3Cluster261	5Cluster253	75Cluster238	GMYC027	PTP030	H397
BT_2176_Galerucinae sp. 43	Galerucinae sp. 43	Network179	3Cluster262	5Cluster254	75Cluster239	GMYC120	PTP274	H276
BT_2179_Alticinae sp. 11	Alticinae sp. 11	Network193	3Cluster263	5Cluster255	75Cluster022	GMYC096	PTP193	H300
BT_2180_Alticinae sp. 75	Alticinae sp. 75	Network149	3Cluster127	5Cluster125	75Cluster124	GMYC240	PTP082	H230
BT_2181_Alticinae sp. 251	Alticinae sp. 251	Network90	3Cluster264	5Cluster256	75Cluster240	GMYC053	PTP161	H143
BT_2182_Galerucinae sp. 29	Galerucinae sp. 29	Network95	3Cluster202	5Cluster197	75Cluster184	GMYC124	PTP276	H151
BT_2189_Hispinae sp. 8	Hispinae sp. 8	Network249	3Cluster265	5Cluster257	75Cluster241	GMYC268	PTP046	H374
BT_2191_Criocerinae sp. 5	Criocerinae sp. 5	Network240	3Cluster266	5Cluster258	75Cluster242	GMYC263	PTP056	H362
BT_2192_Galerucinae sp. 28	Galerucinae sp. 28	Network62	3Cluster267	5Cluster259	75Cluster243	GMYC130	PTP281	H098
BT_2193_Alticinae sp. 76	Alticinae sp. 76	Network27	3Cluster207	5Cluster202	75Cluster189	GMYC210	PTP127	H039
BT_2194_Alticinae sp. 127	Alticinae sp. 127	Network4	3Cluster194	5Cluster189	75Cluster177	GMYC247	PTP092	H004
BT_2196_Alticinae sp. 127	Alticinae sp. 127	Network4	3Cluster194	5Cluster189	75Cluster177	GMYC247	PTP092	H004
BT_2197_Eumolpinae sp. 47	Eumolpinae sp. 47	Network280	3Cluster268	5Cluster260	75Cluster244	GMYC015	PTP022	H414

BT_2208_Alticinae sp. 112	Alticinae sp. 112	Network145	3Cluster224	5Cluster219	75Cluster206	GMYC190	PTP209	H224
BT_2312_Alticinae sp. 42	Alticinae sp. 42	Network52	3Cluster002	5Cluster002	75Cluster002	GMYC219	PTP114	H086
BT_2491_Alticinae sp. 85	Alticinae sp. 85	Network47	3Cluster138	5Cluster136	75Cluster135	GMYC217	PTP104	H070
BT_2492_Alticinae sp. 104	Alticinae sp. 104	Network17	3Cluster101	5Cluster101	75Cluster100	GMYC235	PTP080	H027
BT_2495_Alticinae sp. 243	Alticinae sp. 243	Network54	3Cluster006	5Cluster006	75Cluster006	GMYC220	PTP115	H089
BT_2496_Alticinae sp. 140	Alticinae sp. 140	Network122	3Cluster153	5Cluster150	75Cluster144	GMYC157	PTP187	H192
BT_2498_Alticinae sp. 52	Alticinae sp. 52	Network33	3Cluster231	5Cluster225	75Cluster212	GMYC199	PTP137	H047
BT_2499_Alticinae sp. 118	Alticinae sp. 118	Network170	3Cluster053	5Cluster054	75Cluster054	GMYC187	PTP108	H263
BT_2502_Alticinae sp.160	Alticinae sp.160	Network38	3Cluster051	5Cluster052	75Cluster052	GMYC151	PTP071	H054
BT_2504_Galerucinae sp. 098	Galerucinae sp. 098	Network153	3Cluster269	5Cluster261	75Cluster245	GMYC181	PTP243	H236
BT_2505_Galerucinae sp. 46	Galerucinae sp. 46	Network155	3Cluster026	5Cluster027	75Cluster027	GMYC178	PTP249	H241
BT_2506_Alticinae sp. 47	Alticinae sp. 47	Network198	3Cluster270	5Cluster262	75Cluster246	GMYC150	PTP175	H307
BT_2516_Galerucinae sp. 9	Galerucinae sp. 9	Network101	3Cluster271	5Cluster263	75Cluster247	GMYC142	PTP261	H158
BT_2517_Alticinae sp. 201	Alticinae sp. 201	Network21	3Cluster272	5Cluster264	75Cluster248	GMYC233	PTP076	H032
BT_2518_Alticinae sp. 70	Alticinae sp. 70	Network91	3Cluster093	5Cluster093	75Cluster092	GMYC055	PTP160	H146
BT_2519_Alticinae sp. 153	Alticinae sp. 153	Network46	3Cluster273	5Cluster265	75Cluster249	GMYC218	PTP106	H068
BT_2521_Galerucinae sp. 082	Galerucinae sp. 082	Network182	3Cluster121	5Cluster119	75Cluster118	GMYC106	PTP235	H279
BT_2522_Alticinae sp. 51	Alticinae sp. 51	Network41	3Cluster089	5Cluster089	75Cluster088	GMYC197	PTP126	H057
BT_2523_Alticinae sp. 153	Alticinae sp. 153	Network46	3Cluster273	5Cluster265	75Cluster249	GMYC218	PTP106	H069
BT_2529_Galerucinae sp. 55	Galerucinae sp. 55	Network201	3Cluster087	5Cluster087	75Cluster086	GMYC107	PTP231	H310
BT_2544_Alticinae sp. 97	Alticinae sp. 97	Network13	3Cluster035	5Cluster036	75Cluster036	GMYC258	PTP065	H021
BT_2546_Cassidinae sp. 4	Cassidinae sp. 4	Network253	3Cluster038	5Cluster039	75Cluster039	GMYC284	PTP053	H380
BT_2548_Cassidinae sp. 12	Cassidinae sp. 12	Network243	3Cluster149	5Cluster146	75Cluster142	GMYC278	PTP044	H366
BT_2550_Alticinae sp. 56	Alticinae sp. 56	Network15	3Cluster274	5Cluster266	75Cluster250	GMYC200	PTP138	H023
BT_2572_Alticinae sp. 18	Alticinae sp. 18	Network59	3Cluster158	5Cluster155	75Cluster147	GMYC251	PTP067	H095
BT_2573_Cassidinae sp. 12	Cassidinae sp. 12	Network243	3Cluster149	5Cluster146	75Cluster142	GMYC278	PTP044	H366
BT_2575_Eumolpinae sp. 31	Eumolpinae sp. 31	Network287	3Cluster275	5Cluster267	75Cluster251	GMYC005	PTP013	H423
BT_2576_Alticinae sp. 96	Alticinae sp. 96	Network85	3Cluster044	5Cluster045	75Cluster045	GMYC051	PTP169	H131
BT_2578_Galerucinae sp. 34	Galerucinae sp. 34	Network183	3Cluster037	5Cluster038	75Cluster038	GMYC103	PTP232	H280
BT_2579_Alticinae sp. 115	Alticinae sp. 115	Network10	3Cluster030	5Cluster031	75Cluster031	GMYC253	PTP060	H014
BT_2629_Eumolpinae sp. 29	Eumolpinae sp. 29	Network271	3Cluster117	5Cluster115	75Cluster114	GMYC019	PTP024	H403
BT_2631_Alticinae sp. 135	Alticinae sp. 135	Network126	3Cluster276	5Cluster268	75Cluster252	GMYC173	PTP181	H199
BT_2632_Alticinae sp. 143	Alticinae sp. 143	Network123	3Cluster122	5Cluster120	75Cluster119	GMYC158	PTP186	H195
BT_2637_Eumolpinae sp. 40	Eumolpinae sp. 40	Network257	3Cluster003	5Cluster003	75Cluster003	GMYC001	PTP003	H388
BT_2638_Galerucinae sp. 61	Galerucinae sp. 61	Network93	3Cluster041	5Cluster042	75Cluster042	GMYC131	PTP279	H149
BT_2640_Alticinae sp. 104	Alticinae sp. 104	Network161	3Cluster235	5Cluster229	75Cluster216	GMYC236	PTP078	H250
BT_2641_Alticinae sp. 97	Alticinae sp. 97	Network13	3Cluster035	5Cluster036	75Cluster036	GMYC258	PTP065	H017
BT_2642_Eumolpinae sp. 19	Eumolpinae sp. 19	Network265	3Cluster031	5Cluster032	75Cluster032	GMYC028	PTP004	H396
BT_2643_Alticinae sp. 42	Alticinae sp. 42	Network52	3Cluster002	5Cluster002	75Cluster002	GMYC219	PTP114	H086
BT_2644_Criocerinae sp. 1	Criocerinae sp. 1	Network237	3Cluster071	5Cluster072	75Cluster071	GMYC264	PTP057	H359
BT_2646_Alticinae sp. 96	Alticinae sp. 96	Network85	3Cluster044	5Cluster045	75Cluster045	GMYC051	PTP169	H131
BT_2657_Alticinae sp. 1	Alticinae sp. 1	Network186	3Cluster277	5Cluster269	75Cluster253	GMYC093	PTP199	H284
BT_2658_Alticinae sp. 41	Alticinae sp. 41	Network136	3Cluster223	5Cluster218	75Cluster205	GMYC161	PTP177	H211
BT_2659_Alticinae sp. 86	Alticinae sp. 86	Network48	3Cluster074	5Cluster075	75Cluster074	GMYC216	PTP105	H075
BT_2661_Hispinae sp. 9	Hispinae sp. 9	Network250	3Cluster278	5Cluster270	75Cluster254	GMYC270	PTP047	H375
BT_2662_Alticinae sp. 87	Alticinae sp. 87	Network88	3Cluster028	5Cluster029	75Cluster029	GMYC061	PTP164	H140
BT_2663_Hispinae sp. 5	Hispinae sp. 5	Network246	3Cluster130	5Cluster128	75Cluster127	GMYC287	PTP034	H371
BT_2665_Criocerinae sp. 4	Criocerinae sp. 4	Network239	3Cluster189	5Cluster184	75Cluster172	GMYC262	PTP055	H361
BT_2666_Alticinae sp. 89	Alticinae sp. 89	Network199	3Cluster279	5Cluster271	75Cluster255	GMYC155	PTP172	H308
BT_2670_Alticinae sp. 51	Alticinae sp. 51	Network41	3Cluster089	5Cluster089	75Cluster088	GMYC197	PTP126	H058
BT_2671_Galerucinae sp. 72	Galerucinae sp. 72	Network231	3Cluster118	5Cluster116	75Cluster115	GMYC073	PTP225	H352
BT_2672_Alticinae sp. 51	Alticinae sp. 51	Network41	3Cluster089	5Cluster089	75Cluster088	GMYC197	PTP126	H058
BT_2673_Galerucinae sp. 49	Galerucinae sp. 49	Network159	3Cluster280	5Cluster272	75Cluster256	GMYC184	PTP245	H246
BT_2697_Alticinae sp. 53	Alticinae sp. 53	Network28	3Cluster281	5Cluster273	75Cluster257	GMYC208	PTP128	H040
BT_2698_Eumolpinae sp. 071	Eumolpinae sp. 071	Network276	3Cluster282	5Cluster274	75Cluster258	GMYC016	PTP026	H408
BT_2705_Alticinae sp. 94	Alticinae sp. 94	Network140	3Cluster283	5Cluster275	75Cluster259	GMYC148	PTP173	H216
BT_2707_Alticinae sp. 34	Alticinae sp. 34	Network152	3Cluster284	5Cluster276	75Cluster260	GMYC238	PTP081	H235
